# “Dynamical Docking” of Cyclic Dinuclear Au(I) Bis-N-heterocyclic Complexes Facilitates Their Binding to G-Quadruplexes

**DOI:** 10.1021/acs.inorgchem.2c03041

**Published:** 2022-12-09

**Authors:** Clemens Kaußler, Darren Wragg, Claudia Schmidt, Guillermo Moreno-Alcántar, Christian Jandl, Johannes Stephan, Roland A. Fischer, Stefano Leoni, Angela Casini, Riccardo Bonsignore

**Affiliations:** † Chair of Medicinal and Bioinorganic Chemistry, Department of Chemistry, Technical University of Munich, Lichtenbergstraße 4, Garching b. MünchenD-85748, Germany; ‡ Catalysis Research Center & Department of Chemistry, Technische Universität München, Ernst-Otto-Fischer Str. 1, Garching b. MünchenD-85748, Germany; § Chair of Inorganic and Metal-Organic Chemistry, Department of Chemistry, Technische Universität München, Ernst-Otto-Fischer Str. 1, Garching b. MünchenD-85748, Germany; ∥ School of Chemistry, Cardiff University, Park Place, CardiffCF10 3AT, U.K.; ⊥ Dipartimento di Scienze e Tecnologie Biologiche, Chimiche e Farmaceutiche, Università degli Studi di Palermo, Viale delle Scienze, Edificio 17, Palermo90128, Italy

## Abstract

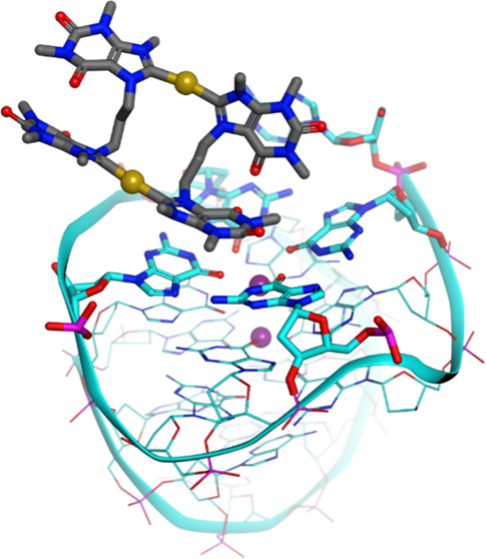

With the aim to improve the design of metal complexes as stabilizers of noncanonical DNA secondary structures, namely, G-quadruplexes (G4s), a series of cyclic dinuclear Au(I) N-heterocyclic carbene complexes based on xanthine and benzimidazole ligands has been synthesized and characterized by various methods, including X-ray diffraction. Fluorescence resonance energy transfer (FRET) and CD DNA melting assays unraveled the compounds’ stabilization properties toward G4s of different topologies of physiological relevance. Initial structure–activity relationships have been identified and recognize the family of xanthine derivatives as those more selective toward G4s versus duplex DNA. The binding modes and free-energy landscape of the most active xanthine derivative (featuring a propyl linker) with the promoter sequence *cKIT1* have been studied by metadynamics. The atomistic simulations evidenced that the Au(I) compound interacts noncovalently with the top G4 tetrad. The theoretical results on the Au(I) complex/DNA Gibbs free energy of binding were experimentally validated by FRET DNA melting assays. The compounds have also been tested for their antiproliferative properties in human cancer cells in vitro, showing generally moderate activity. This study provides further insights into the biological activity of Au(I) organometallics acting via noncovalent interactions and underlines their promise for tunable targeted applications by appropriate chemical modifications.

## Abstract 2

Cyclic dinuclear Au(I) NHCs as selective G4-stabilizers.

## Introduction

In the last few decades, gold-based drugs have been added to the medicinal chemistry toolbox as new therapeutic agents featuring promising antiproliferative effects against cancer cells, bacteria, and protozoa and endowed with different modes of action with respect to classical organic drugs.^1−4^ Interest in antimicrobial gold complexes originated at the end of the 19th century from the work of Koch, reporting on the bacteriostatic activity of K[Au(CN)_2_].^5^ Later on, gold therapy (chrysotherapy) has been widely used against rheumatoid arthritis, including in the form of the oral drug auranofin [triethylphosphine(2,3,4,6-tetra-*O*-acetyl-β-1-d-(thiopyranosato-S)Au(I)] (Ridaura). Early studies on auranofin’s anticancer effects revealed activity levels in vitro comparable to those of the Pt(II) complex cisplatin, leading to a vast number of Au(I) complexes with phosphane ligands being evaluated for their antiproliferative effects in vitro and in vivo.^6^ Recently, auranofin itself has been actively repurposed in pilot trials and clinical studies for anticancer therapy against chronic lymphocytic leukemia and ovarian cancer among others.^7,8^

**Figure 1:**
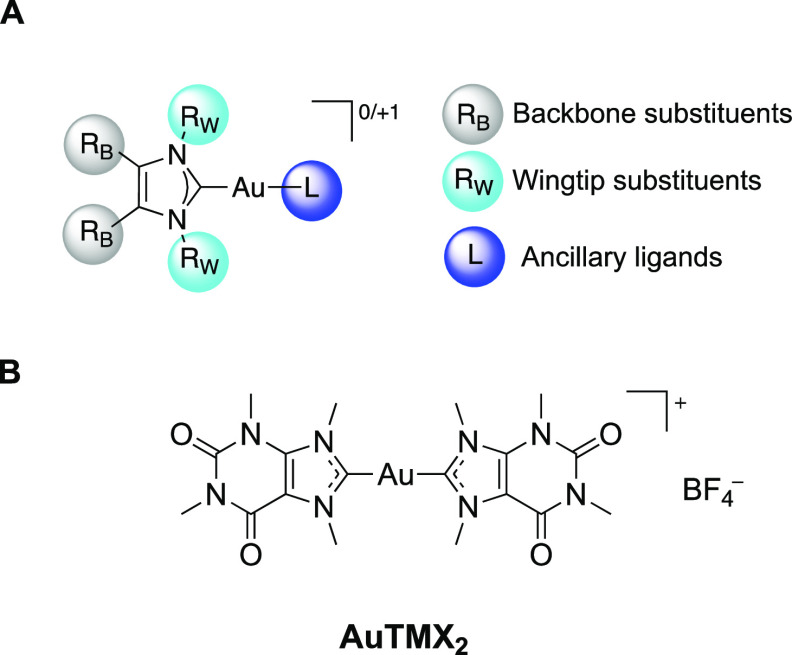
(A) General structure of Au(I) NHC complexes and possible modifications and (B) structure of the cationic bis-NHC Au(I) complex [Au(9-methylcaffeine-8-ylidene)_2_]^+^ (**AuTMX**_**2**_) as a selective G4 stabilizer.^19^

Unfortunately, coordination Au(I) complexes can be easily reduced to colloidal gold in a physiological environment, causing either metallodrug inactivation or toxicity in vivo. To achieve an improved control over metal *speciation* in biological systems, organogold compounds, featuring a stable Au–carbon bond, have been widely explored in medicinal chemistry.^9^ Among the possible organometallic ligands, N-heterocyclic carbenes (NHCs) perfectly fulfill the prerequisites for an optimal drug design and tuning of the metal complex’s reactivity and physicochemical properties, due to their high structural versatility and ease of derivatization. Thus, numerous cytotoxic Au(I) NHC complexes have been synthesized and characterized for their mechanisms of pharmacological action.^10−12^ These studies showed the influence of the NHC scaffold on the anticancer activity of the resulting metal compounds—with, for example, imidazol-2-ylidene carbenes being generally more active than benzimidazol-2-ylidene ones—as well as the role of the functionalization of the NHC backbone, of the wingtip substituents and of the ancillary ligands (Figure 1A) on the resulting metallodrug’s bioactivity.^13−15^ Most of the reported Au(I) NHCs exert their antiproliferative effects via direct binding to protein/enzyme targets, following ligand exchange reactions with thiols or selenol groups in amino acids’ side chains.^16,17^ Positively charged Au(I) NHCs endowed with a high lipophilic character were also observed to selectively target mitochondria in cancer cells,^18^ inducing calcium-sensitive mitochondrial membrane permeabilization accompanied by mitochondrial swelling, as well as by inhibition of mitochondrial enzymes.

In 2014, in collaboration with the group of Le Gendre, some of us reported on the synthesis and antiproliferative properties of xanthine-derived Au(I) NHC complexes as anticancer agents.^19^ Unexpectedly, the compounds were generally inactive as protein binders and enzyme inhibitors, and electrospray ionization mass spectrometry studies (ESI-MS) showed scarce reactivity with amino acids and model proteins.^20^ This observation led to the hypothesis that the compounds’ bioactivity may be based on stable noncovalent adducts with biomolecules. Notably, the lead compound in this series, the cationic caffeine-based bis-NHC Au(I) complex [Au(9-methylcaffeine-8-ylidene)_2_]^+^ (**AuTMX**_**2**_, Figure 1B), emerged as a very effective stabilizer of noncanonical nucleic acid structures, namely, G-quadruplex (G4) DNA. G4s are secondary DNA structures formed in guanine-rich sequences self-assembled by Hoogsteen-type hydrogen bonds and have been identified in human telomeres and promoter regions of many genes, where they regulate telomere homeostasis, gene transcription, and DNA replication.^21^ Stabilization of G4s by small molecules may, therefore, induce anticancer effects due to the resulting inhibition of telomere extensions or oncogene expression.^22^ Metal complexes of different transition metals have been widely investigated as G4 stabilizers and hold promise in drug discovery.^23,24^

Interestingly, **AuTMX**_**2**_ was also endowed with selectivity for G4 structures over duplex B-DNA, at variance with the bis-benzimidazolylidene Au(I) analogue.^19^ The observed selectivity could be rationalized by the ability of the caffeine-ylidene scaffold to mimic the guanines of the G4 framework. Moreover, relativistic Au···π-interactions may also play a role in the interaction of the Au(I) compound with guanines.^25^ According to initial structure–activity relationships^19,26^ and X-ray diffraction (XRD) studies of the adduct formed by **AuTMX**_**2**_ with a model of human telomeric G4 (hTel23),^27^ the compound binds noncovalently between neighboring quadruplexes in the crystal lattice, and the presence of two NHC ligands is essential to achieve the highest possible stabilization of the G4 structure. Further structural characterization of the binding modes of **AuTMX**_**2**_ with different G4s was achieved by advanced atomistic simulations, evidencing the importance of π–π stacking and possibly electrostatic interactions in stabilizing the Au(I) compound/G4 adducts.^28^

In cells, **AuTMX**_**2**_ shows selectivity for cancerous over nontumorigenic cells and against the ovarian cancer A2780 cells with respect to other human cancer cell lines.^19^ Recently, to shed light into the mechanisms of anticancer action of **AuTMX**_**2**_, shotgun proteomics was applied enabling analysis of the global protein expression changes of **AuTMX**_**2**_-treated A2780 cancer cells.^20^ The obtained results, combined with various pharmacological methods, evidenced a multi-modal activity of **AuTMX**_**2**_ based on noncovalent interactions with intracellular targets, and involving alterations in the nucleolus, telomeres, actin stress-fibers, as well as activation of redox-related stress-response.^20^ Considering that, beside humans, putative G4-forming sequences have been found in other mammalian genomes, as well as in yeasts, protozoa,^29,30^ bacteria, and viruses,^31^ this type of organogold complexes, active via noncovalent interactions and targeting secondary nucleic acid structures, could be valuable in other research disease areas. In this context, **AuTMX**_**2**_ and selected derivatives were tested against *Leishmania amazonensis* and *Leishmania braziliensis* in vitro, showing promising effects in both promastigote and amastigote cells.^32^

Intrigued by the peculiar reactivity of **AuTMX**_**2**_ with nucleic acid structures with respect to other cytotoxic metallodrugs, we attempted to improve its G4-stabilizing properties by different strategies. Unfortunately, xanthine-derived Au(I) NHC complexes with different substituents at the N-7 position of the ligand scaffold markedly reduced affinity toward G4 stabilization compared to **AuTMX**_**2**_.^19^ Similarly, functionalization of the N-1 position, as well as substitution of one of the two NHC ligands with alkynyl moieties to achieve neutral complexes, resulted in loss of G4s′ stabilizing effects.^26^ These findings were justified by the occurrence of steric effects in the substituted **AuTMX**_**2**_ analogues, eventually interfering with the noncovalent G4 adduct formation and relative stability; while lack of suitable interactions, either electrostatic or involving the alkynyl ligands with the G-tetrads occurred for the neutral heteroleptic Au(I) derivatives.

**Scheme 1:**
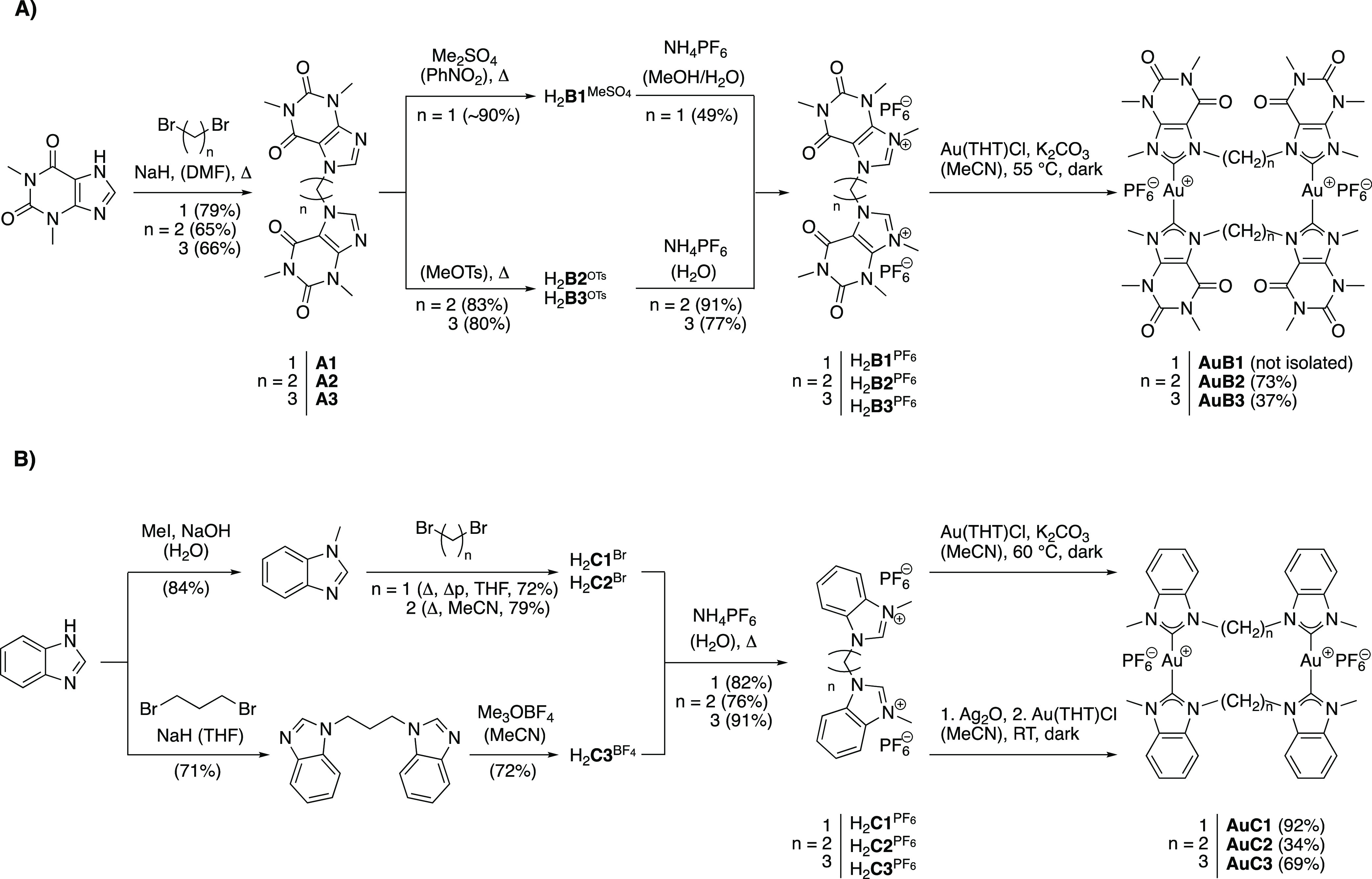
Reaction Scheme Leading to the Metallacyclic Au(I) Purine- (A) and Benzimidazole-Based (B) NHC Complexes

Therefore, to further improve the G4 stabilization properties, we envisioned to “double” the **AuTMX**_**2**_ unit in the compound’s scaffold, designing cyclic dinuclear Au(I) bis-NHC complexes **AuB1–3** in which two purine-derived NHC ligands are coupled by an aliphatic bridge (Scheme 1A). Linkers connecting the two Au(I) bis-NHCs via the wing-tip nitrogens are varied in length to find a balance between steric hindrance and the flexibility required for planarization and efficient π-interaction with G4s. Flexible linkers affect the conformation of the gold macrocycles also with respect to the formation of intramolecular aurophilic interactions.^33^ Moreover, dinuclear gold complexes in which two Au(I) centers are in close proximity, or can at least approach each other through low energy conformational changes, can potentially develop intramolecular aurophilic interactions between the two metal centers, leading to further tuning of the electronic structure and overall compound’s stability and affecting other supramolecular interactions in the proximity of the gold centers.^34^ Unfortunately, only two (**AuB2–3**) out of the three derivatives could be fully isolated and characterized. In addition, three benzimidazole-derived analogues **AuC1–3** (Scheme 1B) were also synthesized for comparison purposes. The five Au(I) compounds have been characterized by different methods, including nuclear magnetic resonance (NMR) spectroscopy, high-resolution electrospray mass spectrometry (HR-ESI-MS), and elemental analysis, as well as by XRD. Moreover, the compounds were studied for their stability in solution and in the presence of *N*-acetyl cysteine as a model biological nucleophile by ^1^H NMR spectroscopy and HR-ESI-MS. The most stable derivatives were then investigated for their G4 DNA stabilizing properties by fluorescence resonance energy transfer (FRET) DNA melting, as well as by circular dichroism (CD), elucidating differences among the two families of Au(I) compounds, particularly in terms of selectivity between G4s and duplex DNA binding affinity in comparison to the benchmark **AuTMX**_**2**_. The binding of the most stabilizing compound within the purine-based cyclic Au(I) bis-NHC family with a promoter G4 sequence (*cKIT1*) was also studied by atomistic simulations, namely, metadynamics (metaD) free energy calculations.^35^ The in silico results confirm and complement the experimental data, providing further structural and energetics information on the ligand binding mechanism, including a quantitatively well-defined free-energy landscape. Finally, preliminary antiproliferative activity assays were conducted in a small panel of human cancer cell lines, as well as in nontumorigenic cells in vitro.

## Results and Discussion

### Synthesis of Cyclic Dinuclear Au(I) NHC Complexes

A detailed path for the synthesis of the cyclic dinuclear Au(I) bis-NHC complexes is reported in Scheme 1 and features three main steps: (i) alkyl-bridging of the NHC ligands, (ii) ligand methylation, and (iii) Au(I) complexation. While imidazole and benzimidazole-based cyclic Au(I) compounds have been already reported in the literature,^36−38^ to the best of our knowledge, the purine-based ligands H_2_**B1–3**^PF6^ have never been synthesized so far. In general, the functionalization of this type of scaffolds is less straightforward with respect to the analogue benzimidazole ligands due to the lower N9 nucleophilicity, requiring an excess of strong electrophiles for substitution reactions.

Starting with the caffeine-based NHC ligands, two theophylline units have been first alkyl-bridged via nucleophilic substitution using dibromo-alkane precursors, leading to **A1–3** in good yields (up to 79%, Scheme 1A). Notwithstanding, the low solubility in polar-aprotic solvents of these compounds hampered their methylation with Meerwein’s salt via previously reported routes.^37^ Inspired by reactions reported by Hori et al. for the solvent-free N9-methylation of xanthines,^39,40^ compounds **A2** and **A3** were finally methylated in molten methyl tosylate at 160 °C (Figures S1 and S2) prior to the anion exchange reaction, which allowed to afford H_2_**B2**^PF6^ and H_2_**B3**^PF6^ in good overall yields of 41 and 49%, respectively (Scheme 1A). The successful theophyllinium ion achievement was also confirmed by ^1^H and ^13^C NMR spectroscopy in CD_3_CN (Figures S3–S9), showing a downfield shift of the C8H and C8 signals up to 8.35 and 8.52 ppm (^1^H) and 140.00 and 139.56 ppm (^13^C), respectively, for H_2_**B2**^PF6^ and H_2_**B3**^PF6^.

These harsh conditions were regrettably not suitable for **A1** methylation, as ^1^H NMR spectroscopy on the crude reaction mixture showed the prevalence of the 9-methylcaffeinium ion, likely forming via fragmentation of the monomethylated intermediate. Following another procedure by Hori et al.,^41^ appropriate **A1**-methylation conditions were achieved using 30 equiv of dimethyl sulfate in dry nitrobenzene at 90 °C (Figure S10). Afterward, the mixture was used without further purification to afford the ligand H_2_**B1**^PF6^ via the counterion exchange reaction in a methanol/water mixture. Complete characterization of the product was carried out using elemental analysis, as well as ^1^H, ^13^C, ^31^P, and ^1^H–^13^C HSQC NMR spectroscopy, the latter showing downfield shifts of the imidazolium proton and carbon signals compared to reagent **A1** (Figures S11–S14).

Concerning benzimidazole-based scaffolds, the imidazolium ligands H_2_**C1–3** (Scheme 1B) have already been reported by Kühn’s group in 2010, with their synthesis involving harsh conditions such as a pressure tube at 120 °C.^42^ While the methylene-bridged ligand H_2_**C1**^Br^ could only be synthesized via this methodology, a diverse approach was used to afford H_2_**C2**^Br^ and H_2_**C3**^BF4^. While H_2_**C2**^Br^ could be obtained by refluxing 1-methyl benzimidazole with the linking agent in acetonitrile, the pathway to the propyl-bridged ligand (H_2_**C3**^BF4^) had to be inverted, whereby the linking of the two benzimidazole units had to occur prior to their methylation (Figures S15–S17). As for the caffeine-based scaffolds, a further counterion exchange reaction allowed one to isolate the PF_6_ salts of the ligands (H_2_**C1–3**^PF6^) in moderate to good yields, with their analytical data matching the literature.^42^

To afford most of the cyclic Au(I) bis-NHC complexes, except for derivatives **AuB1** and **AuC2**, a so-called “weak-base approach” was applied (Scheme 1).^43^ A protocol by Nolan and coworkers^43^ was slightly modified by replacing the use of acetone with acetonitrile to circumvent solubility issues of the NHC ligands. In the presence of K_2_CO_3_, the reactions to **AuB2**, **AuB3**, **AuC1**, and **AuC3** proceeded to completion at ca. 60 °C within a day. Of note, while **AuC3** was previously synthesized by Tubaro and coworkers from the bromine salt of the ligand (H_2_**C3**^Br^),^36^ in our case milder temperature conditions were required using the hexafluorophosphate salt.

As this approach failed to afford the other two complexes, **AuB1** and **AuC2**, in situ transmetalation of silver carbene was attempted, following a procedure recently reported by us.^26^ Notwithstanding, only the benzimidazole derivative **AuC2** was successfully isolated upon reaction, while several unidentified side products emerged during the reaction to afford **AuB1**, unfortunately impeding its isolation. Complete characterization of the cyclic Au(I) bis-NHC complexes was achieved by high-resolution mass spectrometry, elemental analysis, and NMR spectroscopy, the latter clearly showing the disappearance of the carbenic proton upon Au(I) complexation (Figures S18–S35 in the Supporting Information).

### X-ray Structural Characterization

**Figure 2:**
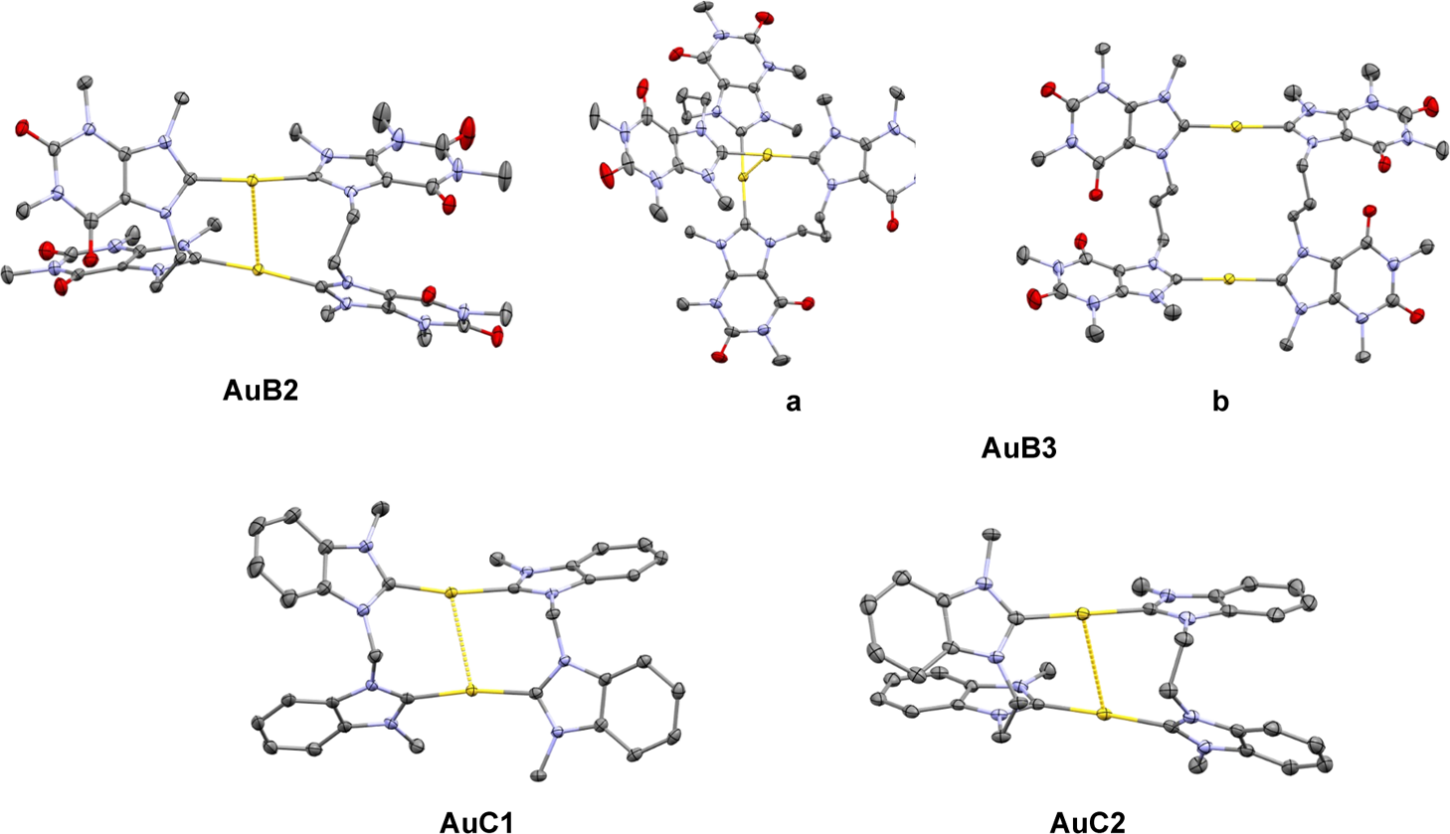
Molecular structures of the cyclic dinuclear Au(I) bis-NHC complexes **AuB2**, **AuB3**, **AuC1**, and **AuC2**. Compound **AuB3** features two independent molecules (a,b) with different conformations. Ellipsoids are displayed at the 50% probability level. Hydrogen atoms as well as co-crystallized solvent molecules and counterions are omitted for clarity. Color code: Au (yellow), C (gray), N (blue), and O (red).

Crystals of **AuB2**, **AuB3**, and **AuC1** (Figure 2) were grown by slow diffusion of diethyl ether in saturated dimethylformamide (**AuB2** and **AuB3**) or acetonitrile (**AuC1**) solutions, while **AuC2** crystals (Figure 2) grew upon slow evaporation of saturated acetonitrile solutions. All of their structures were determined by XRD (Figure 2, Table 1 and S1–S5 in the Supporting Information), whereas the structure of **AuC3** was already reported in the literature.^36^ The Au–carbene bond lengths (see Table 1 for selected bond lengths and angles) are between 2.016(5) and 2.028(4) Å, in the typical range of NHC–Au(I) complexes found in the Cambridge Structural Database (CSD).^44,45^ The C–Au–C angles deviate from perfect linearity by different degrees [range from 168.73(9) to 177.68(14)°] but are within literature-reported values for dinuclear Au(I) complexes of bis-NHC ligands. A survey of CSD data shows that deviations from 180° by around 8° or more do basically not occur when monodentate NHC ligands are involved but instead are frequently observed with bidentate or multidentate NHC ligands, which can be attributed to the strain of the ligands and intramolecular aurophilic interactions.^44,45^ With intramolecular Au···Au distances between 2.9188(7) and 3.2118(4) Å, strong aurophilic interactions can be observed in all compounds with a fold back conformation.^46^ Intriguingly, the **AuB3** crystal structure features two independent molecules with different conformations (Figure 2). Only the first one (Figure 2a), with a twisted conformation resembling a cutout from a helix, features an aurophilic interaction—the shortest one [2.9188(7) Å] among all our compounds. A similar arrangement was reported for a macrocyclic benzimidazole-based *tetra*-NHC complex of Ag.^47^ This structure is the only one in our study in which the two NHCs binding one Au(I) center are not strongly rotated around the C–Au–C axis; instead, they are bent outward with angles of 23.11 and 30.68° between each other, while the two NHC planes of the same ligand are roughly parallel (angles of 3.57 and 13.00°).

**Table 1:** Selected Distances [Å] and Angles [deg] for the Complexes Depicted in Figure 2 (**AuB3** Has Two Independent Molecules in the Asymmetric Unit, a and b as Shown in Figure 2)^a^

	**AuB2**	**AuB3** (a)	**AuB3** (b)	**AuC1**	**AuC2**
Au–C	2.023(4)	2.021(5)	2.016(5)	2.013(2)	2.027(3)
	2.028(4)	2.020(5)		2.019(2)	2.022(3)
	2.024(4)	2.023(5)		2.024(2)	2.022(3)
	2.020(4)	2.021(5)		2.021(2)	2.027(3)
Au–Au	3.1307(5)	2.9188(7)	5.7990(8)	3.027(1)	3.2118(4)
C–Au–C	170.58(18)	171.38(19)	176.10(19)	170.03(9)	177.68(14)
	175.59(19)	172.76(19)		168.73(9)	176.22(14)
NHC–NHC (via Au)	40.90	23.11	63.10	55.84	46.49
	53.12	30.68		53.46	59.25
NHC–NHC (via bridge)	69.76	13.00	63.10	80.13	41.58
	47.83	3.57		72.28	54.16
C_carbene_–N_bridge_	1.336(5)	1.331(6)	1.337(6)	1.365(3)	1.371(4)
	1.342(6)	1.336(6)	1.337(6)	1.358(3)	1.355(5)
	1.338(6)	1.332(6)		1.358(3)	1.355(5)
	1.346(5)	1.341(7)		1.360(3)	1.365(5)
C_carbene_–N_terminal_	1.366(5)	1.373(6)	1.361(6)	1.347(3)	1.345(4)
	1.377(5)	1.369(6)	1.366(6)	1.343(3)	1.354(5)
	1.367(5)	1.371(6)		1.344(3)	1.358(5)
	1.369(5)	1.374(7)		1.343(3)	1.344(4)

a NHC–NHC angles are based on mean planes through the five atoms of the respective imidazole rings and the smaller angle between the planes is always chosen.

In the second structure of **AuB3** (Figure 2b), the Au atoms are apart by 5.7990(8) Å and the conformation can be described as outstretched with a slightly folded bridge, whereas the literature-reported **AuC3** has a fully outstretched bridge with a Au···Au distance of 6.827(2) Å.^36^ Moreover, in the second molecule of **AuB3** and in the other complexes, the angles between the NHC planes of the same ligand are much larger (>40°, Table 1). Particularly in the ethylene-bridged complexes, the NHC moieties are heavily folded toward each other. Compared to the out-of-plane bending in the first molecule of **AuB3**; in the other cases, the NHCs bound to the same Au atom are rotated by 40.90–63.10° (Table 1) around the C–Au–C axis. This is probably imposed by the limited flexibility of the bidentate ligands as *di*-NHC Au complexes of monodentate NHC ligands usually favor a coplanar arrangement (within tolerance and in the absence of bulky wingtips), as reported, for example, in **AuTMX**_**2**_ or [Au(dimethylbenzimidazolylidene)_2_]^+^ with different counterions.^48,49^

It has been previously shown that the C_carbene_–N bonds in **AuTMX**_**2**_ and related compounds are of different lengths, which was rationalized by consideration of the mesomeric structures of disubstituted theophyllinium ions, suggesting that the bond order of the C–N bond closer to the adjacent carbonyl group of the pyrimidine ring is higher and corresponding to a shorter bond length by ca. 0.03 Å.^19^ In accordance with this observation, very similar differences in the C_carbene_–N bond lengths of **AuB2** and **AuB3** were noticed (Table 1). The benzimidazole-derived complexes **AuC1** and **AuC2** as well as literature-reported **AuC3**,^36^ however, do not feature such systematic differences but instead display smaller variations or no differences within standard deviations at all.

### Reactivity with Model Thiols

**Figure 3:**
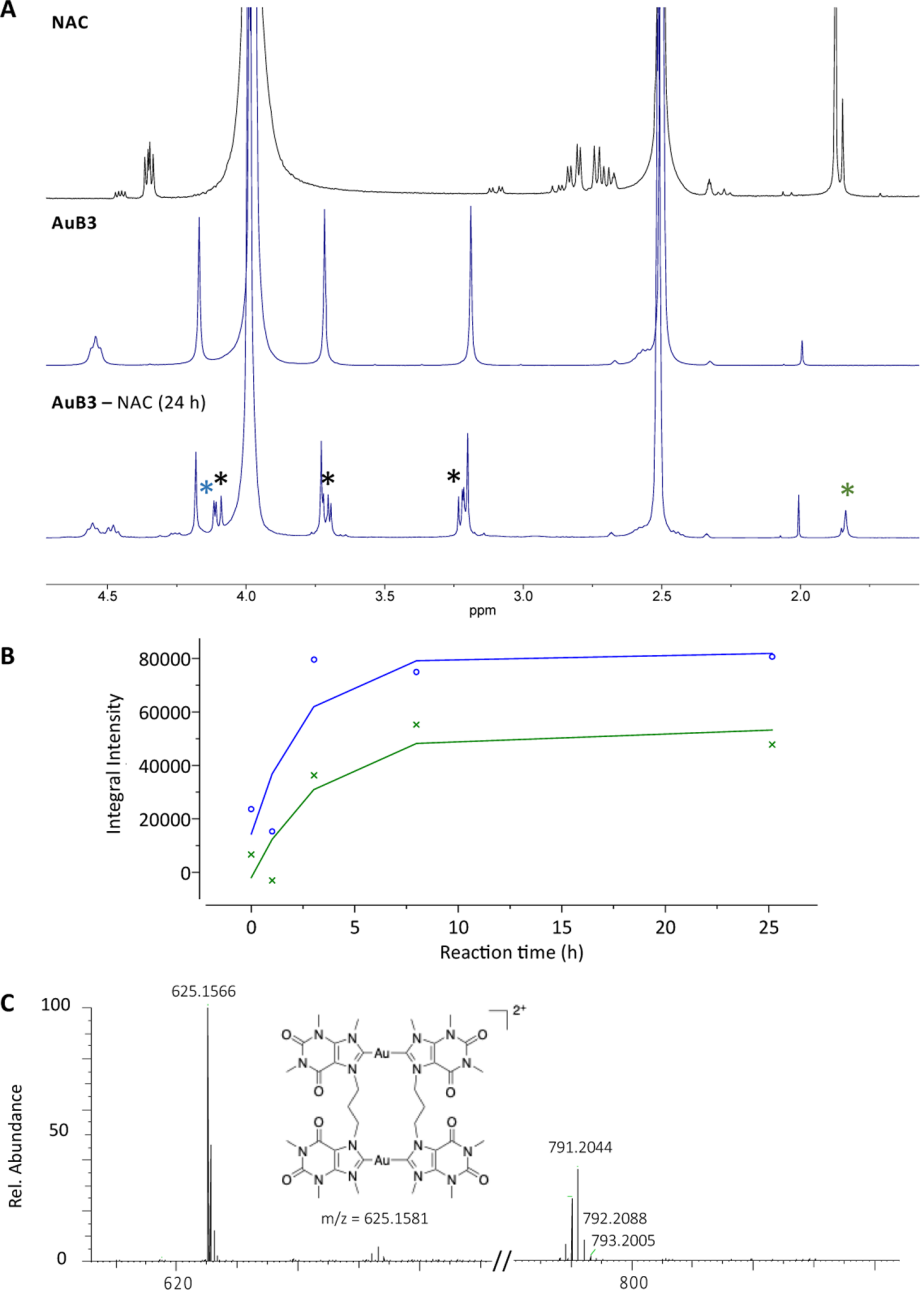
(A) ^1^H NMR spectra in DMSO-*d*_6_/D_2_O (80:20) of **AuB3** alone and in the presence of an equimolar amount of NAC; the NAC spectrum is also reported as reference. Newly formed peaks are labeled with *. (B) Evolution of the 4.10 (blue) and 1.83 ppm ^1^H NMR signals (green) over 24 h reaction. (C) HR-ESI-MS of the reaction mixture after 24 h.

The stability of the newly synthesized Au(I) NHC compounds was assessed over 24 h in a mixture DMSO-*d*_6_/D_2_O (80:20) by ^1^H NMR spectroscopy, showing no spectral changes in this timeframe (Figures S36–S40 in the Supporting Information). To further characterize the compounds’ reactivity toward model biological nucleophiles, each complex was dissolved in a mixture DMSO-*d*_6_/D_2_O (80:20) and exposed for 24 h to an equimolar amount of *N*-acetyl-l-cysteine (NAC), with the reaction monitored via ^1^H NMR spectroscopy. It should be noted that, in the applied experimental conditions, NAC undergoes auto-oxidation to cystine over time with the appearance of new peaks at around 4.45, 3.09, and 1.87 ppm (see reference spectrum in Figure 3A), as already reported in the literature.^26^

While **AuB2**, **AuC2**, and **AuC3** (Figures S41, S44 and S45 in the Supporting Information) did not show any reactivity toward the model nucleophile, significant changes in the ^1^H NMR spectra were recorded for **AuB3** over time (Figure 3). The compound is stable over ca. 3 h; afterward, a new multiplet is detected at around 4.5 ppm (blue star in Figure 3A, close to the signal belonging to the aliphatic linking chain of the unreacted complex), which reaches its maximum at 8 h (Figure 3B) and stays stable over 24 h, while additional new peaks appear in the range 3.0–4.1 ppm. Similarly, a new signal is detected at 1.83 ppm, in the region where NAC resonates (green star in Figure 3A), whereas no further peaks appear in the aromatic range, suggesting that no protonation of the carbenic carbon occurs. Concerning complex **AuC1**, new peaks can be detected after NAC addition whose intensity increases over time (Figure S42). After 24 h, the methylene-bridged benzimidazole-based Au(I) complex, **AuC1**, showed a new multiplet of low intensity between 8.16 and 8.23 ppm and in the high-field range, two more peaks appeared at 1.79 and 1.56 ppm, respectively, while NAC signals varied their intensity over time (Figure S42). Such variations are likely to correspond to NAC–Au adduct formation; nevertheless, the main species present corresponds to the intact Au(I) complex even after 24 h.

To further elucidate the nature of the new species formed, the **AuB3**–NAC reaction mixture was analyzed via HR-ESI-MS in comparison to the sample of the unreactive analogue **AuC3** (Figures 3C and S43). While the latter, as expected, displayed only the species corresponding to the intact gold compound, the **AuB3**–NAC sample spectrum shows both the signal of the intact complex and an additional monocharged peak at 791.2044 *m*/*z*, which could not be identified. Overall, these results indicate that the five cyclic compounds are sufficiently stable in solution and even when forming adducts with biological nucleophiles, their reactivity is markedly slower with respect to other Au(I) NHC systems.

### DNA G4-Stabilization Properties

The G4-stabilizing properties of the isolated bis-NHC Au(I) complexes, **AuB2**, **AuB3**, and **AuC1–3**, were assessed via the FRET DNA melting assay using a panel of five G4s in a 5:1 stoichiometry [Au(I) NHC complex: G4, see the Experimental Section for details]. The G4s were chosen to represent both the telomeric (hTelo) and promoter (*cKIT1*, *cKIT2*, *BCL2*, and *hTERT*) regions of the polynucleotide, as well as different G4 strand orientations such as hybrid-mixed (3 + 1) (hTelo and *BCL2*) and parallel (*cKIT1*, *cKIT2*, and *hTERT*).^50−52^ The bis-caffeine complex **AuTMX**_**2**_ was tested as a benchmark, also because its *cKIT2* and *BCL2* G4-stabilizing properties have never been reported so far.^26,28^

**Figure 4:**
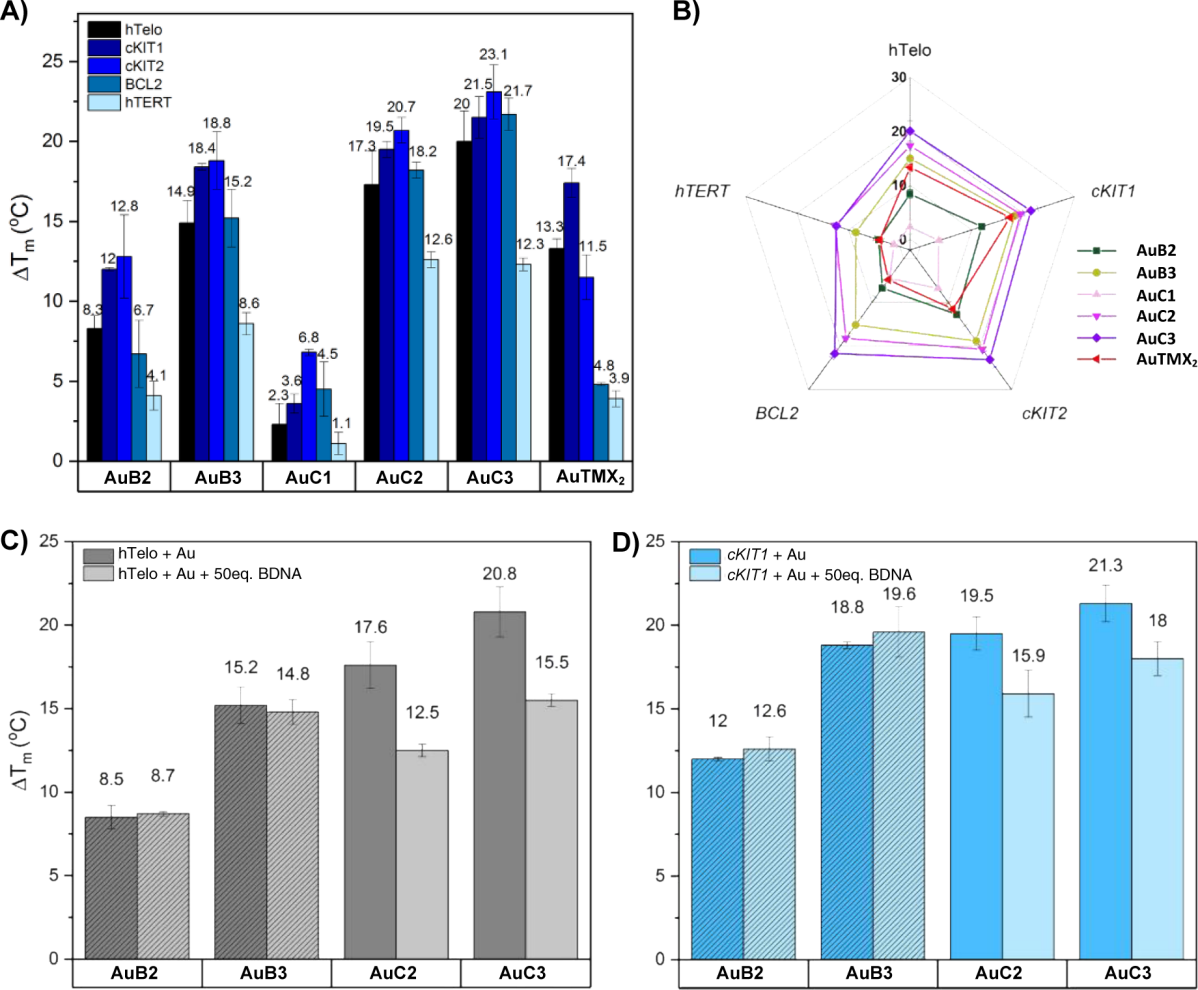
G-quadruplex stabilization effects induced by the Au(I) NHC complexes studied by the FRET DNA melting assay. (a) Δ*T*_m_ (°C) of selected G4s solutions in 60 mM potassium cacodylate (pH = 7.4) in the presence of Au(I) NHC complexes (5 equiv); (b) representation of the Δ*T*_m_ (°C) in a radial plot (from 0 to 30 °C). Δ*T*_m_ (°C) of hTelo (c) and *cKIT1* (d) samples in 60 mM potassium cacodylate (pH = 7.4) in the presence of 5 equiv of selected Au(I) NHC complexes and 50 equiv of CT-DNA. A pattern is shown to differentiate the purine series from the benzimidazole one. Data are shown as mean ± SEM of at least three independent experiments.

As shown in Figure 4A,B, all the Au(I) bis-NHC complexes can stabilize the selected panel of G4s, with their Δ*T*_m_ increasing with the length of the linker chain. This trend is particularly evident within the xanthine-based complex series, with the propyl-bridged Au(I) NHC complex **AuB3** outperforming the shorter bridged **AuB2** by ca. 6 °C per G4 structure. Similarly, in the benzimidazole series, **AuC3** displays a ca. 2–3 °C higher Δ*T*_m_ than **AuC2**, while the methylene-bridged complex **AuC1** barely stabilizes the selected G4s (with Δ*T*_m_ ranging from 1.1 to a max of 6.8 °C depending on the G4). In the xanthine series, **AuB3** shows a comparable stabilizing capability compared to the benchmark **AuTMX**_**2**_ toward hTelo and *cKIT1* but higher Δ*T*_m_ compared to the other G4s. Specifically, **AuB3** features larger stabilization effects than **AuTMX**_**2**_ for *cKIT2* and *hTERT* (2-fold increase) and *BCL2* (3-fold increase). In the benzimidazole series, both **AuC2** and **AuC3** can stabilize each G4 structure more prominently than the **AuTMX**_**2**_, with an average increase in Δ*T*_m_ (between 12.3 and 23.1 °C depending on the G4).

In general, a trend of stabilization capability toward the different G4s could be observed. Figure 4B shows that, while **AuTMX**_**2**_ features a certain degree of selectivity, poorly stabilizing *BCL2* and *hTERT* with respect to other G4s, in the bimetallic complexes a comparable stabilization of the different secondary structures occurs. Noteworthy, the stabilization enhancement toward *BCL2* by **AuB3** compared to **AuTMX**_**2**_ represents an interesting element of novelty and deserves further investigation due to its role in cancer: *BCL2* indeed encodes for an antiapoptotic protein, whose overexpression greatly contributes to cancer development and resistance to chemotherapeutic drugs.^53^ Additionally, while the benzimidazole-based complexes show no preference between the different G4s, **AuB2** and **AuB3** induce a 4 °C higher stabilization of *cKIT1* and *cKIT2* compared to the investigated hybrid-mixed conformations, highlighting a mild preference toward parallel G4 structures. To further validate the trend of stabilization via another method, to exclude the influence of the FRET fluorochromes in the binding, we decided to perform additional CD DNA melting studies on selected compounds, namely, the most active **AuB2**, **AuB3**, and **AuC3** derivatives. The results are reported in Figure S47 and show that the compounds feature the same range and trend of Δ*T*_m_ values as assessed by FRET with **AuB2** < **AuB3** < **AuC3**.

Afterward, the two series of cyclic Au(I) bis-NHC complexes were challenged with an excess (50 equiv) of duplex *calf-thymus* DNA (CT-DNA) in the presence of hTelo or *cKIT1*, chosen as models for hybrid and parallel mimicking G4s, respectively. Both the benzimidazole-based compounds, **AuC2** and **AuC3**, experienced a loss in stabilization by ca. 28%, while neither **AuB2** nor **AuB3** were affected by the presence of the CT-DNA (Figure 4C,D). The results are in line with the high G4 selectivity observed in the case of **AuTMX**_**2**_ and of previously reported mononuclear N1-substituted caffeine-based Au(I) NHC complexes.^26^ Therefore, although the two benzimidazole-based complexes could stabilize the G4s to a greater extent than **AuTMX**_**2**_, they are less selective DNA binders as their mononuclear Au(I) bis-(*N*,*N*-dimethyl-benzimidazole) derivative.^19^ Further studies are necessary to establish selectivity for G4 over duplex DNA in more physiologically relevant conditions (e.g., higher excess of duplex DNA).

**Figure 5:**
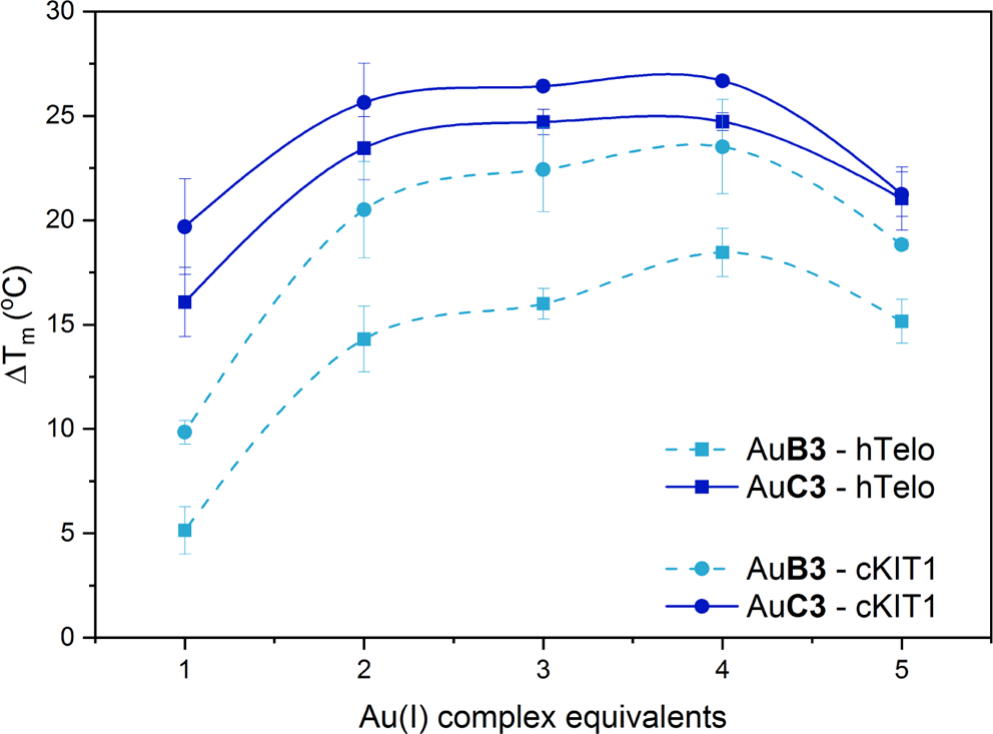
Stabilization effects induced by 1–5 equiv of **AuB3** (sky blue dashed lines) or **AuC3** (dark blue dashed lines) complexes toward hTelo (squares) or *cKIT1* (circles) studied by FRET DNA melting in 60 mM potassium cacodylate (pH = 7.4).

Additional FRET DNA melting assays were performed with increasing equivalents (from 1 to 5 equiv) of the two most potent representative Au(I) complexes in both families, **AuB3** and **AuC3**, using hTelo and *cKIT1* as models. The obtained results are depicted in Figure 5 and show that the system reaches a maximum in stabilization at an Au(I) complex/G4 ratio of 2:1 in each case. Moreover, the overall trend of stabilization is always more pronounced for the benzimidazole derivatives with respect to the caffeine-based analogues.

### CD Titrations

**Figure 6:**
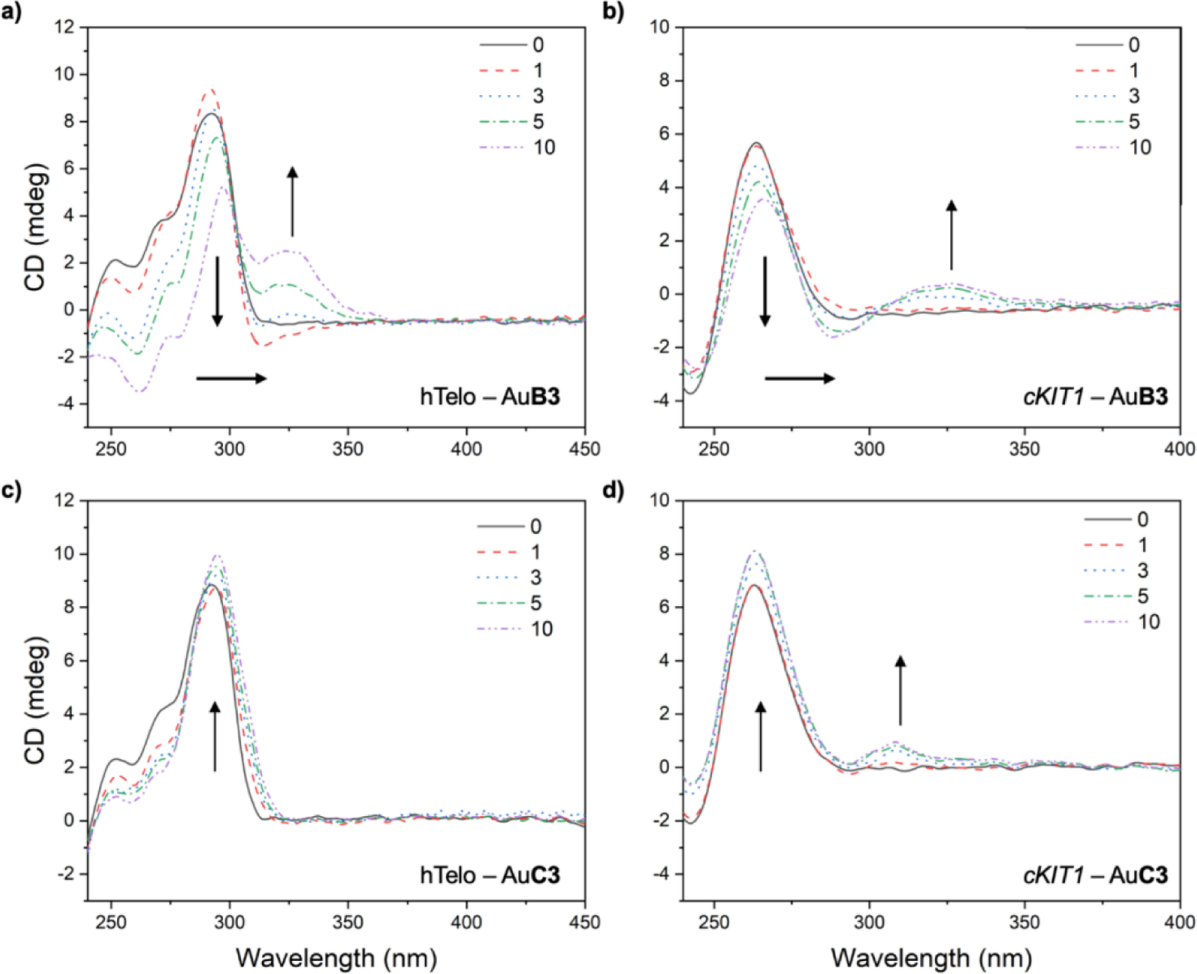
CD spectra of hTelo (a,c) and *cKIT1* solutions (b,d) in the presence of increasing amounts (1–10 equiv) of **AuB3** (a,b) and **AuC3** (c,d) recorded in Tris–HCl/KCl (10/50 mM, pH = 7.4) buffer. Directions of spectral changes are shown by the arrows.

To gain insights into the structural modifications occurring to the G4s upon interaction with the cyclic Au(I) bis-NHC complexes, CD titrations of solution of hTelo and *cKIT1* in the presence of increasing amounts of the most active G4 stabilizers of the two families, **AuB3** and **AuC3**, were performed (Figure 6). As expected, the two G4s have a different conformation resulting in markedly different CD spectra (black traces, Figure 6). The hybrid (3 + 1) hTelo profile is characterized by a positive band at 290 nm, a shoulder at 270 nm, and a negative band at 240 nm (black solid lines in Figure 6A,C), while the parallel G4 folding of *cKIT1* produces an intense positive and a weaker negative band at 263 and 240 nm, respectively (black solid lines in Figure 6B,D).^54^

Increasing amounts (1–10 equiv) of gold complexes result in the appearance of weak-induced CD bands (ICD) above 300 nm in most cases (made exception of the hTelo-**AuC3** adduct), diagnostics of the interaction of the achiral cyclic Au(I) bis-NHC complexes with the chiral G4 backbone, giving rise to new chromophores.^55^ These bands, reported also for other G4-interacting metal complexes,^56,57^ suggest the tight binding of the gold compounds to the DNA secondary structures. Moreover, while groove-binders commonly produce intense ICD signals, DNA intercalators and G4 end-stackers cause weak or absent induced bands.^55^ Considering the moderate magnitude of these bands in our experiments, it is reasonable to assume that the **AuB3** and **AuC3** likely interact with the G-quadruplexes by π-stacking onto the G4-tetrad.

Additionally, the presence of the purine-based representative complex **AuB3** also results in a bathochromic effect and intensity loss of the positive bands for both G4s (Figure 6A,B). Conversely, **AuC3** leads to a moderate increase in the absorbance of the positive peaks of both hTelo and *cKIT1*, located at 290 or 263 nm, respectively (Figure 6C,D). It should be noted that these latter effects and spectral changes in the region <300 nm may also be due to an additive effect of further ICD bands of the complexes. Intriguingly, the hTelo CD spectrum in the presence of **AuC3** does not show any ICD signal and yet experiences the loss of the DNA “shoulder” band located at 270 nm (Figure 6C), which is not the case when **AuB3** is concerned (Figure 6A). This event, together with the increase of the 290 nm band, suggests a likely transition from a hybrid mixed to an antiparallel hTelo conformation in the presence of **AuC3**, as already described for other metal complexes in the literature.^56,58^

### MetaD Study of Noncovalent Adduct Formation

Afterwards, metadymanics was applied to investigate the interactions between di-nuclear **AuB3** and *cKIT1* in comparison with the mono-nuclear **AuTMX**_**2**_ complex previously reported.^28^ MetaD broadens the scope of molecular dynamics simulations by accelerating rare events along selected reaction coordinates, named collective variables (CV), from which the free energy of complex molecular systems^59^ can be integrated. It has been successfully applied to calculate the free-energy surface (FES) of noncovalent substrate/drug adducts with biomolecules,^60,61^ including DNA secondary structures.^28^

**Figure 7:**
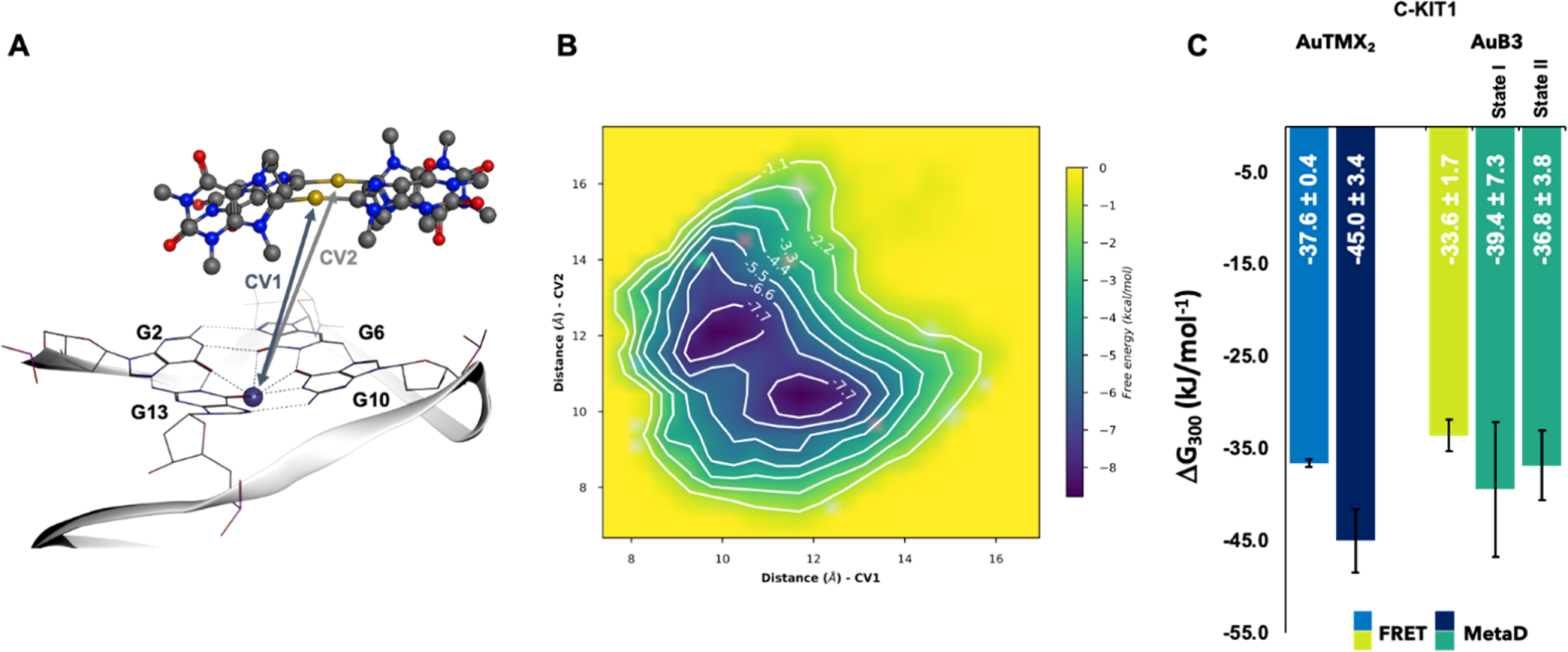
(A) Position of **AuB3** and *cKIT1* at the beginning of the metaD simulation, showing in stick the upper tetrad and upper K^+^ ion (purple sphere) and in ball-and-stick the cyclic dinuclear Au(I) bis-NHC complex. CVs (CV1 and CV2) correspond to distances of Au^+^ ions from the K^+^ ion (Å). (B) Representative FES depicted as a heat map showing two energy minima based on the two CV distances. (C) Comparison of averaged Δ*G* for both metaD and FRET DNA melting assays for **AuTMX**_**2**_(28) and **AuB3**, defined as a bar graph with standard deviation added (state I and state II refer to CV1 < CV2 and CV2 < CV1 respectively).

Well-tempered metaD calculations consisted of manifold 1000 ns (1 ms) of simulation time. Distances between each **AuB3** gold atom and the upper K^+^ ion at the center of *cKIT1*, respectively, were chosen as CVs (CV1 and CV2 in Figure 7A). A FES was obtained for each run. A representative FES heat map in shown Figure 7B, see the Experimental Section for details.

**Figure 8:**
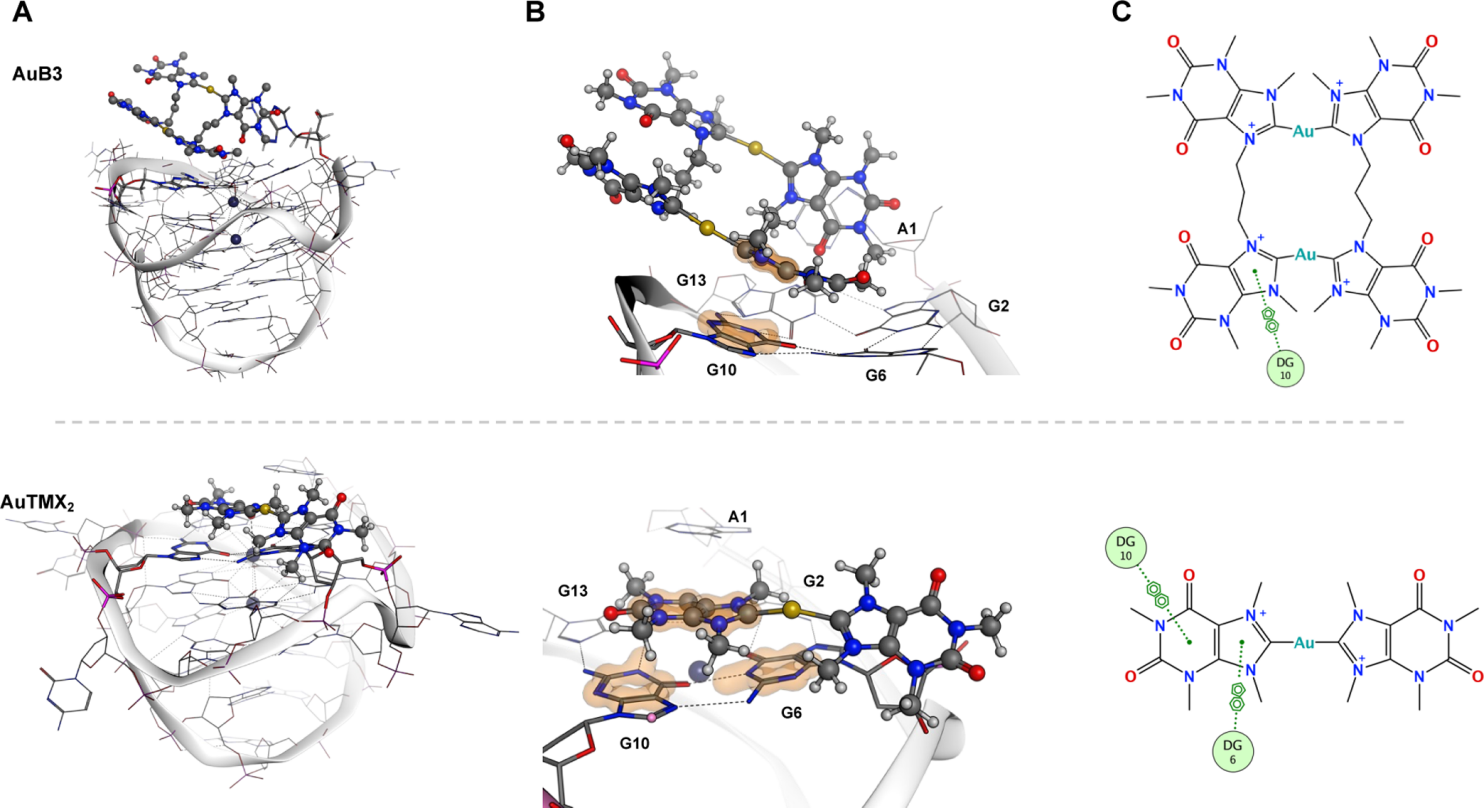
Positions and interactions of the upper G-tetrad nucleobases of *cKIT1* with **AuB3** (top) and **AuTMX**_**2**_ (bottom) as studied by metaD. (A) G4-adducts of **AuB3** and **AuTMX**_**2**_ with *cKIT1* in their lowest energy conformations (state I in the case of **AuB3**). (B) Zoom into the interactions of **AuB3** and **AuTMX**_**2**_ with the upper G4 tetrad with π–π stacking interactions shown as an orange surface. (C) 2D interaction diagram shows increased π–π stacking with **AuTMX**_**2**_ when compared to **AuB3** with nucleobases G6 (DG6) and G10 (DG10).

Gibbs free energy differences and distances were defined with respect to the lowest Δ*G* value of the FES at the corresponding complex-target distance, for each run (values are detailed in Table S6). Figure 7C reports the Δ*G* values calculated for **AuB3** in comparison to those previously reported for the caffeine-based benchmark **AuTMX**_**2**_.^28^ In the CV representation (Figure 7B, heatmap), two free energy minima are visible (see dark blue minima in Figure 7B), which correspond to two equally probable binding states (state I and state II) of comparable energy, namely, −39 ± 7.3 and −36.8 ± 3.8 kJ/mol^–1^, respectively (Figure 7C). Of note, the results are in line with the experimentally calculated Δ*G* values from FRET DNA melting experiments (see Figure 7C and experimental for details). Moreover, they are also comparable to those previously reported for **AuTMX**_**2**_(28) and in line with the experimental observation that the two complexes feature similar G4 stabilizing activity toward *cKIT1* (Figure 4). Overall, from detailed inspection of the simulations, only one caffeine unit (TMX) in **AuB3** interacts closely with guanine bases (G6 and G10) of the upper tetrad region (Table S6, Movie S1 and Figure 8) despite the “doubling” of the **AuTMX**_**2**_ scaffold. More specifically, hydrogen bonding and π–π stacking/π-alkyl interactions with G6 and G10 of the tetrad and the loop base **A1** were the most prevalent (79% of the trajectories) (Figure 8), confirming the top stacking of the complex as surmised from the ICD band appearance. To a lesser extent, interactions were also observed with the other two guanine bases of the top tetrad, G2 (58%) and G13 (42%), respectively.

**Figure 9:**
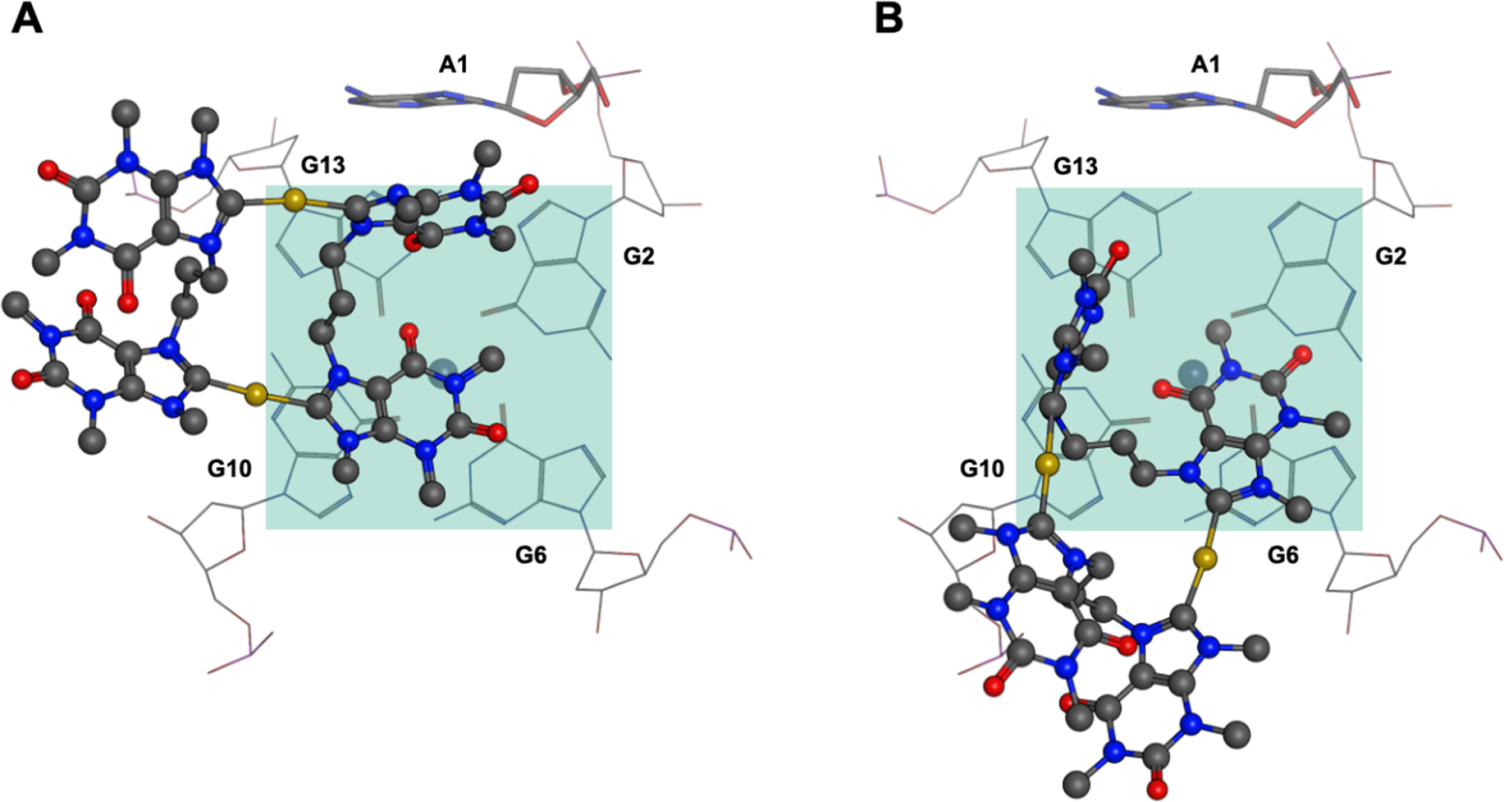
Positions of **AuB3** on the upper G-tetrad of *cKIT1* in the “parallel” orientation with respect to **A1** (A) observed in 21% of the simulation and in the “perpendicular” orientation (B) observed in 47% of the simulations.

Concerning the two binding states (I and II) of **AuB3** with *cKIT1*, a “parallel mode” to base **A1**, with π–π stacking between **AuB3** and bases G6 and G10 (Figure 9A), and a “perpendicular mode” to base **A1** with π–π stacking between **AuB3** and bases G6 and G10 (Figure 9B) can be identified. These two modes are equivalent (equal occurrence of both parallel and perpendicular binding modes) and shape the symmetric appearance of the FES heatmap in Figure 7B.

**Figure 10:**
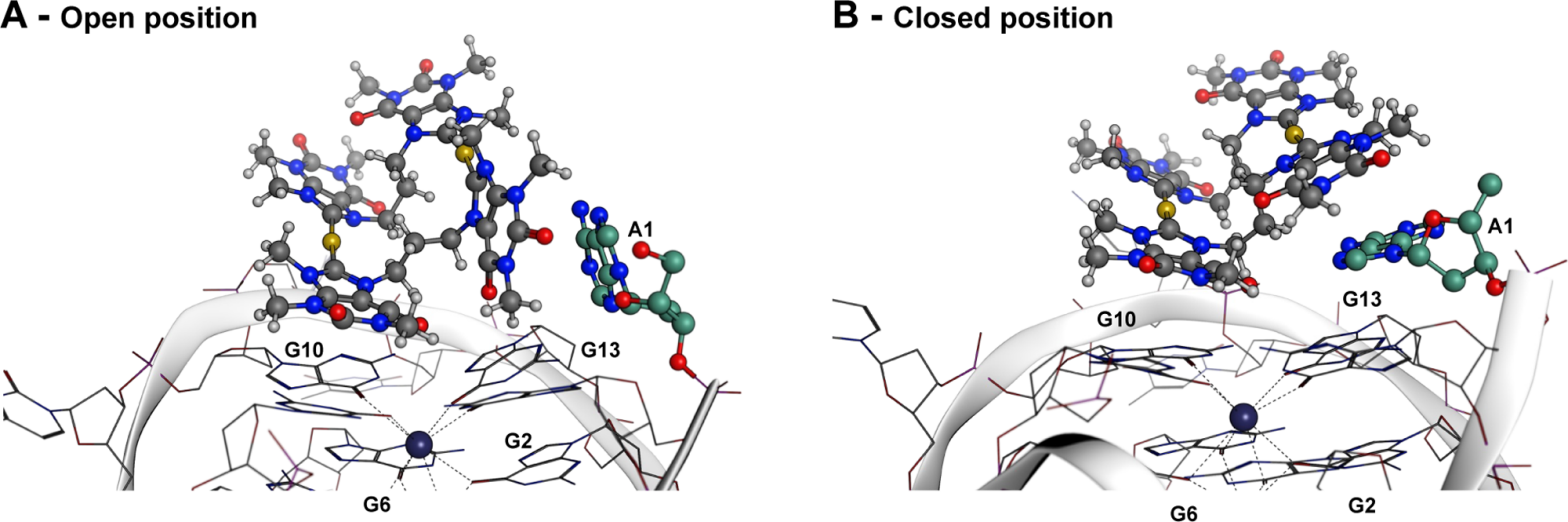
Position of **AuB3** with the upper G-tetrad in both the “open” (A) and “closed” (B) conformations of **A1** (**AuB3** and **A1** shown in ball and stick representation, *cKIT1* shown as a ribbon with bases as a wire. **A1** is colored in green for clarity).

Interestingly, residue **A1** is observed to switch between an “open” and “closed” conformation (Figure 10) in 9 out of 19 trajectories. While the “closed” arrangement hinders the binding of **AuB3** to G2 and G13, the “open” conformation in principle enables the compound to gain access to the nucleobases. However, as mentioned above, only one of the four caffeine units of **AuB3** consistently interacts with the upper tetrad (Figure 8B,C), with a second adjacent TMX forming π–π stacking interactions with **A1**, in either the “open” or “closed” conformation (Figure 9). This lack of further interactions with the upper tetrad residues G2 and G13, even while in the “open” position, is due to the inability of **AuB3** to maintain an overall planar conformation. Accordingly, no significant increase in binding affinity is observed in the Δ*G* values between these two states (closed position = −37.6 ± 4.3 kJ/mol^–1^, open position = −39.3 ± 8.1 kJ/mol^–1^).

On comparing the binding position between the dinuclear Au(I) complex with **AuTMX**_**2**_, the latter interacts with both G6 and G10 guanines located at the G-tetrad top (Figure 8B,C), whereas **AuB3** prefers to interact with only one guanine at a time (Figure 8B,C). Therefore, because **AuB3** cannot outperform **AuTMX**_**2**_ in noncovalent interactions, key to its Δ*G* value is a broader positional variety in molecule-to-G4 interaction poses, which is not observed in **AuTMX**_**2**_ nor in other G4-stabilizing metal complexes.^62^

In conclusion, specific interactions with bases G6 and G10 appear to be key for ligand affinity and *cKIT1* stabilization. The shallow saddle point in the FES (about 5 kJ mol^–1^ above either minimum, Figure 7B) implies a scenario of **AuB3** oscillating between the two energy minima. This dynamic behavior fully compensates for the “imperfect” match between the semi-rigid, sterically demanding **AuB3** molecule and the G-tetrad motif (as illustrated above), which results into a sizable free energy. We, therefore, speculate that FRET DNA melting experiments detect an average value resulting from **AuB3** dynamical (oscillating) and statistical (parallel and perpendicular) orientations. We dub this “shaky lid” behavior of **AuB3** on G-tetrad “dynamical docking”.

### Antiproliferative Activity

The gold compounds were further tested for their antiproliferative activity in three human cancer cell lines, namely, A2780 ovarian cancer, MDA-MB-231, and MCF-7 breast cancer cells lines. The selected breast cancer cells differ in the expression of estrogen (ER)/progesterone (PR) hormone receptors and human epidermal growth factor receptor type 2. While MDA-MB-231 cells are considered triple negative, because they lack all three receptor types, MCF-7 cells are hormone-dependent (ER+/PR+). Moreover, the compounds were also tested against the nontumorigenic VERO E6 (healthy kidney) cell line. The obtained EC_50_ values are reported in Table 2 in comparison to the respective ligands and to the benchmark cisplatin and auranofin.^13^ All ligands were inactive up to a concentration of 100 μM, which highlights the metal contribution in terms of activity enhancement. All the Au(I) complexes were inactive or poorly active in MCF-7 and VERO E6 cells but showed EC_50_ values ranging from ca. 0.44 to ca. 55 μM against A2780 and MDA-MB-231 cells. While in depth investigation of the modes of action should be further addressed in future studies, preliminary structure–activity relationships could be derived: (a) the xanthine-derived Au(I) complexes are moderately active in all cell lines, with EC_50_ values in line with those previously reported for **AuTMX**_**2**_,^28^ (b) ethyl linkers seem beneficial in both series, resulting in the highest activities and following the activity trend: ethylene > propylene > methylene. The only gold complex that outperformed the reference metallodrugs was **AuC2** in A2780 ovarian cancer cells (EC_50_ ca. 0.44 μM, selectivity index >200) and additionally provided the highest selectivity indexes for each cancer cell line compared to both references auranofin and cisplatin.

**Table 2:** EC_50_ Values (μM, *n* ≥ 3) and Selectivity Indexes Calculated as EC_50_(VERO E6)/EC_50_(Cancer Cell Line) of the Au(I) NHC Complexes in Human Cancer and Nontumorigenic Cells^a^

	A2780 (ovarian)	MDA-MB-231 (breast)	MCF-7 (breast)	Vero E6 (noncancerogenic kidney)
cisplatin	0.80 ± 0.08 (15.30)	11.7 ± 1.5 (1.0)	27.9 ± 2.3 (0.4)	12.2 ± 1.2
auranofin	0.27 ± 0.07 (20.44)	3.1 ± 0.1 (1.8)	3.0 ± 0.4 (1.8)	5.5 ± 0.3
**AuTMX**_**2**_	16.2 ± 2.1^b^	n.d.	n.d.	>100
H_2_**B2**^PF6^	>100	>100	>100	>100
H_2_**B3**^PF6^	>100	>100	>100	>100
H_2_**C1**^PF6^	>100	>100	>100	>100
H_2_**C2**^PF6^	>100	>100	>100	>100
H_2_**C3**^PF6^	>100	>100	>100	>100
**AuB2**	12.0 ± 0.9 (>8.3)	52.3 ± 1.5 (>1.9)	>100	>100
**AuB3**	23.7 ± 1.0 (>4.2)	53.7 ± 0.1 (>1.9)	>100	>100
**AuC1**	55.4 ± 2.4 (>1.8)	52.8 ± 0.7 (>1.9)	>100	>100
**AuC2**	0.44 ± 0.13 (>227.27)	28.1 ± 2.0 (>3.5)	52.9 ± 1.4 (>1.9)	>100
**AuC3**	11.18 ± 1.22 (>8.94)	13.1 ± 0.2 (>7.6)	>100	>100

a Cells were incubated with the respective compounds for 72 h.

b Taken from ref (28).

## Conclusions

In conclusion, in order to design potent and selective G-quadruplex stabilizers, two cyclic dinuclear Au(I) bis-NHC complexes **AuB2–3,** in which two xanthine-derived NHC ligands are coupled by an aliphatic bridge, were synthesized and fully characterized, together with three benzimidazole-derived analogues **AuC1–3**. Most of the compounds have shown high stability in solution and in the presence of cysteine nucleophiles and could be studied for their G4s stabilization properties by FRET DNA melting, showing different selectivity patterns depending on the type of the G4 structure. Interestingly, all the Au(I) complexes can stabilize the selected panel of G4s, with their Δ*T*_m_ increasing with the length of the linker chain. While the benzimidazole derivatives show higher G4 stabilization activity with respect to the benchmark mononuclear **AuTMX**_**2**_, they are less selective and can also bind to duplex DNA. Instead, the xanthine-based complexes show moderate selectivity for G4s versus duplex DNA, which is relevant for targeted pharmacological applications. The propyl-bridged complex **AuB3** results as the best performer, showing a comparable stabilizing capability toward hTelo and *cKIT1* with respect to **AuTMX**_**2**_ but significantly higher Δ*T*_m_ with the promoter *cKIT2*, *BCL2*, and *hTERT*. Furthermore, **AuB3** also stabilizes parallel G4s (such as *cKIT1* and *cKIT2*) to a greater extent than the hybrid-mixed hTelo or *BCL2* sequences, highlighting a potential preference toward the parallel G4-conformation. Additionally, the appearance of weak ICD bands in the G4s′ CD spectra in the presence of either **AuB3** (and also **AuC3**) suggests the complexes’ top stacking onto the G4 tetrads.

In order to shed light into the mechanisms of binding of the cyclic dinuclear Au(I) bis-NHC complexes with G4s at a molecular/atomic level, metaD simulations were applied to study the adduct of the most potent xanthine derivative **AuB3** with *cKIT1*. The compound interacts with the top tetrad of the G4 monomer establishing noncovalent interactions with some of the nucleobases and oscillating between two energy minima. As expected from the similar experimental Gibbs free energies of G4 stabilization, the cyclic dinuclear **AuB3** does not gain any improved stabilizing interaction with *cKIT1* with respect to **AuTMX**_**2**_. This result is well justified by the metaD analysis, whereby the compound is shown to escape from a rigid planar structure to achieve a “dynamical docking” on the upper tetrad, enabling only “half” of the molecule to actually interact with some of the guanines in *cKIT1*. The experimental validation by FRET DNA melting of the binding energy calculated by metaD was also achieved for the **AuB3** adducts with *cKIT1*. Preliminary antiproliferative activity data showed only moderate activity of the xanthine-based Au(I) complexes in cancer cells, while more intriguing cytotoxicity profiles were observed for the benzimidazole derivatives. Nevertheless, all the cyclic dinuclear compounds were nontoxic against nontumorigenic cells.

Overall, our study has evidenced that cyclic dinuclear Au(I) bis-NHC complexes based on xanthine scaffolds may provide some advantages over mononuclear complexes, particularly in the adaptability toward binding to different G4 structures likely due to the flexibility conferred by the alkyl linkers. However, care should be paid to obtain the optimal balance between metallodrug rigidity and conformational adaptability to achieve maximal G4 stabilization. Finally, it is also worth mentioning that metal bis-NHCs complexes derived from purines, including of Pt(II), Pd(II), and Ag(I), have attracted attention for different therapeutic applications in recent years,^63−65^ and our results point toward a possible common noncovalent binding modes with pharmacological targets of these families of organometallics.

## Experimental Section

### General

Reactions involving dry solvents were performed under a nitrogen atmosphere and in flame-dried glassware. Syntheses involving silver or gold were carried out in the dark. The remaining manipulations, unless otherwise stated, were conducted under standard conditions. Nitrobenzene was dried by distillation and discarding of the first 5% chloroform by passing it over a plug of basic alumina (75 g for 100 mL of solvent). All other solvents as well as starting materials were commercially obtained and used without further purification. Column chromatography was performed on silica gel 60A (particle size 40–63 μm). Merck Millipore silica gel 60 F-254 plates were employed for thin-layer chromatography (TLC), which were analyzed in UV light (λ = 254 nm) or stained with an alkaline potassium permanganate solution. NMR spectra were recorded on a Bruker AVANCE DPX 400 at ambient temperature (400.13 MHz for ^1^H NMR, 100.53 MHz for ^13^C{^1^H} NMR, and 161.97 MHz for ^31^P NMR). Chemical shifts δ are reported in parts per million (ppm) with respect to residual ^1^H and ^13^C signals of the deuterated solvents as the internal standard, whereas coupling constants *J* are denoted in Hertz (Hz). ^13^C{^1^H} NMR spectra were baseline-corrected and occasionally exponential apodization functions were used to improve resolution. The following abbreviations as well as combinations thereof are employed for NMR signal multiplicities: s = singlet, d = doublet, t = triplet, p = quintet, h = heptet, and b = broad. Elemental analyses (EAs) for C, H, N, and S were obtained from a HEKAtech Euro EA by the microanalytical laboratory at the Technical University of Munich. Measurements that were below the limit of detection are denoted with *bdl*.

HR-ESI-MS was conducted on a Thermo Fisher Exactive Plus Orbitrap mass spectrometer, which was equipped with an ESI source by the same company. Samples were prepared as 1 mg mL^–1^ solutions in acetonitrile, syringe filtered to 0.45 μm, and directly injected. With an ionization voltage of 2 kV, ions were detected in the positive mode. Peaks were then fit with Gaussian functions and compared to isotope patterns calculated in enviPat Web 2.4.^66^ Mass-charge ratios *m*/*z* are given in g mol^–1^ e^–1^. Chloro(tetrahydrothiophene)gold(I) [Au(THT)Cl],^67^ the benchmark Au(I) compound **AuTMX**_**2**_,^19^ ligands, 1,1′-trimethylenebis(benzimidazole)^68^ and 1,1′-methylenebis(3,3′-dimethylbenzimidazolium) dibromide (**H**_**2**_**C1**^Br^),^42^ were synthesized accordingly to previously published procedures. The syntheses of 7,7′-methylenebis(theophylline) (**A1**), 7,7′-ethylenebis(theophylline) (**A2**), and 7,7′-trimethylenebis(theophylline) (**A3**) were performed according to previously reported procedures.^69^ The compounds’ purity was assessed to be >95%.

### Synthesis of NHC Ligands

#### General Procedure for the Synthesis of 7,7′-(α,ω-Alkanediyl)-bis(theophylline)

Adapted from a general procedure by Itahara and Imamura.^69^ Theophylline (2.80 g, 15.5 mmol, 2.00 equiv) was dissolved in 100 mL dry DMF under a N_2_ atmosphere to give a clear yellow solution and NaH (625 mg of a 60% dispersion in mineral oil, 15.6 mmol, 2.01 equiv) was added, rinsing the powder funnel with another 25 mL dry DMF. After 30 min, 1.00 equiv Br(CH_2_)_n_Br (7.77 mmol) was added and the solution was stirred for 22 h at room temperature. A further heating up to 70 °C for another 5 h was applied prior to pouring the suspension into ∼200 mL of distilled water, stirring it for 5 min, and leaving it in the fridge to crystallize. The precipitate was vacuum filtered and washed with 50–80 mL of ice-cold distilled water. It was then dried in high vacuum and subsequently washed with 60–90 mL hexanes or ^*n*^pentane to remove residual mineral oil originating from the NaH dispersion.

##### Synthesis of 1-Methylbenzimidazole

The title compound was synthesized by modification of literature procedures.^70,71^ 10.0 g benzimidazole (84.7 mmol, 1.00 equiv) were suspended in 40 mL 50% aq NaOH at 0 °C. Upon warming to 30 °C, the solid dissolved almost completely. Over a period of 20 min, 13.7 g MeI (96.4 mmol, 1.14 equiv) were added dropwise to the well-stirred suspension, resulting in a biphasic system. The reaction was completed after stirring at 40 °C for another 70 min as confirmed by TLC. The aqueous phase was extracted with DCM (4 × 30 mL) and the combined organic layers were washed with distilled water (1 × 15 mL). After drying over anhydrous MgSO_4_, filtration, and evaporation of volatiles in vacuo, 11.15 g of a brown crude oil were obtained. Purification by column chromatography (275 g silica, gradient 3 → 7% MeOH in DCM) gave 9.41 g 1-methylbenzimidazole (71.2 mmol, 84%) as yellow oil that crystallized to a beige solid. NMR was in accordance with the literature.

##### Synthesis of 7,7′-Methylenebis(9,9′-dimethyltheophyllinium) Bis(methyl sulfate) (H_2_**B1**^MeSO_4_^)

400 mg **A1** (1.07 mmol, 1.00 equiv) were dissolved in 40 mL dry PhNO_2_ at 90 °C to give a greenish-yellow, clear solution. Me_2_SO_4_ (3 mL, ∼4 g, ∼30 equiv) was added dropwise to the stirred solution of **A1**, resulting in immediate clouding of the reaction mixture. The reaction monitoring by NMR was employed: 0.3 mL were worked up by pouring on 1.7 mL Et_2_O, centrifuging, decanting, and washing with 1 mL Et_2_O twice. After a total of 6 days, conversion was satisfactory. Approximately 40% of the initial reaction mixture (the rest was consumed by failed parallel attempts) were worked up by pouring into 50 mL Et_2_O, filtration, and washing with 3 × 10 mL Et_2_O. With the described procedure, 244 mg (391 μmol) of the crude title compound were obtained, corresponding to a yield of ∼90% with respect to the partial amount that was worked up. The brown powder, which is highly hygroscopic and extremely sensitive toward alkaline conditions was stored at −30 °C under a N_2_ atmosphere and used without further purification. In DMSO, H_2_**B1**^MeSO_4_^ dissociates in less than 2 days. ^1^H NMR (DMSO-*d*_6_): δ 3.28 (s, 6 H, C*H*_3_), 3.34 (s, 6 H, C*H*_3_), 3.74 (s, 6 H, C*H*_3_), 4.26 (s, 6 H, C*H*_3_), 7.04 (s, 2 H, C*H*_2_), 9.66 (s, 2 H, NC*H*N).

##### Synthesis of 7,7′-Ethylenebis(9,9′-dimethyltheophyllinium) Ditosylate (H_2_**B2**^OTs^)

**A2** (250 mg, 647 μmol, 1.00 equiv) was united with MeOTs (2.50 g, 13.4 mmol, 20.8 equiv). On heating, the white suspension resolved to a yellow solution around 140 °C and was further warmed to 160 °C, at which temperature the reaction mixture was stirred for 2 h. Once cooled down, the resulting dark amber oil was poured into 40 mL Et_2_O to form a white to brown, sticky precipitate. The supernatant was decanted off and the solid was recrystallized from ^*i*^PrOH/EtOH (∼1/1, 20 mL). The white crystals were filtered off and washed with 2 × 4 mL ice-cold ^*i*^PrOH. Drying in high vacuum afforded the title compound H_2_**B2**^OTs^ as 405 mg (534 μmol, 83%) of a white powder. ^1^H NMR (DMSO-*d*_6_): δ 2.28 (s, 6 H, Me_tosylate_), 3.27 (s, 6 H, C*H*_3_), 3.70 (s, 6 H, C*H*_3_), 4.09 (s, 6 H, C*H*_3_), 4.97 (s, 4 H, C*H*_2_), 7.10 (d, ^3^*J*_HH_ = 7.7, 4 H, C*H*_ar, tosylate_), 7.42 (d, ^3^*J*_HH_ = 7.8, 4 H, C*H*_ar, tosylate_), 9.26 (s, 2 H, NC*H*N).

##### Synthesis of 7,7′-Trimethylenebis(9,9′-dimethyltheophyllinium) Ditosylate (H_2_**B3**^OTs^)

**A3** (300 mg, 749 μmol, 1.00 equiv) and MeOTs (3.00 g, 16.1 mmol, 21.5 equiv) were united and heated to 160 °C, at which temperature they formed a clear yellow solution. After 2 h, the resulting dark amber solution was cooled to room temperature and poured into 50 mL Et_2_O. The supernatant was decanted off, leaving a grayish-orangey precipitate, which was then recrystallized from ^*i*^PrOH/EtOH (∼1/1, 25 mL, hot filtration). Colorless needles were filtered off, washed with 4 mL cold ^*i*^PrOH as well as 5 mL Et_2_O, and dried in vacuo to give the title compound in 80% yield (476 mg, 597 μmol). ^1^H NMR (DMSO-*d*_6_): δ 2.28 (s, 6 H, Me_tosylate_), 2.45 (p, ^3^*J*_HH_ = 6.8, 2 H, CH_2_C*H*_2_CH_2_), 3.25 (s, 6 H, C*H*_3_), 3.72 (s, 6 H, C*H*_3_), 4.13 (s, 6 H, C*H*_3_), 4.53 (t, ^3^*J*_HH_ = 6.8, 4 H, NC*H*_2_), 7.10 (d, ^3^*J*_HH_ = 8.0, C*H*_tosylate_), 7.43 (d, ^3^*J*_HH_ = 8.1, C*H*_tosylate_), 9.43 (s, 2 H, NC*H*N).

##### Synthesis of 1,1′-Ethylenebis(3,3′-dimethylbenzimidazolium) Dibromide (H_2_**C2**^Br^)

1-Methylbenzimidazole (1.13 g, 8.53 mmol, 2.46 equiv) was dissolved in 10 mL dry MeCN. 1,2-Dibromoethane (650 mg, 3.46 mmol, 1.00 equiv) was added to give a clear, yellowish solution. The mixture was refluxed for 3 days. The solvent was removed from the resulting white suspension in vacuo and the yellowish solid was washed with 25 mL THF in three portions. Recrystallization from 150 mL EtOH gave the title compound H_2_**C2**^Br^ as pure white needles (1.23 g, 2.72 mmol, 79%). NMR was in accordance with the literature.^42 1^H NMR (DMSO-*d*_6_): δ 4.05 (s, 6 H, C*H*_3_), 5.14 (s, 4 H, C*H*_2_), 7.62 (ddd, *J*_HH_ = 8.3|7.3|1.2, 2 H, C*H*_ar_), 7.69 (ddd, *J*_HH_ = 8.3|7.3|1.1, 2 H, C*H*_ar_), 7.92 (dt, *J*_HH_ = 8.2|1.0, 2 H, C*H*_ar_), 8.03 (dt, *J*_HH_ = 8.4|1.0, 2 H, C*H*_ar_), 9.83 (s, 2 H, NC*H*N). ^13^C{^1^H} NMR (DMSO-*d*_6_): δ 33.41 (*C*H_3_), 45.53 (*C*H_2_), 112.99 (*C*H_ar_), 113.72 (*C*H_ar_), 126.65 (*C*H_ar_), 126.72 (*C*H_ar_), 130.77 (*C*_ar_), 131.68 (*C*_ar_), 143.40 (N*C*HN).

##### Synthesis of 1,1′-Trimethylenebis(3,3′-dimethylbenzimidazolium) Bis(tetrafluoroborate) (H_2_**C3**^BF4^)

500 mg 1,1′-trimethylene-bis(benzimidazole) (∼90% purity, prior to recryst., 1.63 mmol, 1.00 equiv) were dissolved in 20 mL dry MeCN under slight warming. 600 mg Me_3_OBF_4_ (4.06 mmol, 2.49 equiv) were added, leading to immediate clouding of the solution. After 4 h of vigorous stirring, another 25 mL dry MeCN were added to obtain a clear solution and additional 60 mg (0.41 mmol, 0.25 equiv) Me_3_OBF_4_ were added. After stirring overnight, the reaction was worked up by the removal of the solvent at reduced pressure. 1.04 g yellowish crude product were washed with 150 mL boiling MeOH. The wash fraction was reduced to 105 mL, out of which further product was obtained by crystallization upon cooling. In total, 563 mg (1.17 mmol, 72%) H_2_**C3**^BF4^ were obtained as a white powder. ^1^H NMR (DMSO-*d*_6_): δ 2.58 (p, ^3^*J*_HH_ = 7.2, 2 H, CH_2_C*H*_2_CH_2_), 4.07 (s, 6 H, C*H*_3_), 4.65 (t, ^3^*J*_HH_ = 7.3, 4 H, NC*H*_2_), 7.67–7.76 (m, 4 H, C*H*_ar_), 7.99–8.09 (m, 4 H, C*H*_ar_), 9.66 (s, 2 H, NC*H*N). ^13^C{^1^H} NMR (DMSO-*d*_6_): δ 28.04 (CH_2_*C*H_2_CH_2_), 33.26 (*C*H_3_), 43.78 (N*C*H_2_), 113.36 (*C*H_ar_), 113.63 (*C*H_ar_), 126.54 (*C*H_ar_), 126.60 (*C*H_ar_), 130.84 (*C*_ar_), 131.84 (*C*_ar_), 142.84 (N*C*HN). Assigned by HSQC NMR. Calcd for C_19_H_22_B_2_F_8_N_4_ (H_2_**C3**^BF4^) [%]: C, 47.54; H, 4.62; N, 11.67. Found [%]: C, 47.36; H, 4.55; N, 11.65. Single crystals suitable for diffraction were obtained by slow diffusion of Et_2_O into a solution of H_2_**C3**^BF4^ in MeCN.

#### General Procedure for Ion Exchange to PF_6_

Benzimidazole ligands: the compound is dissolved in a minimal amount of distilled water (20–75 mM) by careful warming to 60–80 °C under stirring. Five to 6 M equiv. NH_4_PF_6_ are dissolved separately in distilled water to give an approximately 1 M solution and heated to the same temperature. The first solution was slowly poured into the second one under vigorous stirring, resulting in the immediate formation of a white suspension. Stirring is continued on cooling to room temperature. The precipitate was collected by filtration and washed thoroughly with cold distilled water. Following drying in high vacuum, if elemental analysis purity was not reached, the product is further resuspended and treated as described above.

Xanthine ligands: the crude product was dissolved in a minimal amount of distilled water at room temperature (∼0.2 M) and cotton-filtered into a vigorously stirred, approximately 2 M aq solution of 10 equiv NH_4_PF_6_. The resulting white suspension was stirred for another 15 min. Sometimes, gel-like lumps form initially but resolve to a suspension on continued stirring. The white precipitate was collected by filtration and washed thoroughly with ice-cold water prior to drying in high vacuum.

##### Synthesis of 7,7′-Methylenebis(9,9′-dimethyltheophyllinium) Bis(hexafluorophosphate) (H_2_**B1**^PF6^)

H_2_**B1**^MeSO_4_^ (162 mg, 269 μmol, 1.00 equiv) was dissolved in 8 mL MeOH and poured into a solution of NH_4_PF_6_ (500 mg, 3.07 mmol, 11.8 equiv) in 4 mL distilled water. Soon after, silver needles started to grow in the solution. Crystallization was aided by cooling in the freezer. The precipitate was collected by filtration, washed (3× 6 mL distilled water), and dried in high vacuum. H_2_**B1**^PF6^ was hereby obtained as an off-white powder in 49% yield (88 mg, 0.13 mmol). The compound was further purified by recrystallization from a mixture of acetone and EtOAc to give a pure white powder. ^1^H NMR (CD_3_CN): δ 3.36 (s, 6 H, C*H*_3_), 3.73 (s, 6 H, C*H*_3_), 4.14 (s, 6 H, C*H*_3_), 6.95 (s, 2 H, C*H*_2_), 9.05 (s, 2 H, NC*H*N). ^13^C{^1^H} NMR (CD_3_CN): δ 29.47 (*C*H_3_), 32.41 (*C*H_3_), 38.97 (*C*H_3_), 57.38 (*C*H_2_), 108.27, 140.95, 142.13 (N*C*HN), 151.09, 154.95. Assigned by HSQC NMR. ^31^P NMR (CD_3_CN): δ −144.9 (h, ^1^*J*_PF_ = 706.9). Calcd for C_17_H_24_F_12_N_8_O_5_P_2_ (H_2_**B1**^PF6^·H_2_O) [%]: C, 28.74; H, 3.41; N, 15.77. Found [%]: C, 28.57; H, 3.08; N, 15.31.

##### Synthesis of 7,7′-Ethylenebis(9,9′-dimethyltheophyllinium) Bis(hexafluorophosphate) (H_2_**B2**^PF6^)

Anion exchange of H_2_**B2**^OTs^ (500 mg, 659 μmol) according to the general procedure afforded H_2_**B2**^PF6^ as a white powder in 91% yield. ^1^H NMR (CD_3_CN): δ 3.33 (s, 6 H, C*H*_3_), 3.73 (s, 6 H, C*H*_3_), 4.05 (s, 6 H, C*H*_3_), 4.98 (s, 4 H, C*H*_2_), 8.35 (s, 2 H, NC*H*N). ^13^C{^1^H} NMR (CD_3_CN): δ 29.31 (*C*H_3_), 32.32 (*C*H_3_), 38.44 (*C*H_3_), 49.30 (*C*H_2_), 108.91 (*C*_sp_2), 139.99 (N*C*HN), 141.09 (*C*_sp_2), 151.31 (*C*_sp_2), 154.63 (*C*_sp_2). Assigned by analogy to H_2_**B2**^OTs^. ^31^P NMR (CD_3_CN): δ −144.7 (h, ^1^*J*_PF_ = 707.3). Calcd for C_18_H_24_F_12_N_8_O_4_P_2_ (H_2_**B2**^PF6^) [%]: C, 30.61; H, 3.42; N, 15.86; S, 0.00. Found [%]: C, 30.45; H, 3.50; N, 15.59, S *bdl*.

##### Synthesis of 7,7′-Trimethylenebis(9,9′-dimethyltheophyllinium) Bis(hexafluorophosphate) (H_2_**B3**^PF6^)

Anion exchange of H_2_**B3**^OTs^ (383 mg, 496 μmol) according to the general procedure afforded H_2_**B3**^PF6^ as a white powder in 77% yield. ^1^H NMR (CD_3_CN): δ 2.48 (p, ^3^*J*_HH_ = 7.1, 2 H, CH_2_C*H*_2_CH_2_), 3.31 (s, 6 H, C*H*_3_), 3.74 (s, 6 H, C*H*_3_), 4.09 (s, 6 H, C*H*_3_), 4.53 (t, ^3^*J*_HH_ = 7.1, 4 H, NC*H*_2_), 8.52 (s, 2 H, NC*H*N). ^13^C{^1^H} NMR (CD_3_CN): δ 29.22 (*C*H_3_), 30.83 (CH_2_*C*H_2_CH_2_), 32.30 (*C*H_3_), 38.24 (*C*H_3_), 46.82 (N*C*H_2_), 108.97 (*C*_sp_2), 139.56 (N*C*HN), 141.04 (*C*_sp_2), 151.39 (*C*_sp_2), 154.55 (*C*_sp_2). Assigned by HSQC NMR. ^31^P NMR (CD_3_CN): δ −144.68 (h, ^1^*J*_PF_ = 705.9). Calcd for C_19_H_28_F_12_N_8_O_5_P_2_ (H_2_**B3**^PF6^·H_2_O) [%]: C, 30.91; H, 3.82; N, 15.18; S, 0.00. Found [%]: C, 30.63; H, 3.50; N, 14.87, S *bdl*.

Synthesis of 1,1′-methylenebis(3,3′-dimethylbenzimidazolium) bis(hexafluorophosphate) (H_2_**C1**^PF6^). 1,1′-ethylenebis(3,3′-dimethylbenzimidazolium) bis(hexafluorophosphate) (H_2_**C2**^PF6^) and 1,1′-trimethylenebis(3,3′-dimethylbenzimidazolium) bis(hexafluorophosphate) (H_2_**C3**^PF6^). Anion exchange was carried from the Br- (for H_2_**C1** and H_2_**C2**) and BF_4_ precursors of the ligands according to the general procedure. ^1^H and ^13^C{^1^H} NMR were in accordance with literature^42^ and purity confirmed by elemental analysis to be >95%.

**H**_**2**_**C1**^PF6^: white powder, 82% yield (534 mg). **H**_**2**_**C2**^PF6^: white powder, 76% yield (780 mg). **H**_**2**_**C3**^PF6^ as white powder in 91% yield (463 mg).

#### Synthesis of Cyclic Dinuclear Au(I) Bis-NHC Complexes

##### Synthesis of [Au_2_(μ-{7,7′-Ethylenebis(9,9′-dimethyltheophyllin-8,8′-ylidene)})_2_](PF_6_)_2_ (**AuB2**)

66.1 mg H_2_**B2**^PF6^ (93.6 μmol) and 1.01 equiv Au(THT)Cl were dissolved in dry MeCN (2 mL ca), and stirred at 55 °C for an hour in the dark, prior to the addition of 5.0 equiv of oven-dried, freshly crushed K_2_CO_3_. After 24 h stirring, the reaction was filtrated over Celite and rinsed with fresh MeCN. The final product was achieved via fractional precipitation, upon addition of a large excess of Et_2_O, and centrifugation followed by further washes with small aliquots of Et_2_O. **AuB2** was afforded as 52 mg (73%) of a white powder. ^1^H NMR (CD_3_CN): δ 3.27 (s, 12 H, C*H*_3_), 3.73 (s, 12 H, C*H*_3_), 4.15 (s, 12 H, C*H*_3_), 5.28 (br s, 8 H, C*H*_2_). ^13^C{^1^H} NMR (CD_3_CN): δ 28.98 (*C*H_3_), 32.47 (*C*H_3_), 40.50 (*C*H_3_), 46.69 (N*C*H_2_), 108.89 (*C*_sp_2), 141.51 (*C*_sp_2), 151.72 (*C*_sp_2), 154.47 (*C*_sp_2), 187.60 (*C*–Au). Assigned by HSQC NMR. ^31^P NMR (CD_3_CN): δ −144.65 (h, ^1^*J*_PF_ = 706.2). Calcd for C_36_H_48_Au_2_F_12_N_16_O_10_P_2_ (**AuB2**·2H_2_O) [%]: C 27.92; H 3.12; N, 14.47. Found [%]: C, 27.88; H, 2.94; N 14.07. *m*/*z*: 611.1417 (611.1424 calcd for [**AuB2**-2 PF_6_]^2+^), 1367.2482 (1367.2496 calcd for [**AuB2**-PF_6_]^+^). Single crystals suitable for diffraction were obtained by slow diffusion of Et_2_O into a solution of **AuB2** in DMF.

##### Synthesis of [Au_2_(μ-{7,7′-Trimethylenebis(9,9′-dimethyltheophyllin-8,8′-ylidene)})_2_](PF_6_)_2_**(AuB3**)

67.4 mg H_2_**B3**^PF6^ (93.6 μmol) and 1.01 equiv Au(THT)Cl are dissolved in dry MeCN (2 mL ca) and stirred at 55 °C for an hour in the dark, prior to the addition of 5.0 equiv of oven-dried, freshly crushed K_2_CO_3_. After 24 h stirring, the reaction was filtered over Celite and rinsed with fresh MeCN. The final product was achieved via fractional precipitation, upon the addition of a large excess of Et_2_O, and centrifugation followed by further washes with small aliquots of Et_2_O. **AuB3** was afforded as 25 mg (37%) of a white powder. ^1^H NMR (CD_3_CN): δ 2.59 (p, ^3^*J*_HH_ = 6.9, 4 H, CH_2_C*H*_2_CH_2_), 3.28 (s, 12 H, C*H*_3_), 3.78 (s, 12 H, C*H*_3_), 4.19 (s, 12 H, C*H*_3_), 4.61 (t, ^3^*J*_HH_ = 6.9, 8 H, N*C*H_2_). ^13^C{^1^H} NMR (CD_3_CN): δ 29.01 (*C*H_3_), 31.23 (CH_2_*C*H_2_CH_2_), 32.58 (*C*H_3_), 39.98 (*C*H_3_), 49.48 (N*C*H_2_), 109.89 (*C*_sp_2), 142.22 (*C*_sp_2), 151.89 (*C*_sp_2), 154.37 (*C*_sp_2), 187.55 (*C*-Au). ^31^P NMR (CD_3_CN): δ −144.66 (h, ^1^*J*_PF_ = 707.7). Calcd for C_38_H_50_Au_2_F_12_N_16_O_9_P_2_ (**AuB3**·H_2_O) [%]: C, 29.28; H, 3.23; N, 14.38. Found [%]: C, 29.44; H, 3.23; N, 13.89. *m*/*z*: 625.1572 (625.1581 calcd for [**AuB3**-2 PF_6_]^2+^), 1395.2791 (1395.2809 calcd for [**AuB3**-PF_6_]^+^). Single crystals suitable for diffraction were obtained by slow diffusion of Et_2_O into a solution of **AuB3** in DMF.

##### Synthesis of [Au_2_(μ-{1,1′-Methylenebis(3,3′-dimethylbenzimidazol-2,2′-ylidene)})_2_](PF_6_)_2_ (**AuC1**)

100 mg H_2_**C1**^PF6^ (176 μmol) and 1.01 equiv Au(THT)Cl are dissolved in dry MeCN (3 mL ca) and stirred at 60 °C for an hour in the dark, prior to the addition of 5.0 equiv of oven-dried, freshly crushed K_2_CO_3_. After a day of stirring, the reaction is filtered over Celite and rinsed with fresh MeCN. The final product was achieved via fractional precipitation, upon the addition of a large excess of dry PhMe and ^*n*^pentane. The precipitate was collected via centrifugation and further washed with small aliquots of ^*n*^pentane to afford **AuC1** as 100 mg (92%) of an off-white powder. ^1^H NMR (CD_3_CN): δ 4.06 (s, 12 H, C*H*_3_), 7.00 (d, ^1^*J*_HH_ = 14.8, 1 H, NC*H*HN), 7.31 (d, ^1^*J*_HH_ = 14.7, 1 H, NCH*H*N), 7.58–7.65 (m, 8 H, C*H*_ar_), 7.72–7.78 (m, 4 H, C*H*_ar_), 7.86–7.92 (m, 4 H, C*H*_ar_). ^13^C{^1^H} NMR (CD_3_CN): δ 36.90 (*C*H_3_), 59.62 (*C*H_2_), 112.65 (*C*H_ar_), 113.92 (*C*H_ar_), 126.74 (*C*H_ar_), 126.97 (*C*H_ar_), 133.53 (*C*_ar_), 135.09 (*C*_ar_), 191.86 (*C*-Au). Assigned by HSQC NMR. ^31^P NMR (CD_3_CN): δ −144.63 (h, ^1^*J*_PF_ = 706.4). Calcd for C_34_H_38_Au_2_F_12_N_8_O_3_P_2_ (**AuC1**·3H_2_O) [%]: C, 31.64; H, 2.97; N, 8.68. Found [%]: C, 31.09; H, 2.39; N, 8.46. *m*/*z*: 473.1029 (473.1035 calcd for [**AuC1**-2 PF_6_]^2+^), 1091.1698 (1091.1718 calcd for [**AuC1**-PF_6_]^+^). Single crystals suitable for diffraction were obtained by slow diffusion of Et_2_O into a solution of **AuC1** in MeCN.

##### Synthesis of [Au_2_(μ-{1,1′-Ethylenebis(3,3′-dimethylbenzimidazol-2,2′-ylidene)})_2_](PF_6_)_2_ (**AuC2**)

75.0 mg H_2_**C2**^PF6^ (129 μmol, 1.00 equiv) and 298 mg Ag_2_O (1.29 mmol, 9.98 equiv) were suspended in 7.0 mL dry acetonitrile. Over the weekend, the reaction mixture was stirred at room temperature in the dark. Excess Ag_2_O was removed by Celite filtration. Au(THT)Cl (41.8 mg, 130 μmol, 1.01 equiv) was then added, which immediately resulted in the formation of a fine, white precipitate. After stirring overnight, the precipitate of AgCl was removed by centrifugation and decanting. The product was isolated by fractional precipitation with dry PhMe and ^*n*^pentane (impurities precipitate after the product), collection by centrifugation, washing with small aliquots of ^*n*^pentane, and drying in high vacuum. **AuC2** was obtained as 28 mg (22 μmol, 34%) of a gray powder. ^1^H NMR (CD_3_CN): δ 3.95 (s, 12 H, C*H*_3_), 5.29 (s, 8 H, C*H*_2_), 7.33 (ddd, *J*_HH_ = 8.3|7.1|1.1, 4 H, C*H*_ar_), 7.40–7.47 (m, 8 H, C*H*_ar_), 7.54 (dt, *J*_HH_ = 8.3|0.9, 4 H, C*H*_ar_). ^13^C{^1^H} NMR (CD_3_CN): δ 36.24 (*C*H_3_), 47.68 (*C*H_2_), 112.18 (*C*H_ar_), 113.15 (*C*H_ar_), 125.90 (*C*H_ar_), 125.99 (*C*H_ar_), 133.99 (*C*_ar_), 134.77 (*C*_ar_), 191.37 (*C*-Au). ^31^P NMR (CD_3_CN): δ −144.60 (h, ^1^*J*_PF_ = 707.0). Calcd for C_36_H_38_Au_2_F_12_N_8_OP_2_ (**AuC2**·H_2_O) [%]: C, 33.71; H, 2.99; N, 8.74. Found [%]: C, 33.35; H, 2.57; N, 8.94. *m*/*z*: 487.1186 (487.1192 calcd for [**AuC2**-2 PF_6_]^2+^), 1119.2017 (1119.2031 calcd for [**AuC2**-PF_6_]^+^). Single crystals suitable for diffraction were obtained by slow evaporation of a solution of **AuC2** in MeCN.

##### Synthesis of [Au_2_(μ-{1,1′-Trimethylenebis(3,3′-dimethylbenzimidazol-2,2′-ylidene)})_2_](PF_6_)_2_ (**AuC3**)

59.9 mg H_2_**C3**^PF6^ (100 μmol), and 2.01 equiv Au(THT)Cl were dissolved in dry MeCN (3 mL ca) and stirred at 60 °C for an hour in the dark, prior to the addition of 5.0 equiv of oven-dried, freshly crushed K_2_CO_3_. After a day of stirring, the reaction was filtered over Celite and rinsed with fresh MeCN. The final product was achieved via fractional precipitation via the addition of a large excess of dry PhMe and ^*n*^pentane. The precipitate was collected by centrifugation and further washed with small aliquots of ^*n*^pentane to afford **AuC3** as 45 mg (69%) of a white powder. ^1^H and ^13^C{^1^H} NMR were in accordance with literature^36^ and the purity confirmed by elemental analysis to be >95%.

### X-ray Crystallography

Data were collected on a Bruker D8 Venture single-crystal X-ray diffractometer equipped with a CMOS detector (Photon-100), a TXS rotating anode with Mo Kα radiation (λ = 0.71073 Å), and a Helios optic (compounds **H**_**2**_**C3**, **AuB2**) or a CPAD detector (Photon-II), an IMS microfocus source with Mo Kα radiation and a Helios optic (compounds **AuB3**, **AuC1**, **AuC2**) using the APEX3 software package.^72^ The measurements were performed on single crystals coated with perfluorinated ether. The crystals were fixed on top of a Kapton micro sampler and frozen under a stream of cold nitrogen. A matrix scan was used to determine the initial lattice parameters. Reflections were corrected for Lorentz and polarization effects, scan speed, and background using SAINT.^73^ Absorption correction, including odd and even ordered spherical harmonics, was performed using SADABS.^73^ The space group assignment was based upon systematic absences, E statistics, and successful refinement of the structures. The structures were solved using SHELXT with the aid of successive difference Fourier maps and were refined against all data using SHELXL in conjunction with SHELXLE.^74−76^ Hydrogen atoms (except on heteroatoms) were calculated in ideal positions as follows: methyl hydrogen atoms were refined as part of rigid rotating groups, with a C–H distance of 0.98 Å and *U*_iso_(H) = 1.5*U*_eq_(C). Non-methyl H atoms were placed in calculated positions and refined using a riding model with methylene, aromatic, and other C–H distances of 0.99, 0.95, and 1.00 Å, respectively, and *U*_iso_(H) = 1.2*U*_eq_(C). Non-hydrogen atoms were refined with anisotropic displacement parameters. Full-matrix least-squares refinements were carried out by minimizing Σ*w*(*F*_o_^2^ – *F*_c_^2^)2 with the SHELXL weighting scheme.^74^ Neutral atom scattering factors for all atoms and anomalous dispersion corrections for the non-hydrogen atoms were taken from International Tables for Crystallography.^77^ Images of the crystal structure were generated with mercury.^78^ Deposition numbers 2202916–2202920 contain the supplementary crystallographic data for this paper. These data are provided free of charge by the joint Cambridge Crystallographic Data Centre and Fachinformationszentrum Karlsruhe Access Structures service www.ccdc.cam.ac.uk/structures.

### NMR Stability Studies

Dinuclear gold complexes **AuB2–3** and **AuC1–3** were tested for their stability in a 1/4 mixture of D_2_O and DMSO-*d*_6_ over time and in the presence of the model thiol NAC, in the same solvent mixture. Equal amounts of every gold complex (∼3 mg) were dissolved in 0.5 mL solvent mixture each and ^1^H NMR spectra were recorded over time, after mixing, and at 1, 3, 8, and 24 h after dissolution. Similarly, ^1^H NMR spectra of the Au(I) compounds in the presence of an equimolar NAC amount were recorded at the same time intervals.

### FRET DNA Melting Assay

FRET experiments were run on an Applied Biosystems QuantumStudio 5 Real-Time PCR thermocycler (Thermo Fisher Scientific, Waltham, USA) equipped with a FAM filter (λex = 492 nm; λem = 516 nm). The thermocycler was set to perform a stepwise increase of 0.3 °C every 30 s, from 25 to 95 °C, and measurements were acquired after each step. All the oligonucleotides were purchased from Sigma-Aldrich (Germany) in HPLC purity grade. The FRET probes used were FAM (6-carboxyfluorescein) and TAMRA (6-carboxy-tetramethylrhodamine), in 5′- and 3′-end, respectively. The lyophilized fluorolabeled hTelo (21-mer), d[GGG(TTAGGG)_3_], *cKIT1*, d[GGGAGGGCGCTGGGAGGAGGG], *cKIT2*, d[CCCGGGCGGGCGCGAGGGAGGGGAGG], *BCL2* d[AGGGGCGGGCGCGGGAGGAAGGGGGCGGGAGCGGGGCTG], and *hTERT* d[GGGGGCTGGGCCGGGGACCCGGGAGGGGTCGGGACGGGGCGGGG] oligonucleotides were first diluted in deionized water to obtain 100 μM stock solutions. Stock solutions were diluted to a concentration of 400 nM in potassium cacodylate buffer (60 mM, pH 7.4) and then annealed to form G-quadruplex (G4) structures by heating to 95 °C for 5 min, followed by cooling to room temperature overnight.

Experiments were carried out in a 96-well plate with a total volume of 30 μL. The final concentration of the G4-oligonucleotide was set to 200 nM in potassium cacodylate buffer (60 mM, pH 7.4). Stock solutions of the gold compounds in DMSO (1 mM) were freshly prepared prior to the experiments. The stock solutions were further diluted to a final concentration of 2 μM (with a total percentage of DMSO of approx. 0.1%) in potassium cacodylate buffer (60 mM, pH 7.4) to achieve the desired G4/gold compound stoichiometry (from 1:1 to 1:5). Competition assays with duplex DNA were performed using *calf-thymus* DNA (Sigma-Aldrich, Germany) according to a previously reported procedure.^58^ In all the experiments, to compare different sets of data, FAM emission was normalized (0–1). Melting temperature is defined as the temperature at which the normalized emission is 0.5, and Δ*T*_m_ is defined as the difference of *T*_m_ between treated samples and untreated controls. Independent experiments were run at least in triplicates.

### Gibbs Free Energy Analysis from FRET DNA Melting Data and Fitting

In order to determine the energy of binding of the gold compound to the G4, the experimental data were normalized to folded fraction (θ) of G4-DNA and fitted according to eqs 1 and 2,^79^ where the enthalpy (Δ*H*) for the process was derived from the resulting fit. In eq 2, *T* corresponds to the temperature (variable during the melting assay) and R is the gas constant. Subsequently, Δ*G*_300_ was derived from eq 3,^80^ where T_MD_ corresponds to the temperature used in the metaD simulations described above (300 K) and *T*_m_ is the melting temperature for each condition, determined from the FRET melting profiles.
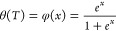
1where
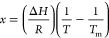
2
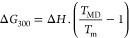
3

### CD Spectroscopy

CD spectra of G4 structures alone or in the presence of the Au(I) compounds were recorded on a JASCO J-1500 CD spectrometer equipped with a PTC-517 Peltier thermostated cell holder, using the following parameters: range 210–400 nm, bandwidth 1 nm, step 1 nm, accumulation 3, temperature 25 °C. Stock solutions of unlabeled 21-mer hTelo, d[GGG(TTAGGG)3], and *cKIT1*, d[GGGAGGGCGCTGGGAGGAGGG] (each 100 μM), were prepared by dissolving the lyophilized sample in deionized water and further diluted in a Tris-HCl/KCl buffer (10/50 mM, pH 7.4). Stock solutions of **AuB3** and **AuC3** (1 mM) in DMSO were freshly prepared prior to the experiments and further diluted in buffer to achieve a G4/gold compound stoichiometry up to 1:10.

For CD G4 melting experiments, a stock solution containing 0.2 nM c*KIT1* was diluted to a concentration of 8.0 μM in 60 mM potassium cacodylate buffer (pH 7.4) and then annealed to form G4 structures as described in the FRET paragraph. Stock solutions of **AuB2**, **AuB3**, or **AuC3** (10 mM in DMSO) were diluted in 60 mM potassium cacodylate buffer to a concentration of 40 μM. The final concentration of G4 in each experiment was set to 4.0 μM with a ratio G4/metal complex of 1:5 (%_DMSO_ = 0.2%). A determination of the melting curve of the pure G4 structure was performed using a solution with c_*cKIT1*_ = 4.0 μM, and DMSO (0.2%). The CD spectra of each sample were measured in the range of 25–95 °C with 2 °C temperature steps, the measurements were blank-corrected using in 60 mM potassium cacodylate buffer (pH 7.4) containing 0.2% DMSO. Each spectrum was acquired in the range of 240–400 nm with a 1 nm spectral resolution and integration time of 1 s.

### MetaD Simulations

The crystal structures of **AuB3** (CCDC number: 2202920) and of the *cKIT1* G4 model (PDB ID: 4WO2) were used for the simulations. The system **AuB3**–G4 adduct was built using the Maestro GUI from Schrödinger (build 2021-1)^81^ and all metaD calculations were undertaken using the Desmond simulation package from Schrödinger LLC^82,83^ (ref). The OPLS 2005 force field parameters were used in all simulations.^84^ The system consisted of cKIT1 with **AuB3** placed at a distance of ca. 10 Å from the upper tetrad, the box size being 10 × 10 × 10 nm and solvated using the TIP3P water model (ca. 32,000).^85^ 16 Na^+^ ions were added to neutralize the charge of the system, with a further addition of 0.6 mol of K^+^ and Cl^–^ ions added to match FRET DNA melting experimental conditions of pH 7.4. Constraints were added between the potassium ions embedded in the G4 structure and the O6 oxygen of each guanine in the three stacked tetrads to mimic the structurally relevant affinities of the G4. The system was first relaxed using the Desmond minimization application for 100 ps and further equilibrated using the standard relax model option for MD/MetaD before each simulation run. Simulations were run using the *NPT* ensemble with a Nose–Hoover^86^ thermostat (300 K) and MTK^87^ barostat. The time step for all production runs was 1fs with a simulation time of 50 ns (20 production runs in total) for a combined 1 ms of simulation time. A random seed was used for each production run. The chosen CVs were the distance between each of the two gold centers of **AuB3** and the upper potassium ion located at the center of cKIT1, with a Gaussian width of 0.05 Å and height of 0.03 kcal/mol and a deposition rate of 0.09 ps. FES were obtained in the form of a heat map for each run with the Δ*G* taken from the lowest energy minima of each FES. The average Δ*G* was then calculated, from these values (Table S6). Δ*G* values from 19 of the 20 simulations were used, while an outlier of both energy and position was omitted.

### Cell Culture Maintenance

MDA-MB-231 human breast cancer cells, MCF-7 human breast carcinoma cells, and VERO E6 *Cercopithecus aethiops* monkey kidney cells were maintained in Dulbecco’s modified Eagle’s medium (high glucose, pyruvate, and no glutamine), which was supplemented with heat-inactivated fetal bovine serum (qualified, South American origin, 10% v/v), gentamicin sulfate solution 50 mg/mL (1% v/v, 50 mg/L end concentration), and l-glutamine solution 200 nM (1% v/v, 2 nM end concentration) and were passaged twice a week. A2780 human ovarian cancer cells were maintained in the RPMI 1640 medium (no glutamine), supplemented with a heat-inactivated fetal bovine serum (qualified, South American origin, 10% v/v), penicillin/streptomycin solution 10,000:10,000 U/mL (1% v/v, 5 mL), and l-glutamine solution 200 nM (1% v/v, 2 nM end concentration) and were also passaged twice a week. All reagents were purchased from Gibco at Thermo Scientific and solvents from Sigma-Aldrich if not stated differently. Ultrapure water (18.2 MΩ/0.56 μS at 25 °C) was provided by a BerryPuremini from Berrytec. MCF-7 and VERO E6 cells were obtained from Cell Line Service (CLS, Eppelheim, Germany), MDA-MB-231 cells were from the Helmholtz Institute for Infection Research (HZI, Braunschweig, Germany), and A2780 cells from the European Collection of Authenticated Cell Cultures (ECACC, United Kingdom/distributed by Sigma Aldrich, Germany).

### Antiproliferative Activity Studies

A2780 cells (5000 cells/well), MDA-MB-231 cells (8000 cells/well), MCF-7 cells (10,000 cells/well), or Vero E6 cells (10,000 cells/well) were transferred to a flat-bottom 96-well microtiter plate and incubated at 37 °C/5% CO_2_ in a humidified atmosphere for 24 h. Stock solutions of the compounds in DMF or DPBS (cisplatin) were freshly prepared and diluted with the respective cell culture medium to graded concentrations (a final concentration of DMF: 0.1% v/v). After 72 h of exposure, the cell biomass of living cells was quantified by 3-(4,5-dimethylthiazol-2-yl)-2,5-diphenyl tetrazolium bromide (Sigma-Aldrich) staining and the absorptions were calculated as the delta of two measurements of the same well at wavelengths 570 and 690 nm by an Infinite 200pro plate reader (Tecan). The EC_50_ values (half-maximal effective concentrations) were determined as the concentration that caused 50% inhibition of cell proliferation compared to the untreated control (0.1% DMF in DMEM/RPMI). The results were calculated as the mean of at least three independent experiments.

## Supporting Information Available

NMR and HR-ESI-MS spectra of the newly synthesized compounds, ^1^H NMR stability tests, representative FRET melting profiles, and crystal and MetaD calculation data (PDF)
MetaD simulation of **AuB3** interaction with *cKIT1* (AVI)

## Supplementary Material

ic2c03041_si_001.pdf

ic2c03041_si_002.avi
