# Supplementary material for: “Dynamical Docking” of Cyclic Dinuclear Au(I) Bis-N-heterocyclic Complexes Facilitates Their Binding to G-Quadruplexes
Source: Inorg Chem. 2022 Dec 9;61(50):20405–23. doi: 10.1021/acs.inorgchem.2c03041 (PMC9953335; doi:10.1021/acs.inorgchem.2c03041)
Supplement: Supplementary file 1 — ic2c03041_si_001.pdf [file ic2c03041_si_001.pdf]

Supporting Information

"Dynamical Docking" of Cyclic Dinuclear Au(I) Bis-N-heterocyclic Complexes Facilitates Their Binding to G-Quadruplexes

Clemens Kaußler,[†,a] Darren Wragg,[†,a] Claudia Schmidt,[a] Guillermo Moreno-Alcántar,[a] Christian Jandl,[b] Johannes Stephan,[b] Roland A. Fischer,[b,c] Stefano Leoni,[d] Angela Casini*[,a] and Riccardo Bonsignore,*[,e]

[a] *Chair of Medicinal and Bioinorganic Chemistry, Department of Chemistry, Technical University of Munich, Lichtenbergstraße 4, D-85748 Garching b. München, Germany.*

[b] *Catalysis Research Center & Department of Chemistry, Technische Universität München, Ernst-Otto-Fischer Str. 1, D-85748 Garching b. München, Germany.*

[c] *Chair of Inorganic and Metal-Organic Chemistry, Department of Chemistry, Technische Universität München, Ernst-Otto-Fischer Str. 1, D-85748 Garching b. München, Germany.*

[d] *School of Chemistry, Cardiff University, Park Place, CF10 3AT Cardiff, UK*

[e] *Dipartimento di Scienze e Tecnologie Biologiche, Chimiche e Farmaceutiche, Università degli Studi di Palermo, Viale delle Scienze, Edificio 17, 90128 Palermo, Italy.*

† shared first authors

* angela.casini@tum.de; riccardo.bonsignore@unipa.it

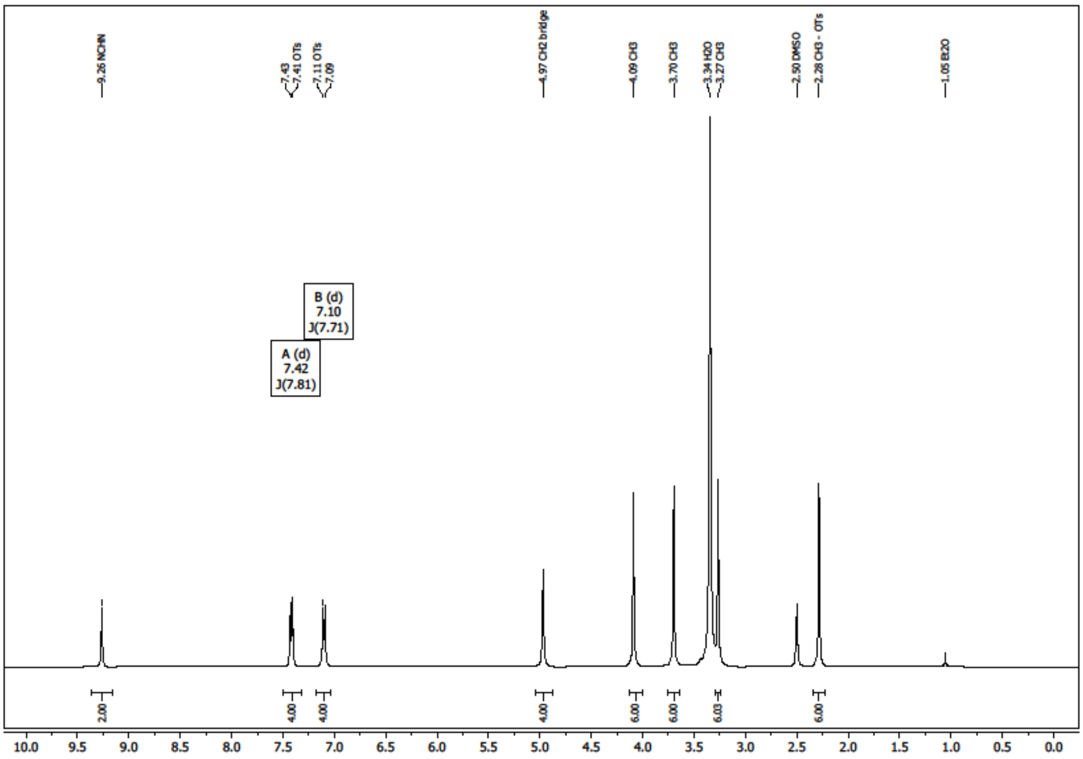

Figure S1. ^{1}**H NMR of** H_2B2^{OTs} **(DMSO-*d*$_6$).**

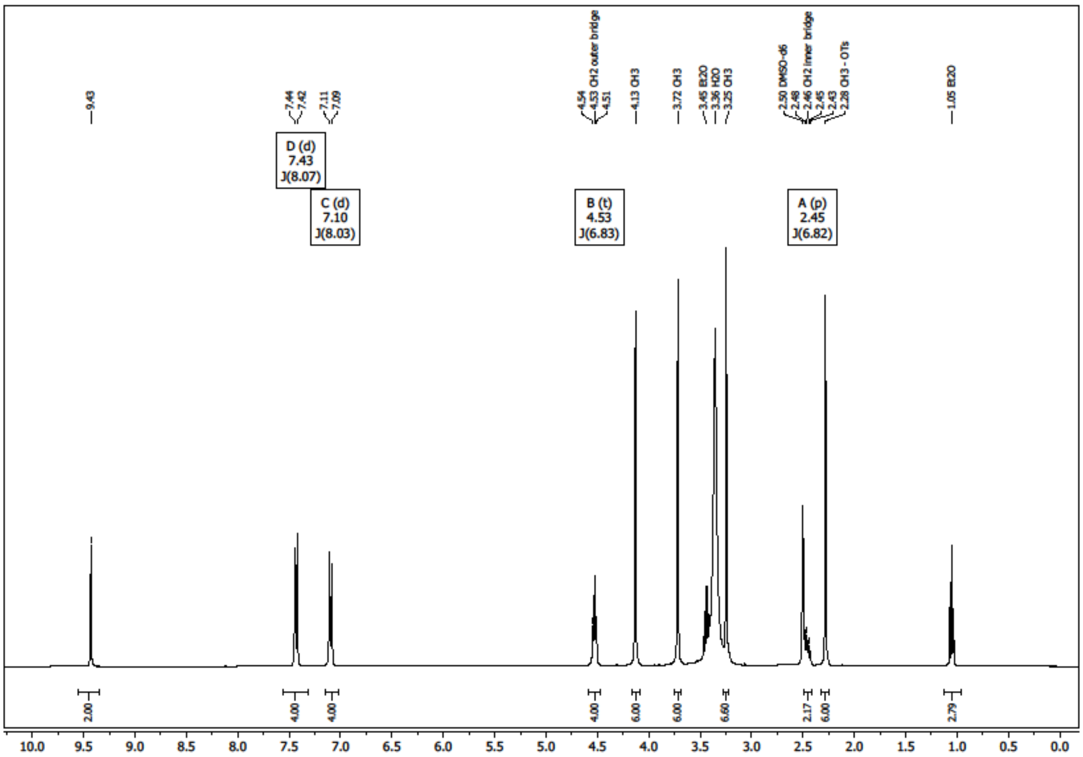

Figure S2. ^{1}**H NMR of** H_2B3^{OTs} **(DMSO-*d*$_6$).**

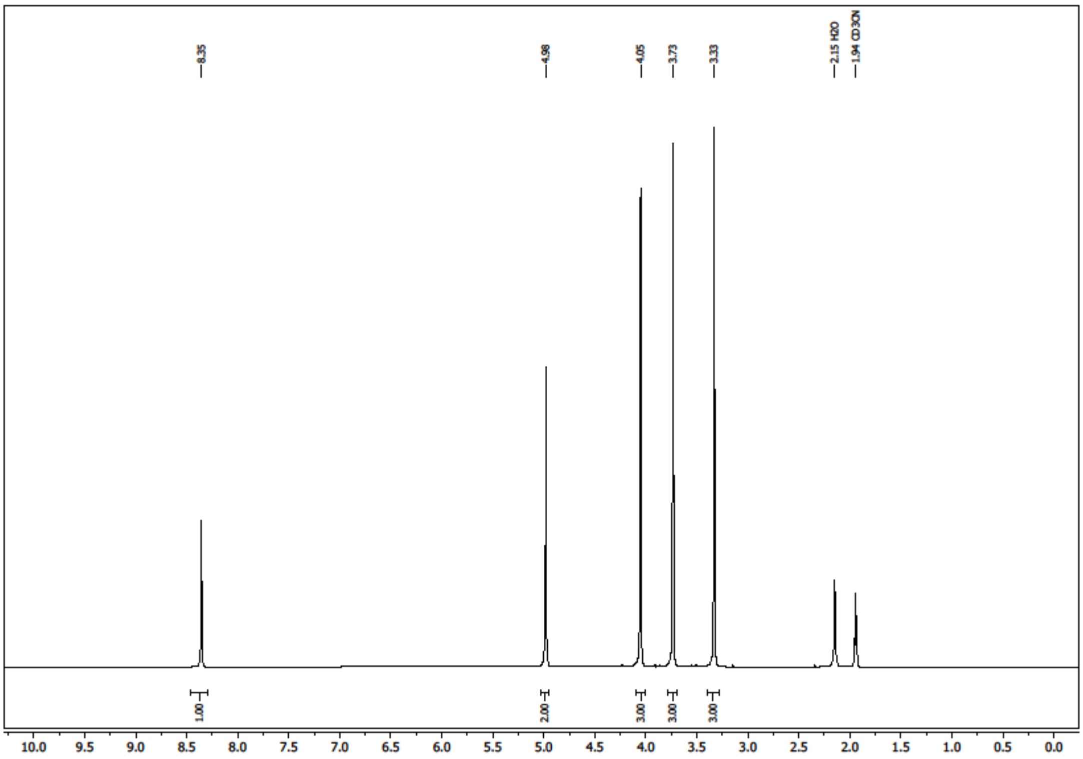

Figure S3. ^{1}H **NMR of** H_2B2^{PF6} **(CD_3CN).**

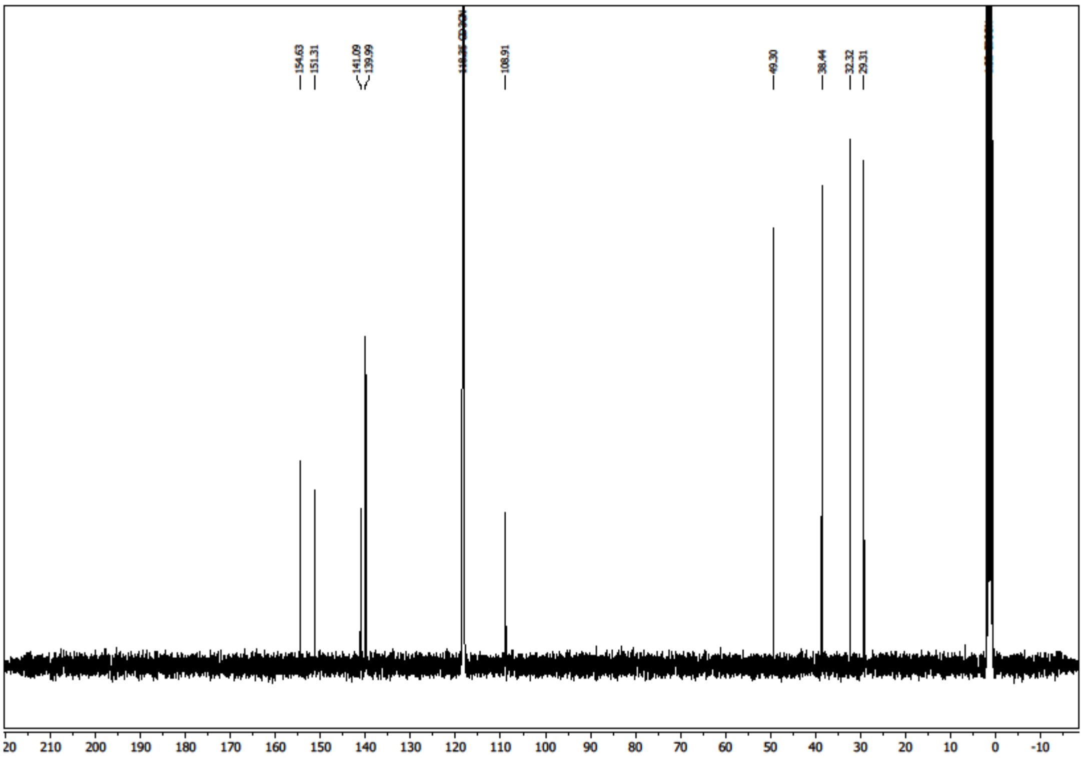

Figure S4. $^{13}C\{^{1}H\}$ **NMR of** H_2B2^{PF6} **(CD_3CN).**

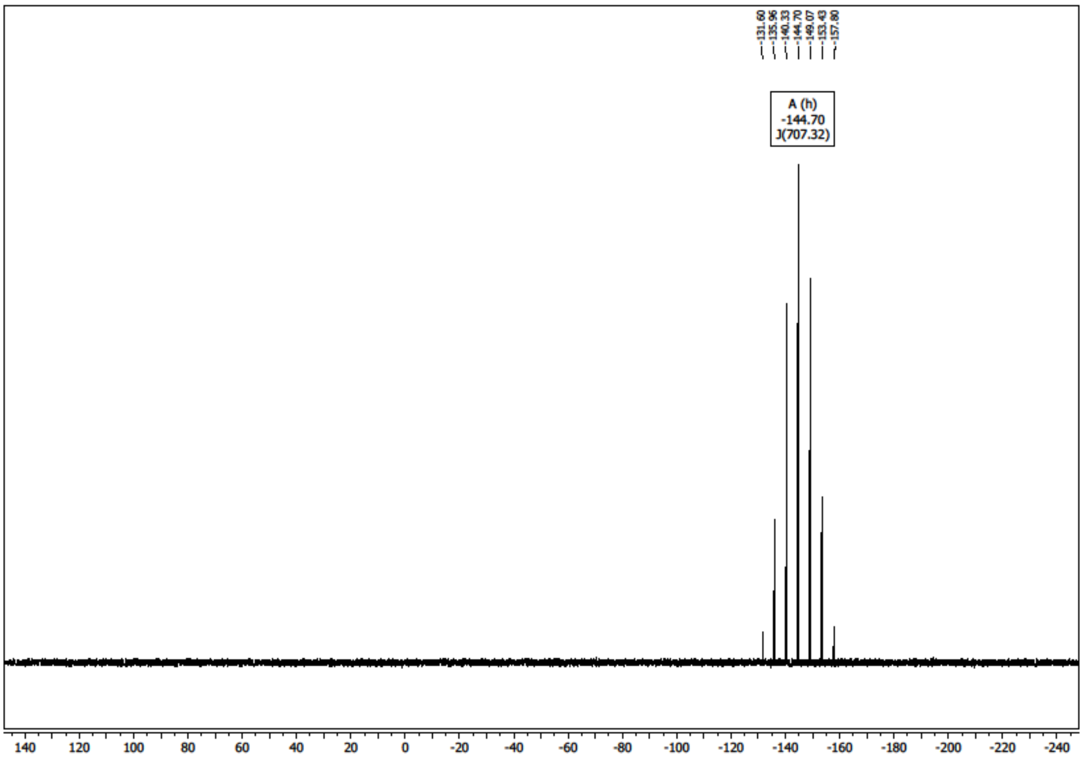

Figure S5. ^{31}P **NMR of** H_2B2^{PF6} **(**CD_3CN**).**

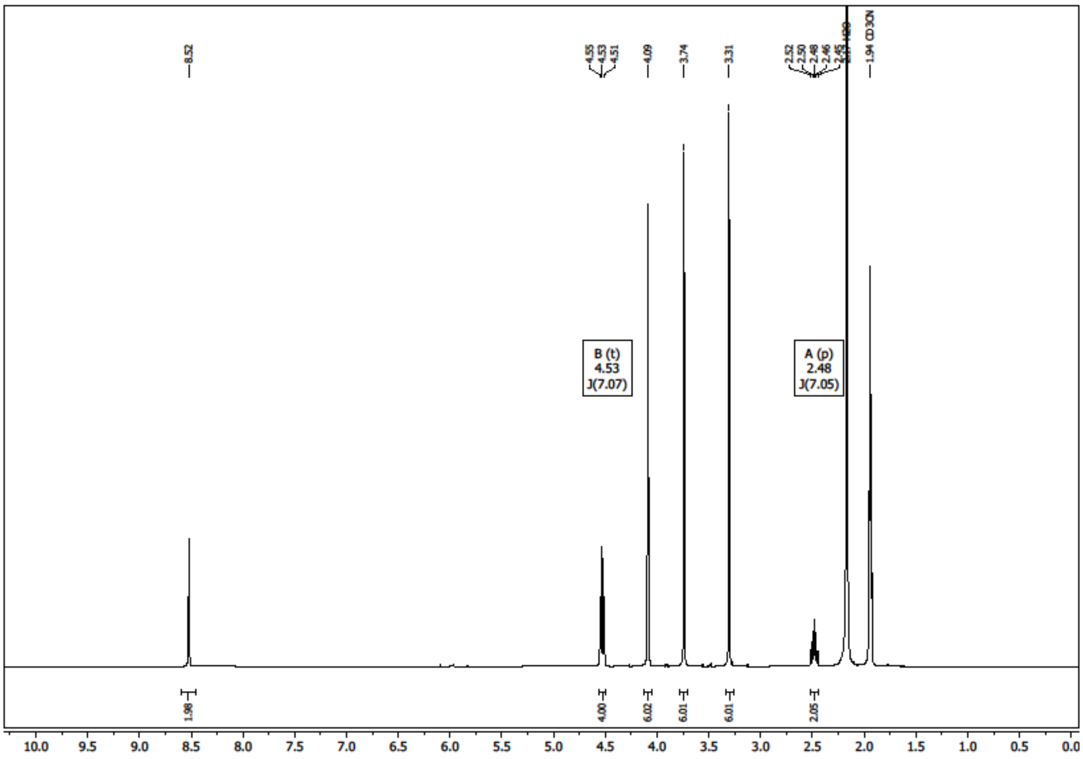

Figure S6. ^{1}H **NMR of** H_2B3^{PF6} **(**CD_3CN**).**

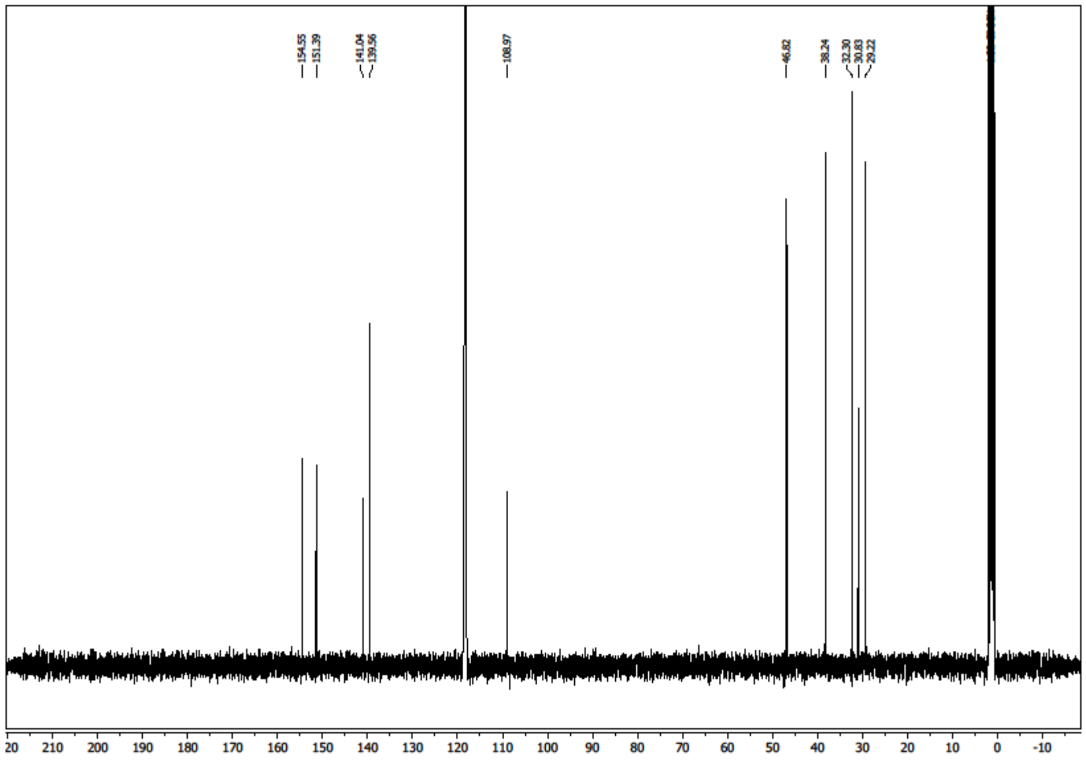

Figure S7. $^{13}C\{^{1}H\}$ **NMR of** H_2B3^{PF6} **(**CD_3CN**).**

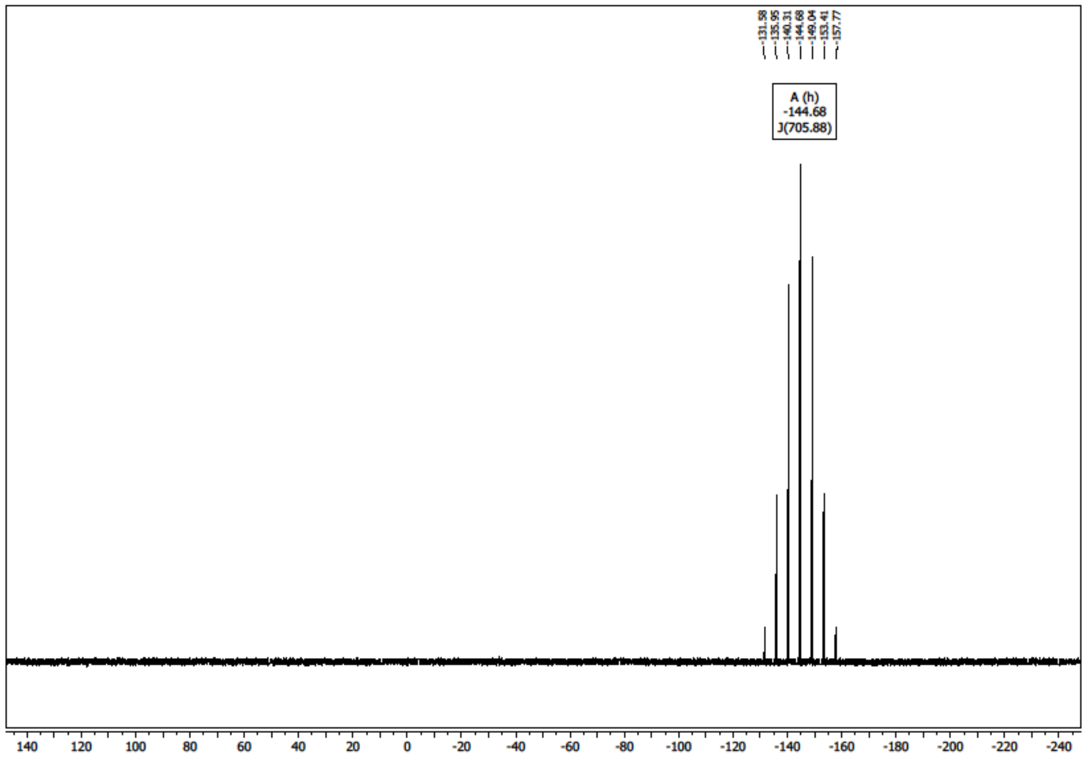

Figure S8. ^{31}P **NMR of** H_2B3^{PF6} **(**CD_3CN**).**

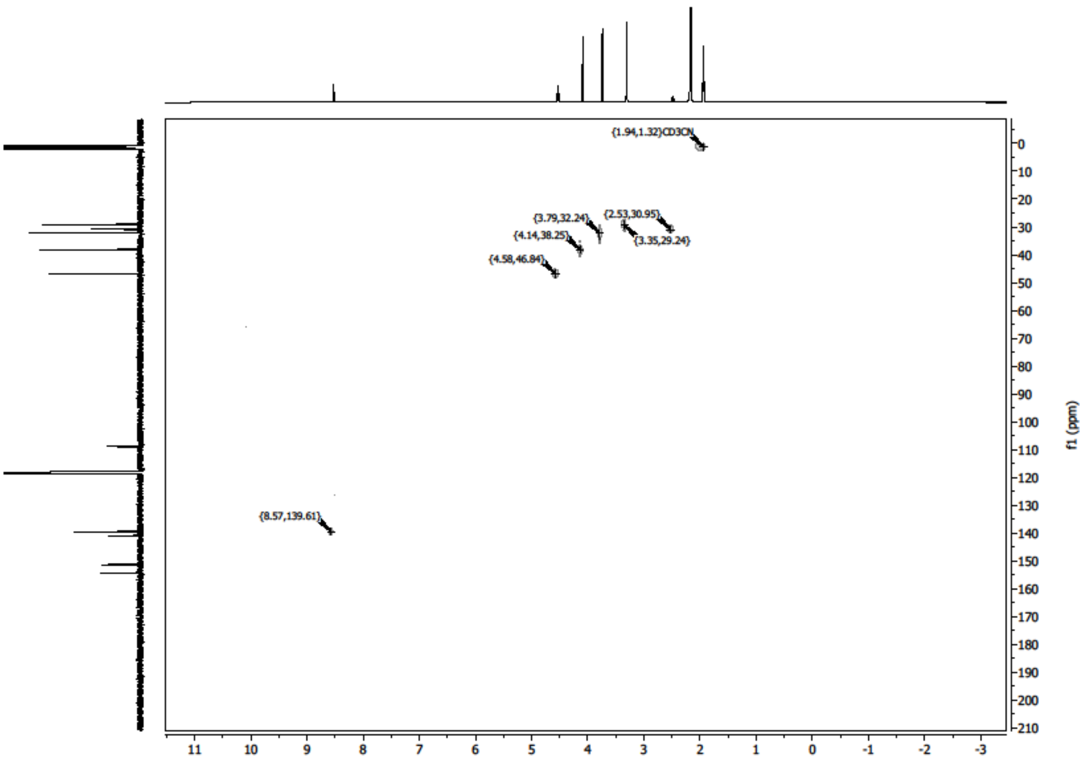

Figure S9. ^{1}H—^{13}C **HSQC NMR of** H_2B3^{PF6} **(**CD_3CN**).**

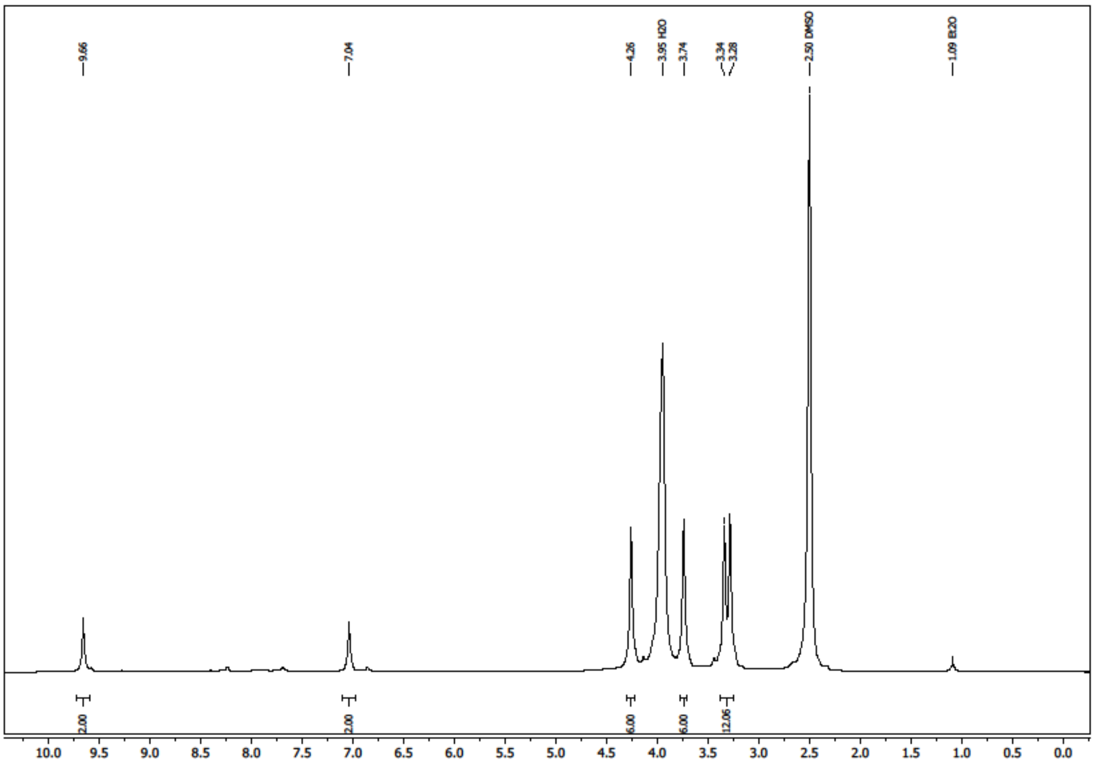

Figure S10. ^{1}H **NMR of** H_2B1^{MeSO4} **(DMSO-**d_6**).**

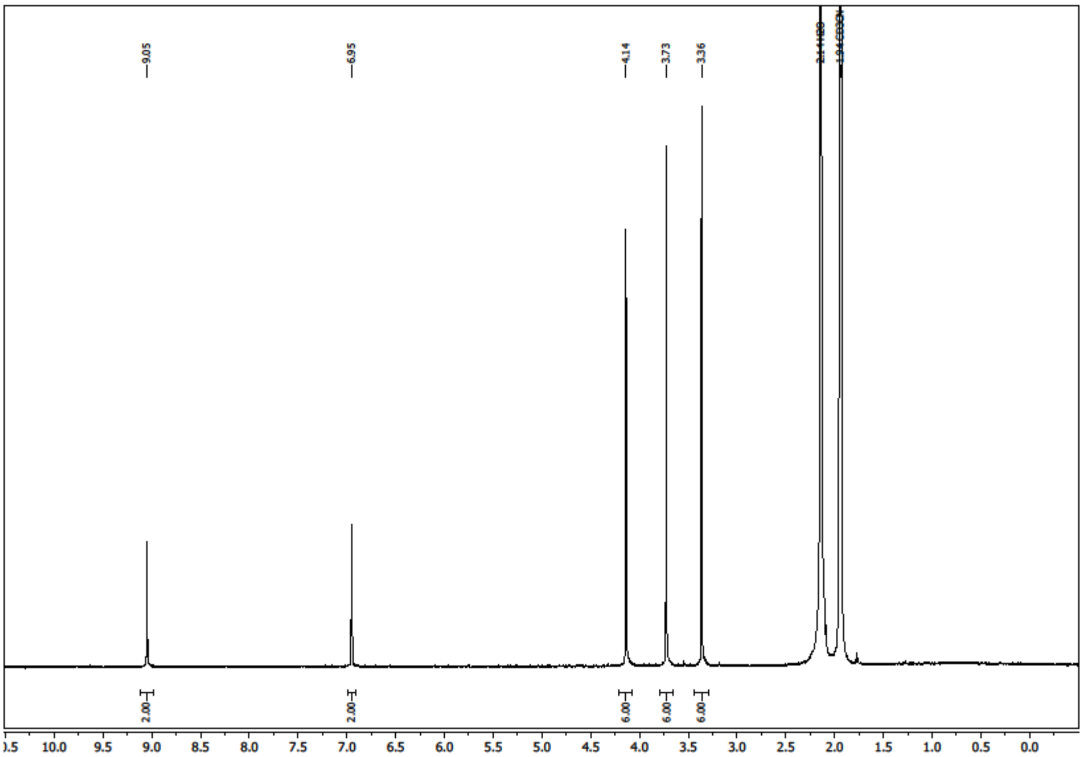

Figure S11. ^{1}**H NMR of** H_2B1^{PF6} **(**CD_3CN**).**

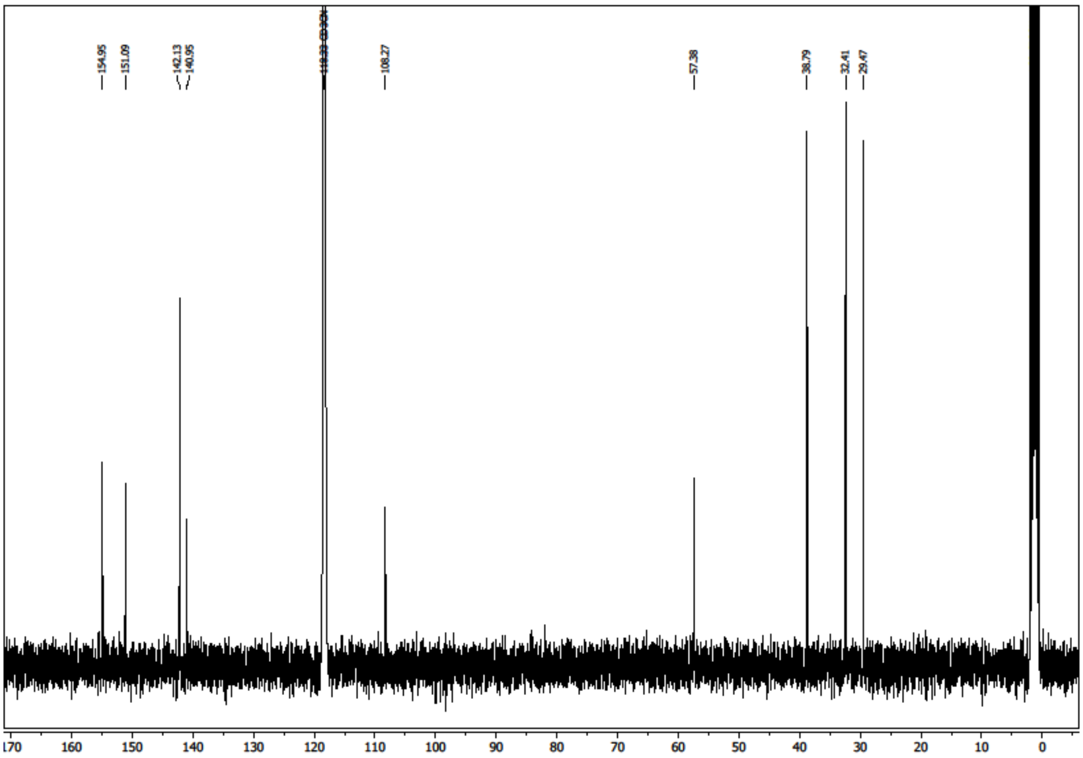

Figure S12. $^{13}C\{^{1}H\}$ **NMR of** H_2B1^{PF6} **(**CD_3CN**).**

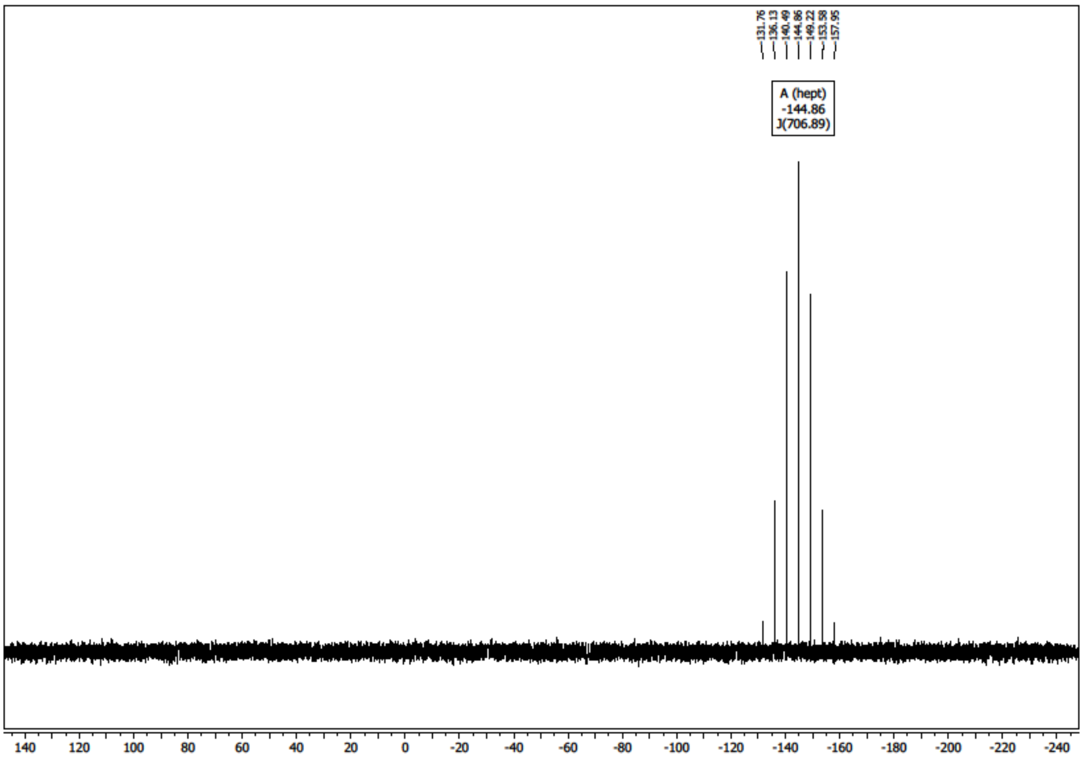

Figure S13. **^{31}P NMR of H_2B1^{PF6} (CD_3CN).**

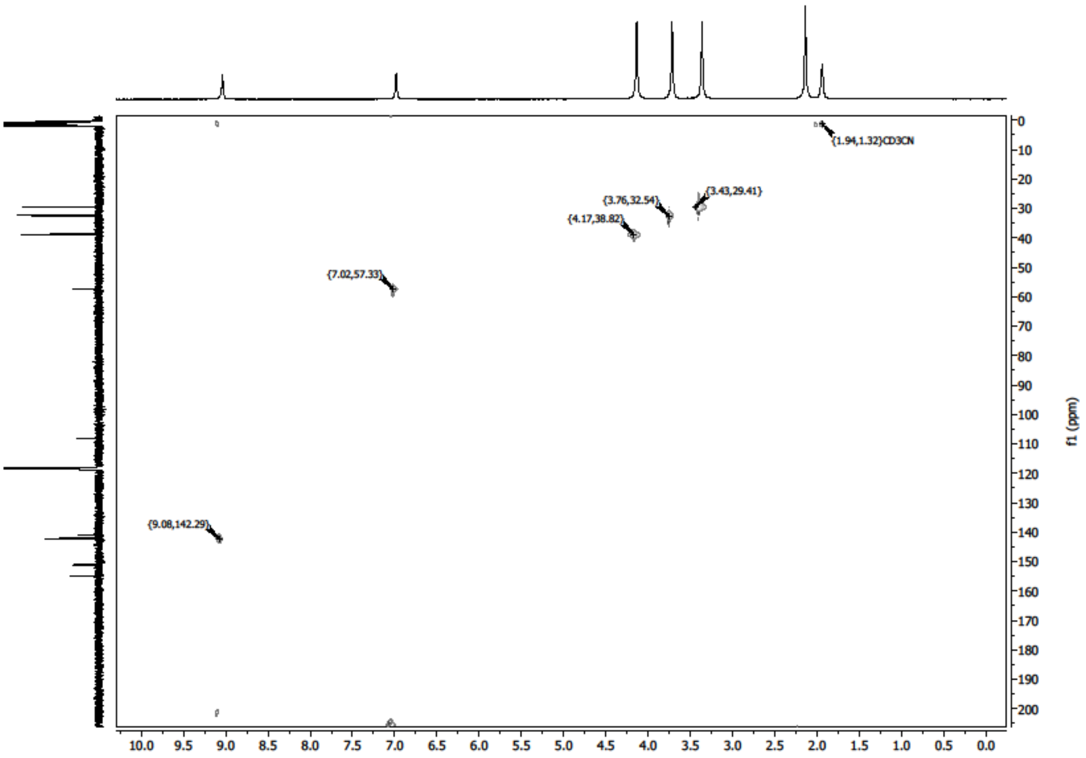

Figure S14. **^1H—^{13}C HSQC NMR of H_2B1^{PF6} (CD_3CN).**

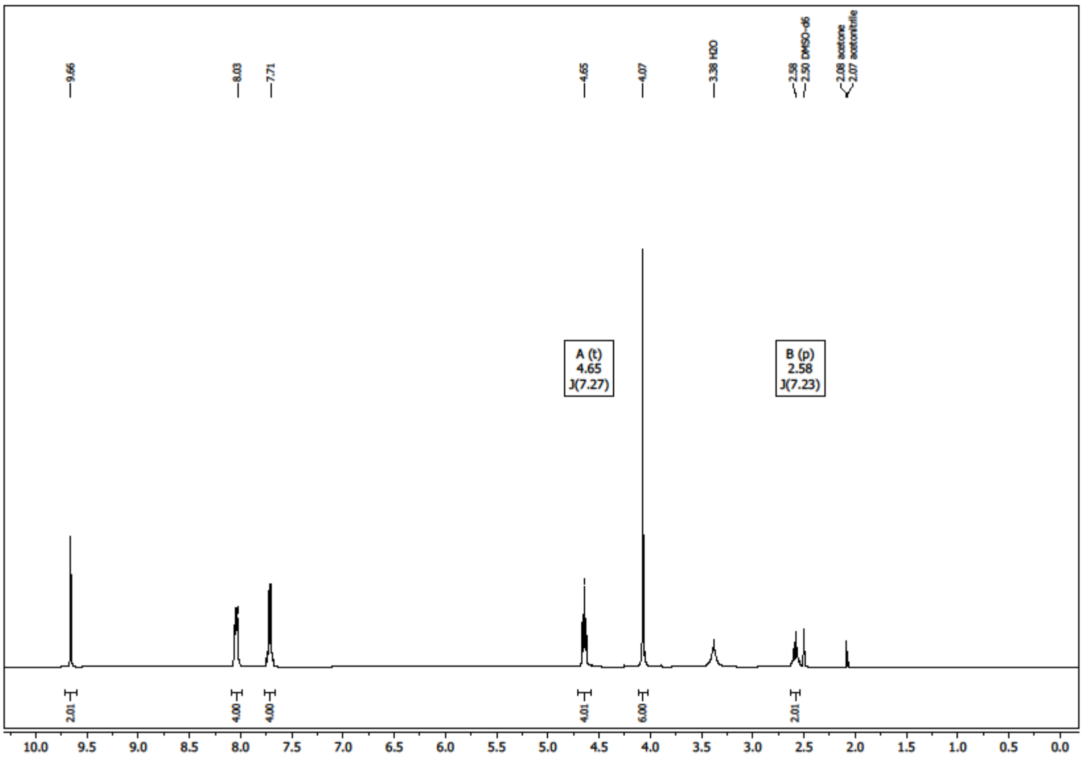

Figure S15. ^{1}H **NMR of** H_2C3^{BF4} **(DMSO-*d*$_6$).**

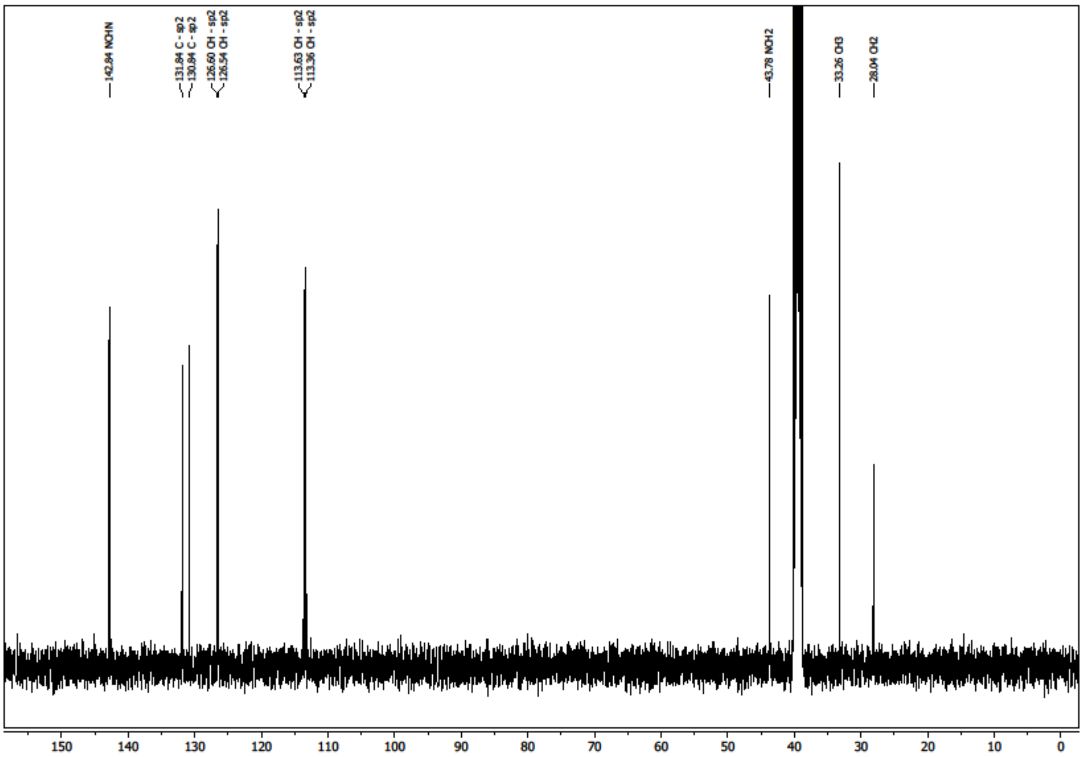

Figure S16. $^{13}C\{^{1}H\}$ **NMR of** H_2C3^{BF4} **(DMSO-*d*$_6$).**

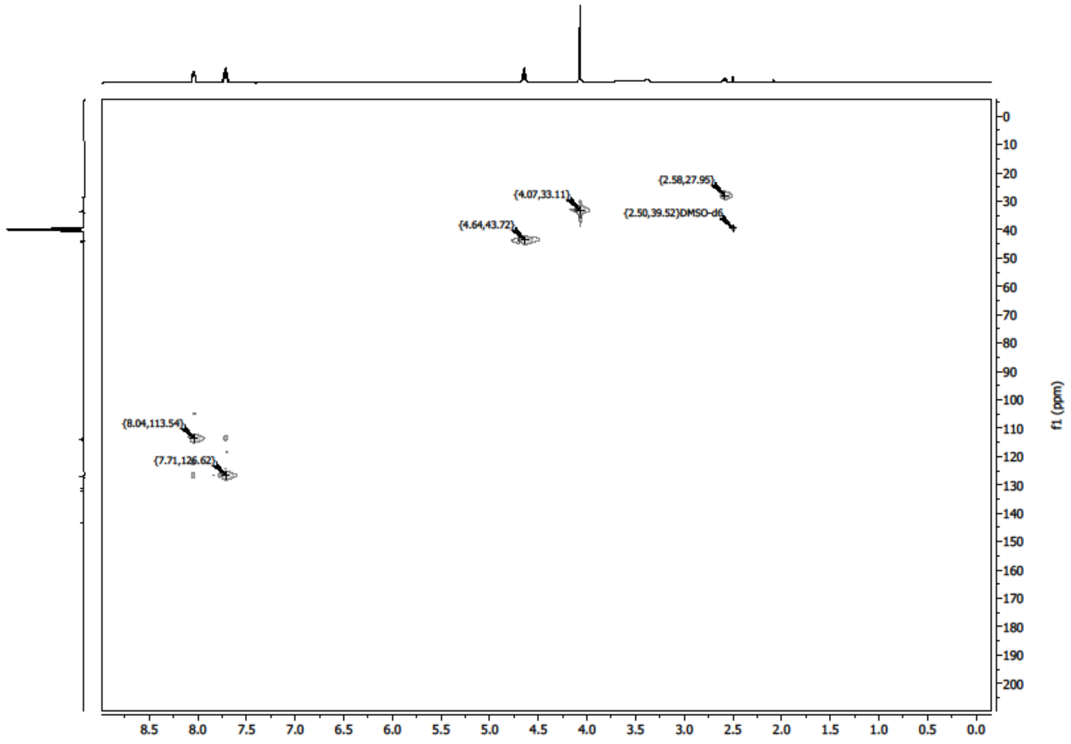

Figure S17. ^{1}H—^{13}C **HSQC NMR of** H_2C3^{BF4} **(DMSO-*d*$_6$).**

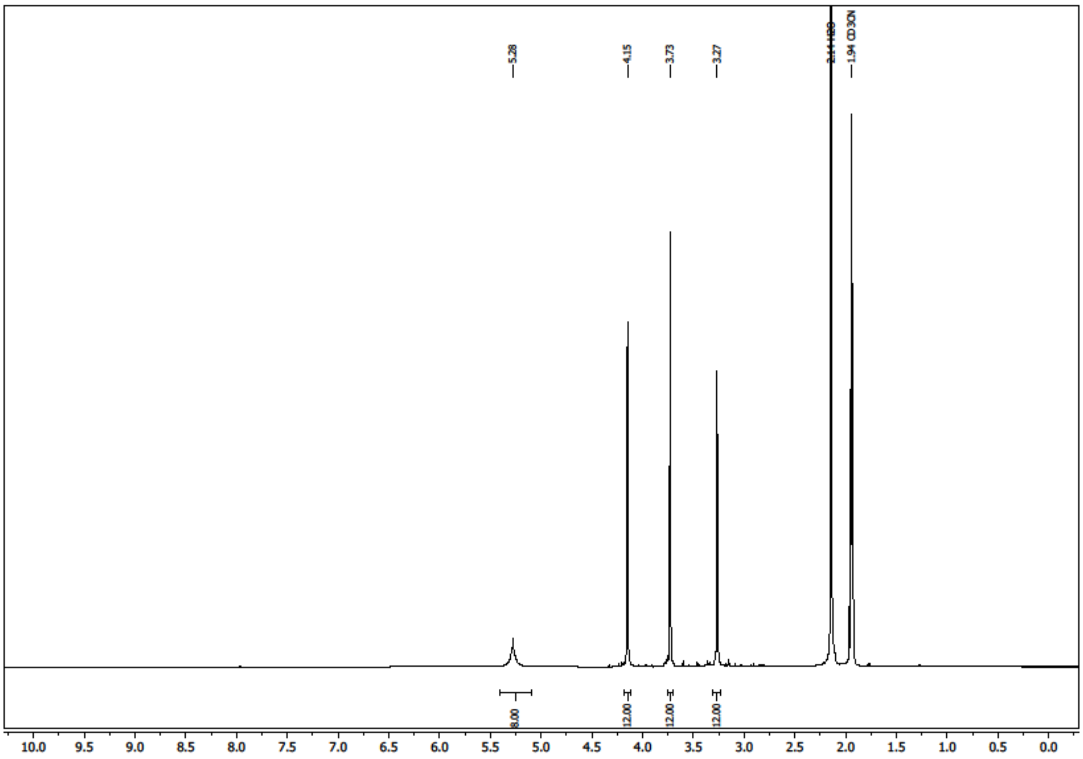

Figure S18. ^{1}H NMR of AuB2 (CD_3CN).

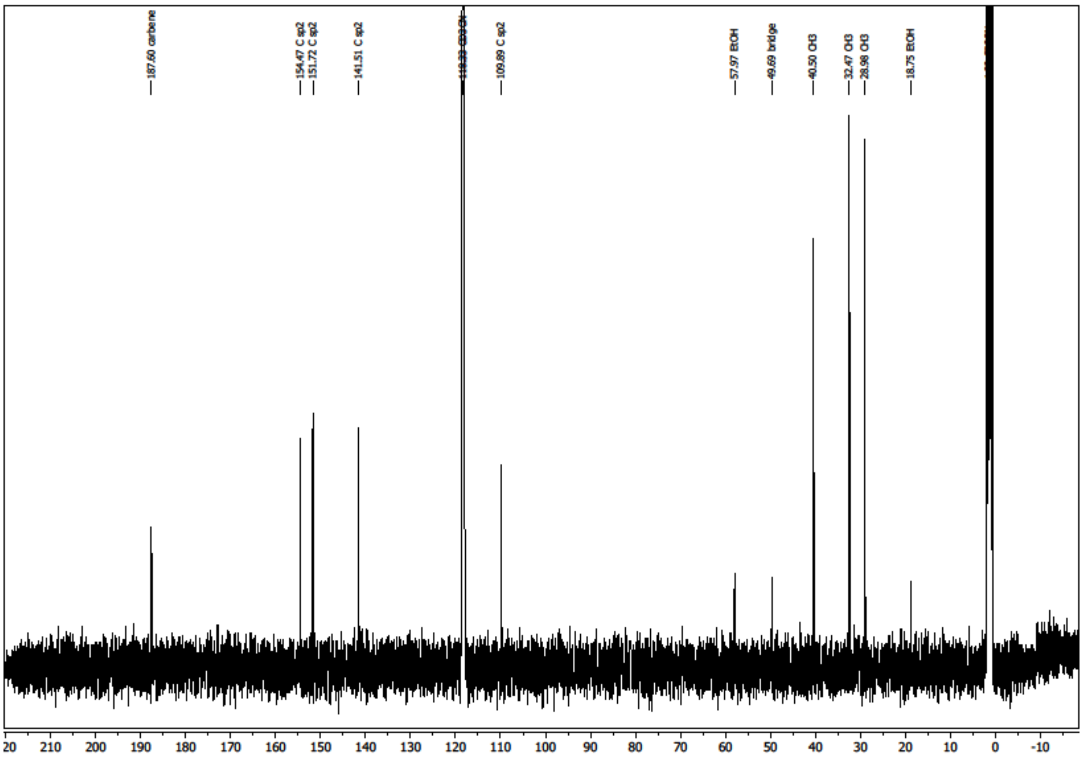

Figure S19. $^{13}C\{^1H\}$ NMR of AuB2 (CD_3CN).

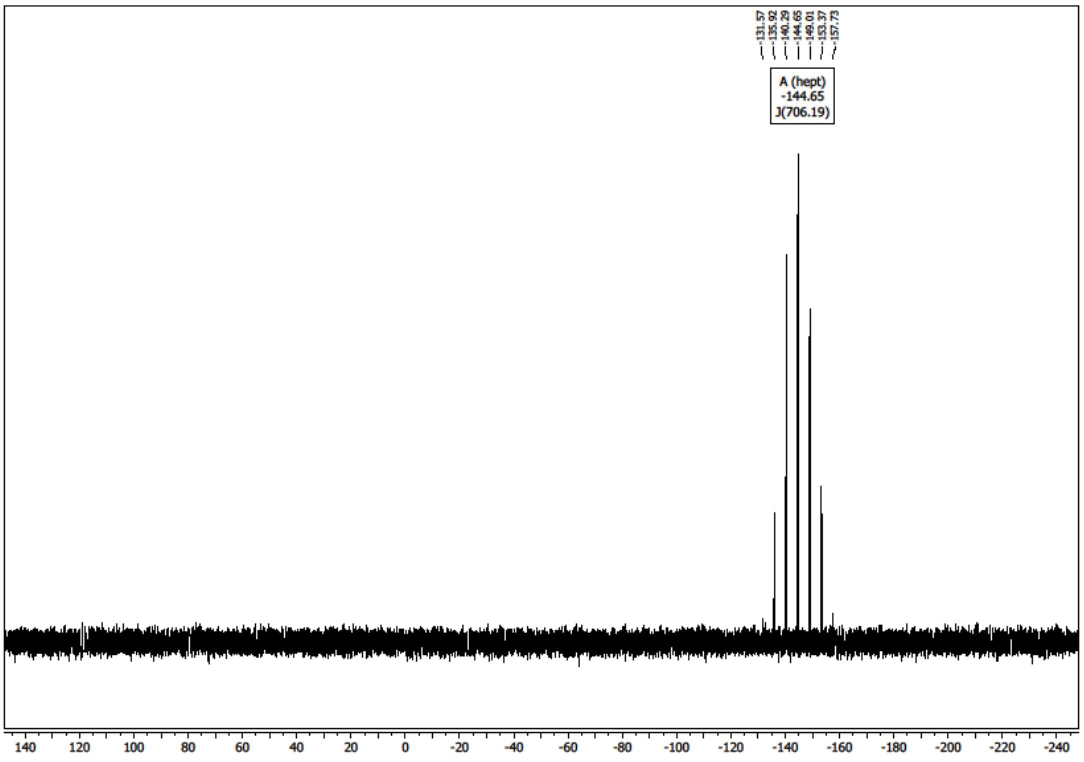

Figure S20. ^{31}P **NMR of AuB2 (CD_3CN).**

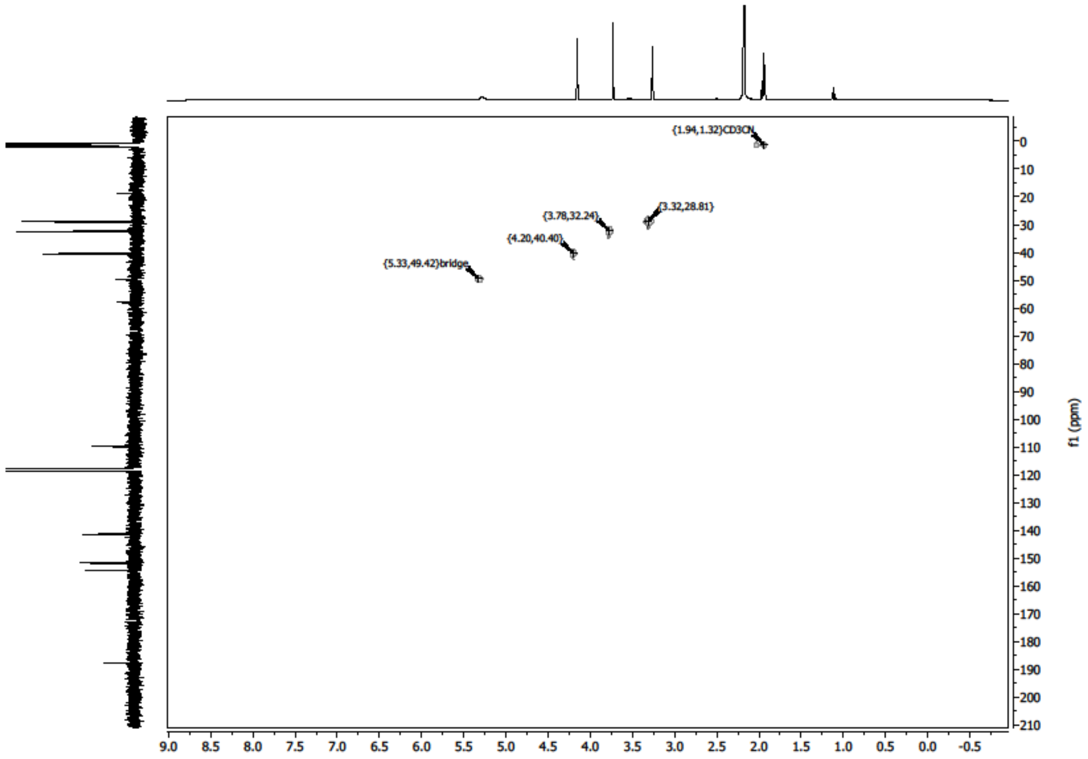

Figure S21. ^{1}H**—**^{13}C **HSQC NMR of AuB2 (CD_3CN).**

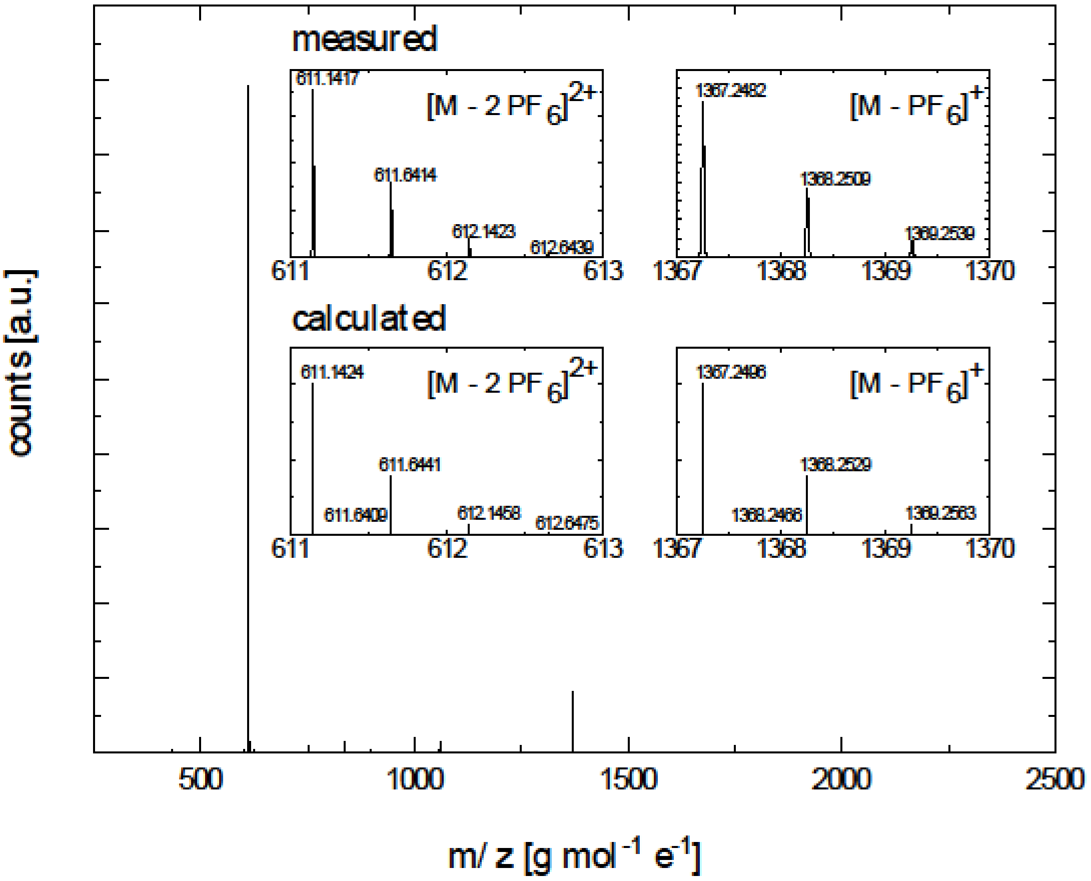

Figure S22. HR-ESI-MS of AuB2.

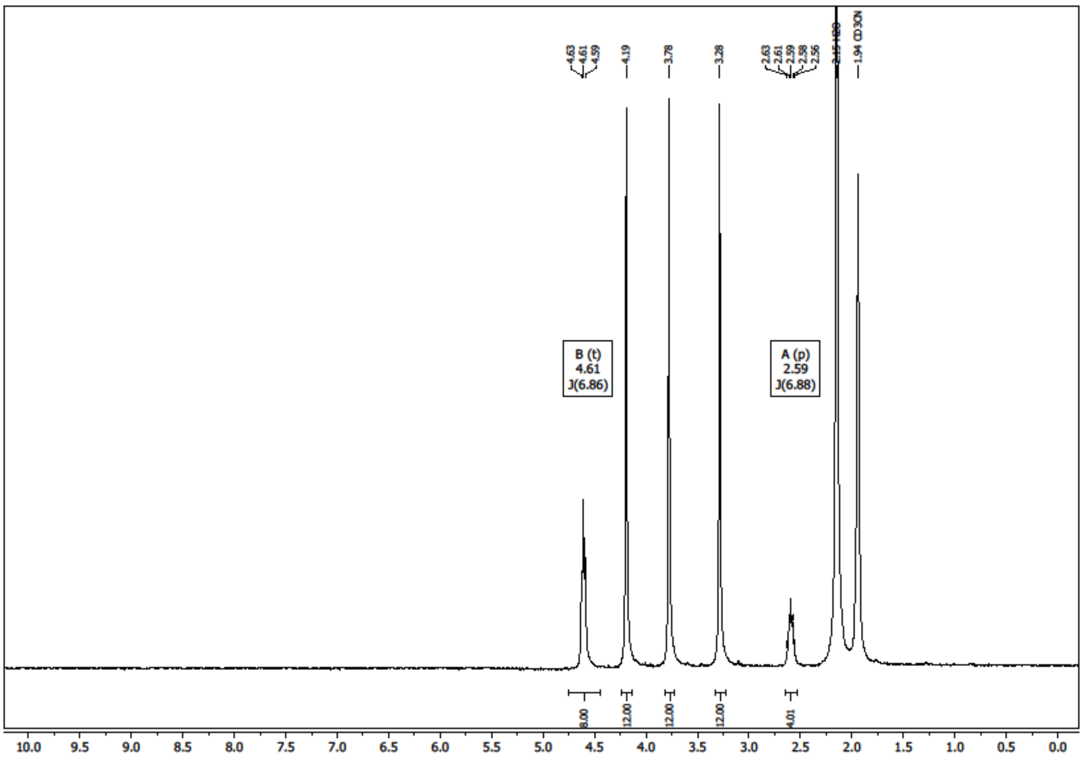

Figure S23. ^{1}H NMR of AuB3 (CD_3CN).

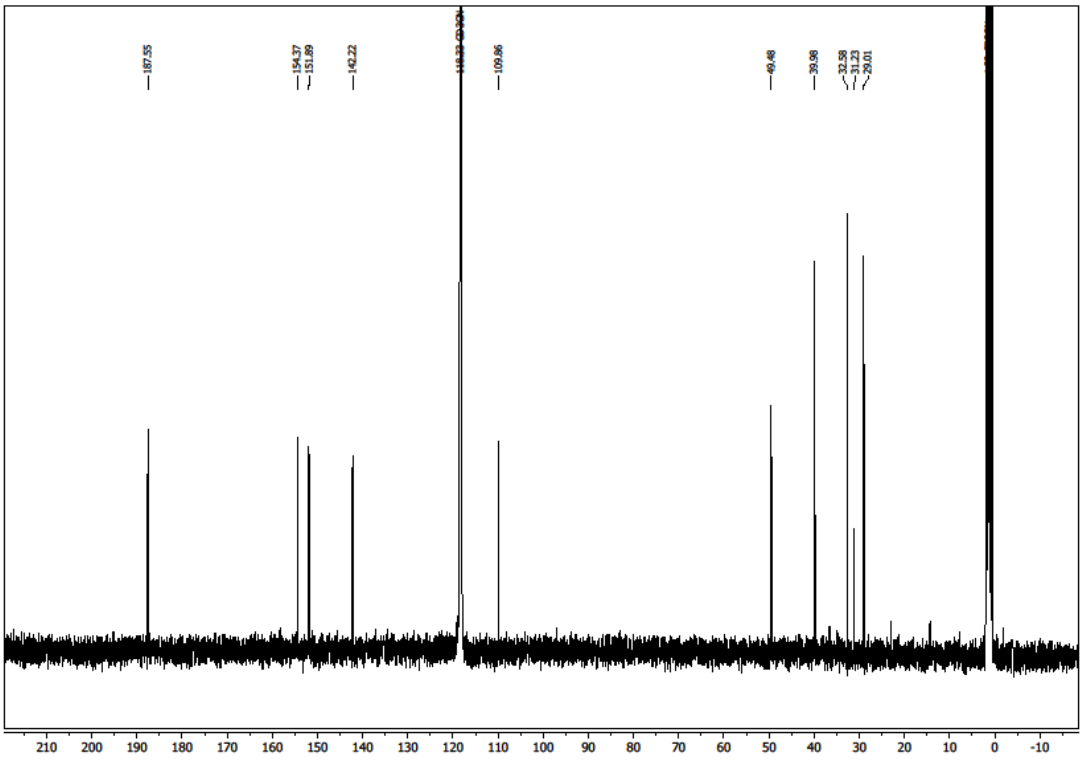

Figure S24. $^{13}C\{^{1}H\}$ **NMR of AuB3 (CD_3CN).**

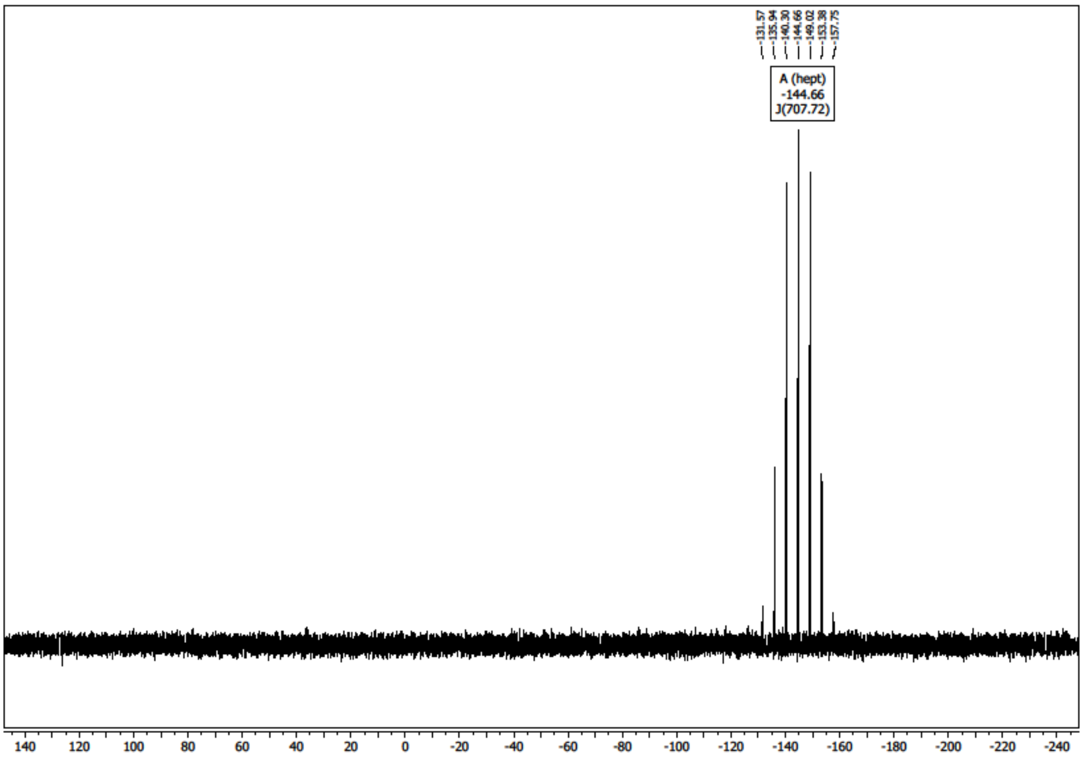

Figure S25. ^{31}P **NMR of AuB3 (CD_3CN).**

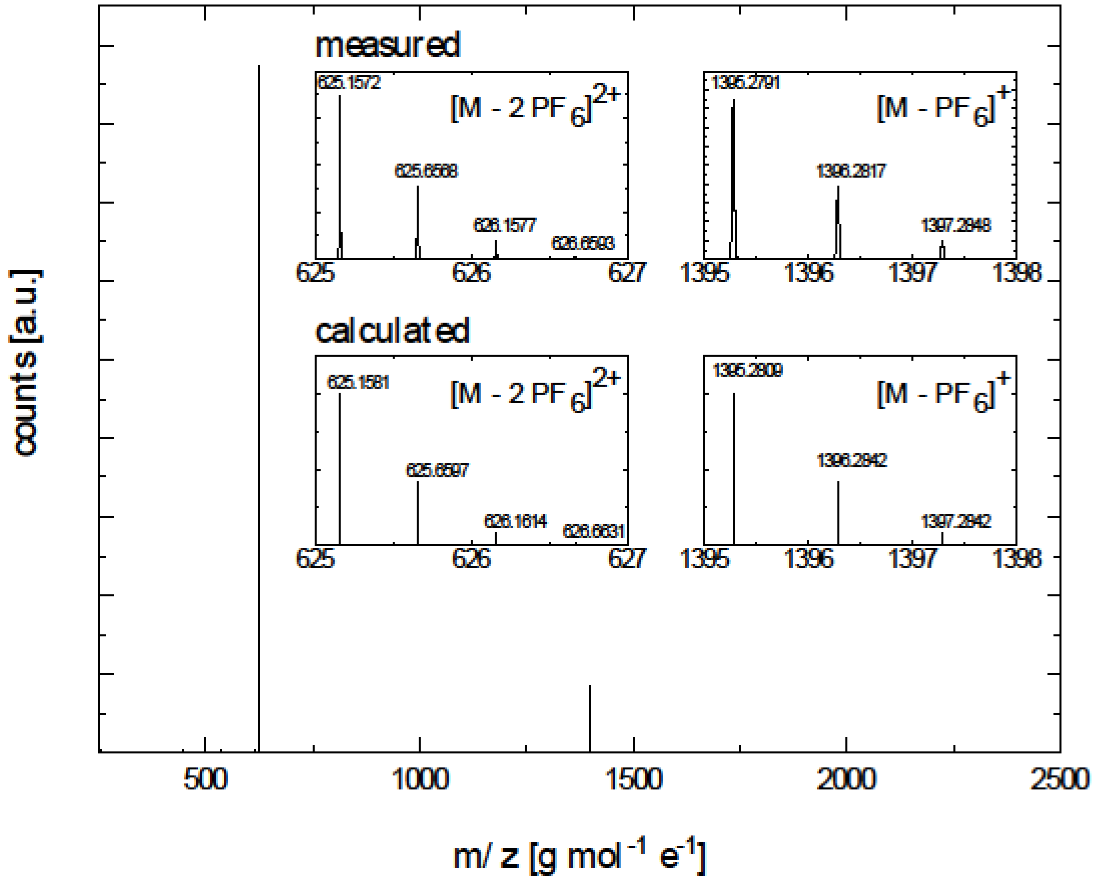

Figure S26. HR-ESI-MS of AuB3.

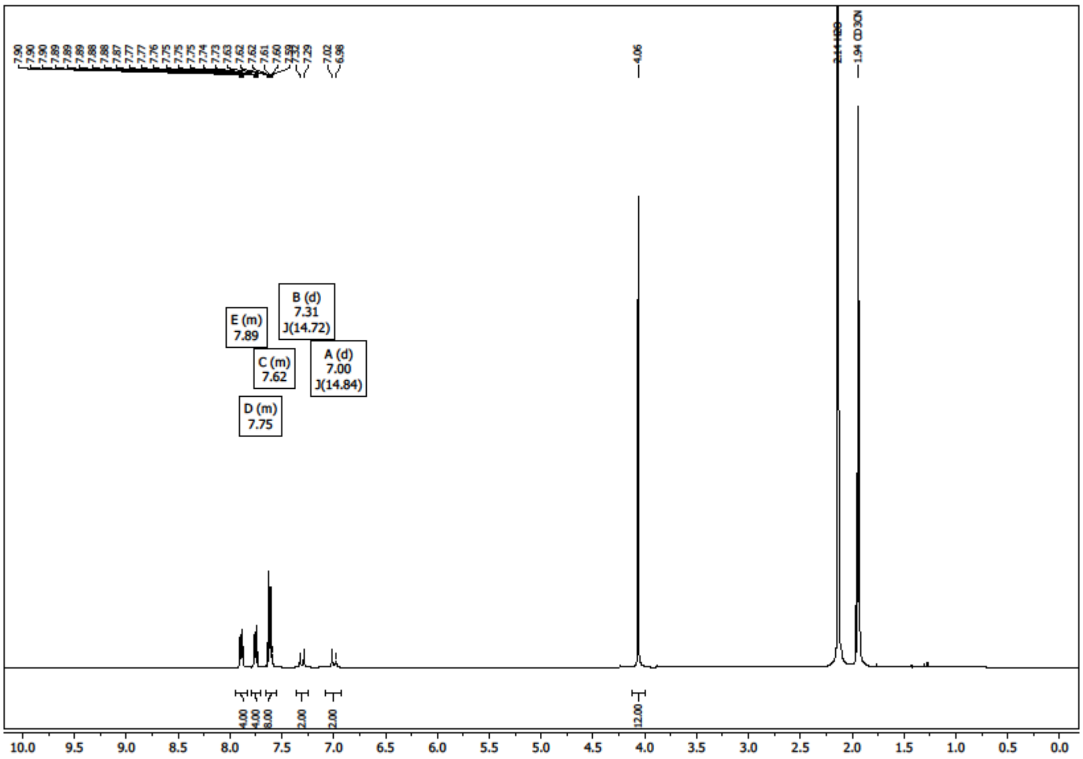

Figure S27. ^{1}H **NMR of AuC1 (CD_3CN).**

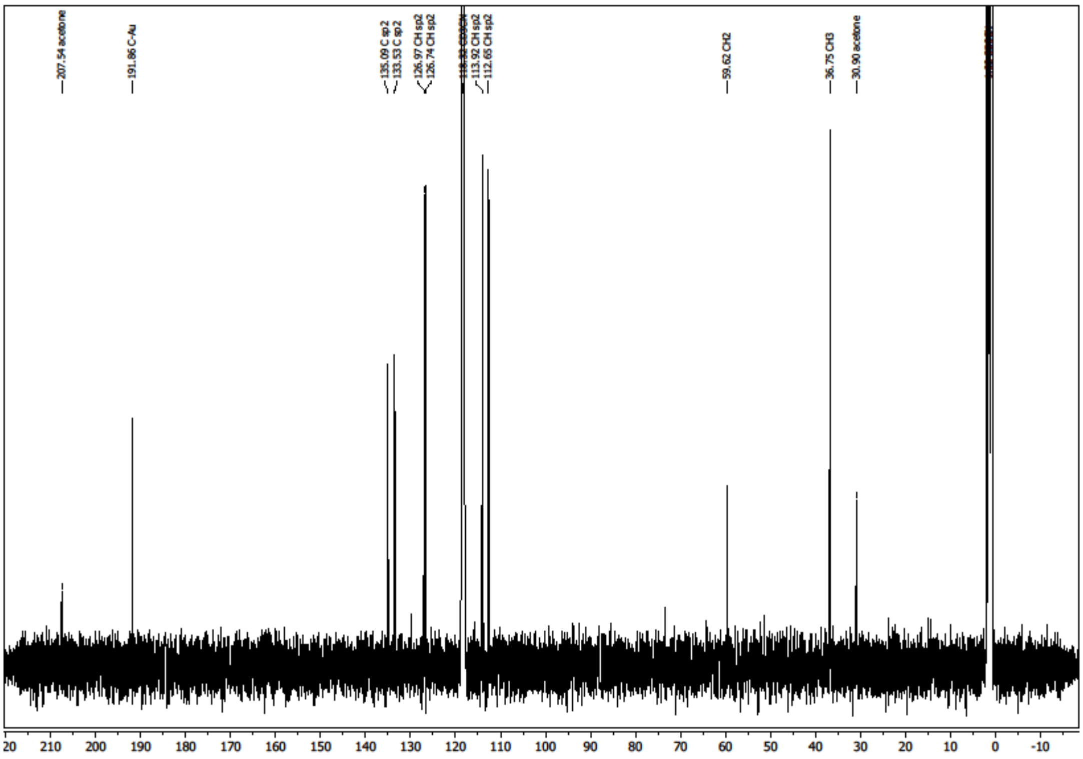

Figure S28. $^{13}C\{^{1}H\}$ **NMR of AuC1 (CD_3CN).**

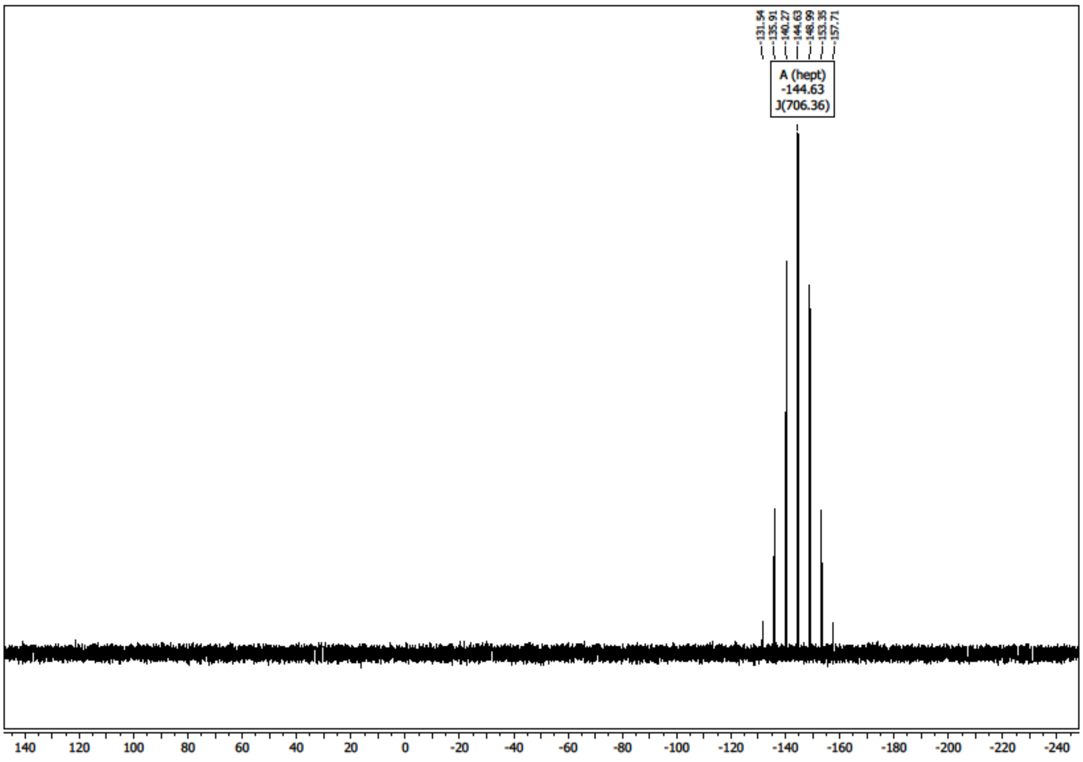

Figure S29. ^{31}**P NMR of AuC1 (**CD_3CN**).**

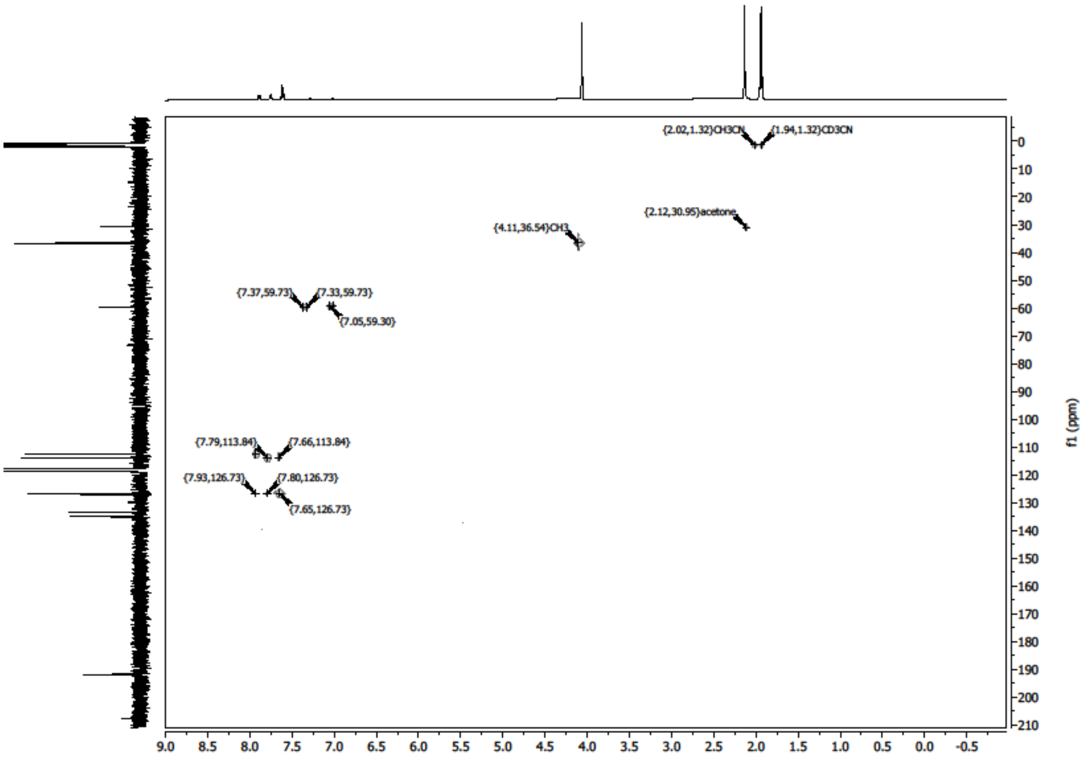

Figure S30. ^{1}**H—**^{13}**C HSQC NMR of AuC1 (**CD_3CN**).**

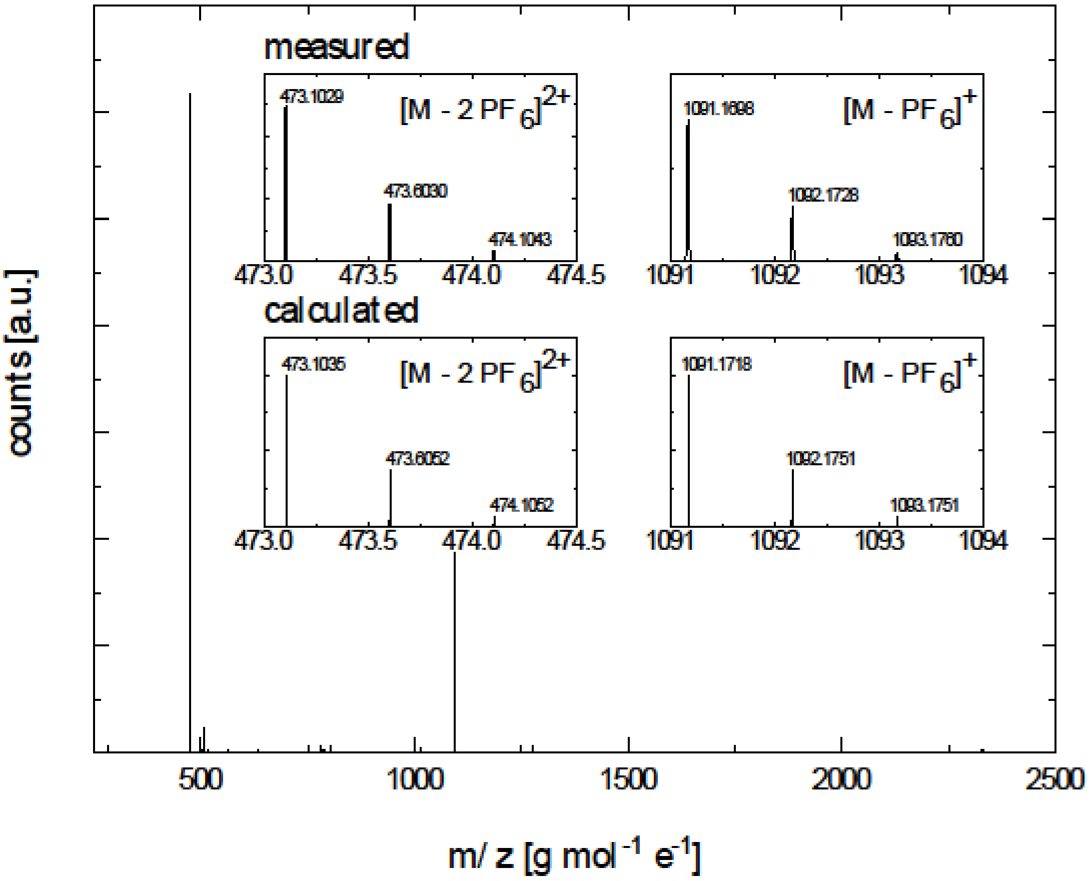

Figure S31. HR-ESI-MS of AuC1.

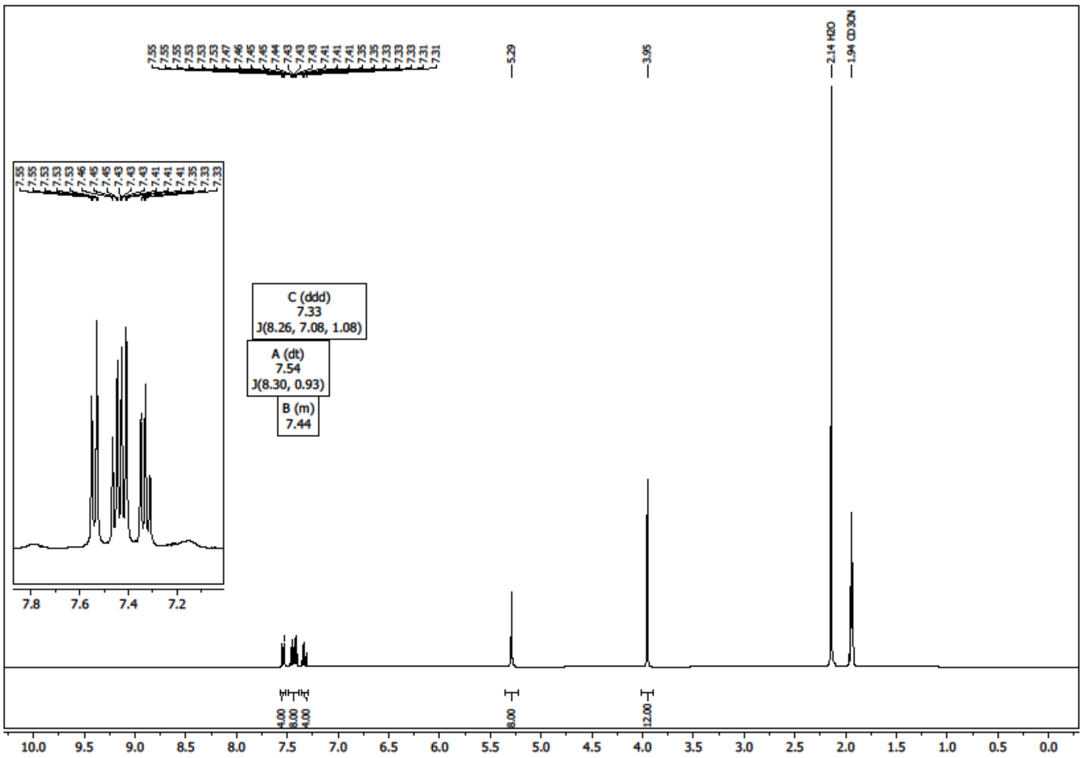

Figure S32. ^{1}H NMR of AuC2 (CD_3CN).

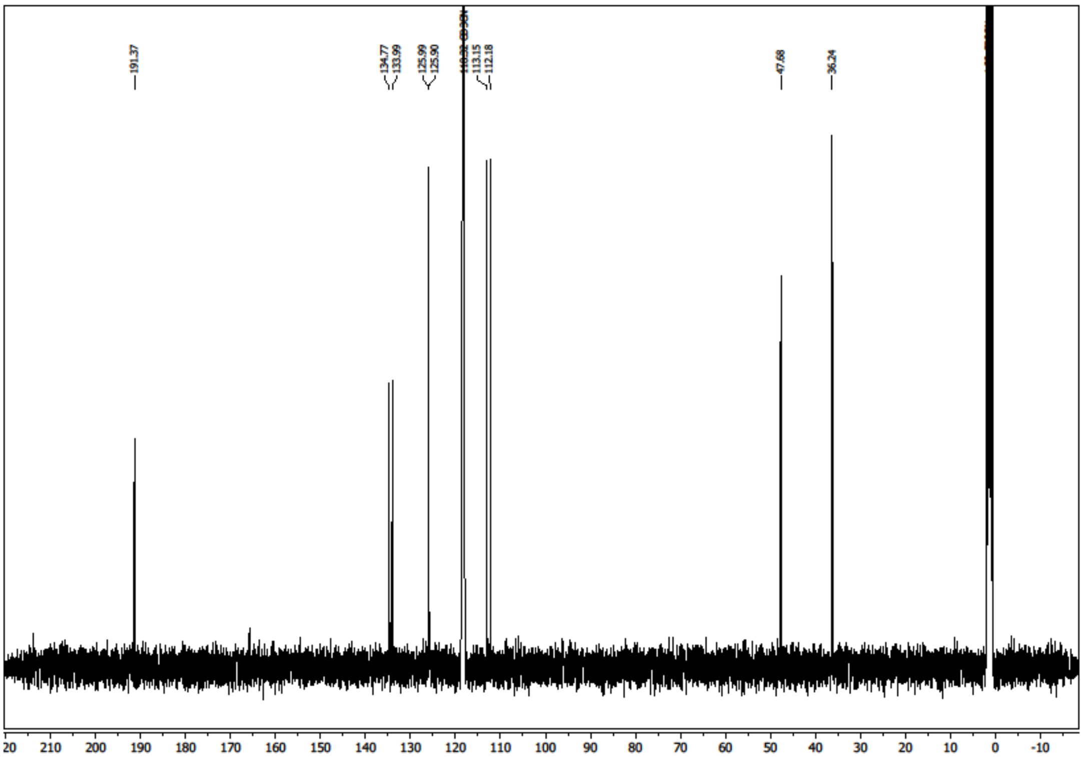

Figure S33. **$^{13}C\{^{1}H\}$ NMR of AuC2 (CD_3CN).**

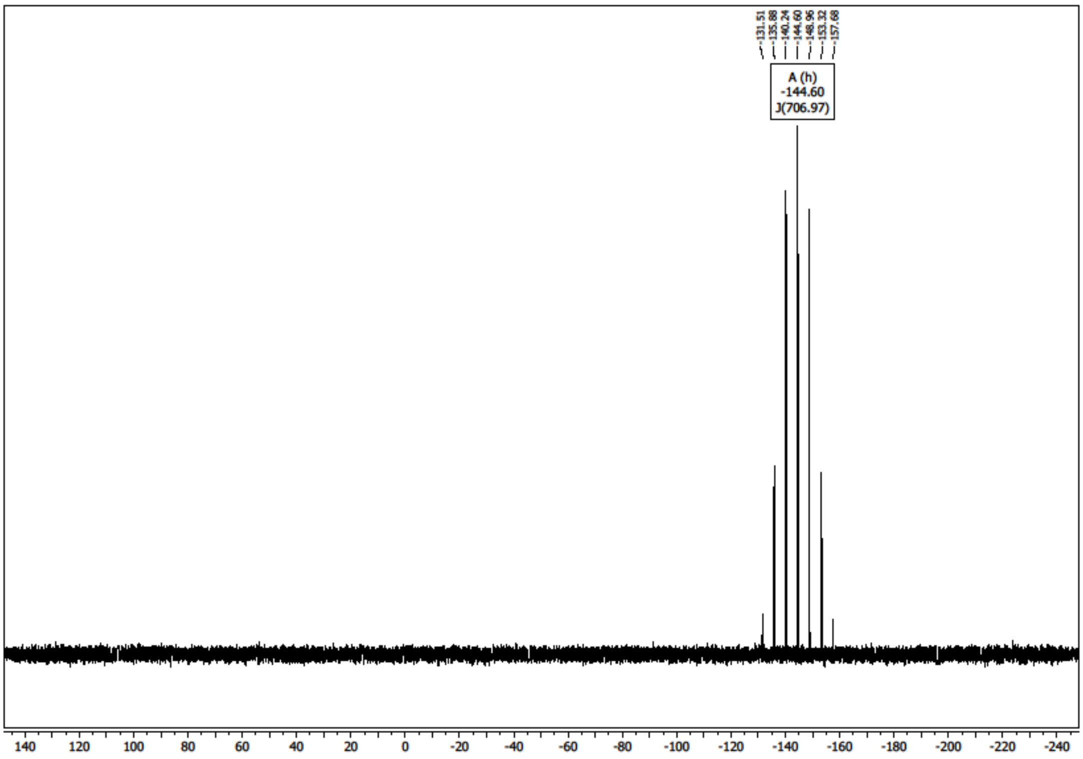

Figure S34. **^{31}P NMR of AuC2 (CD_3CN).**

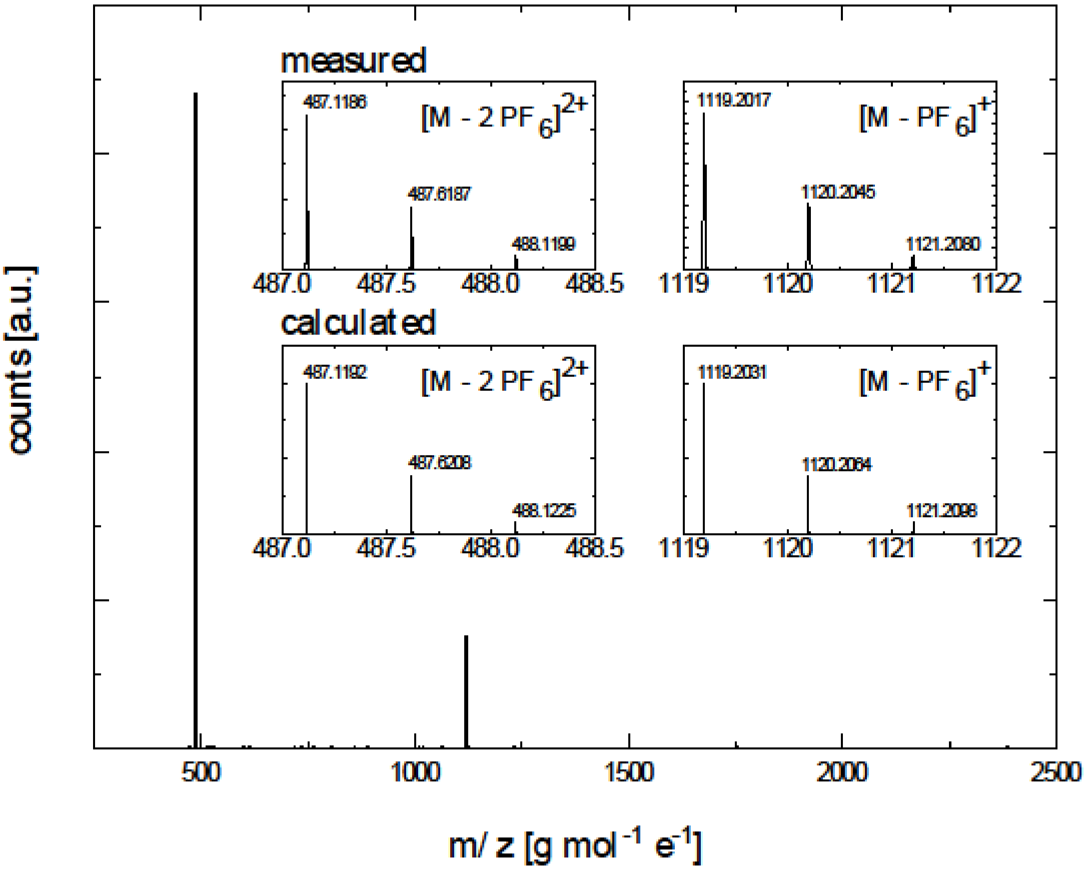

Figure S35. HR-ESI-MS of AuC2.

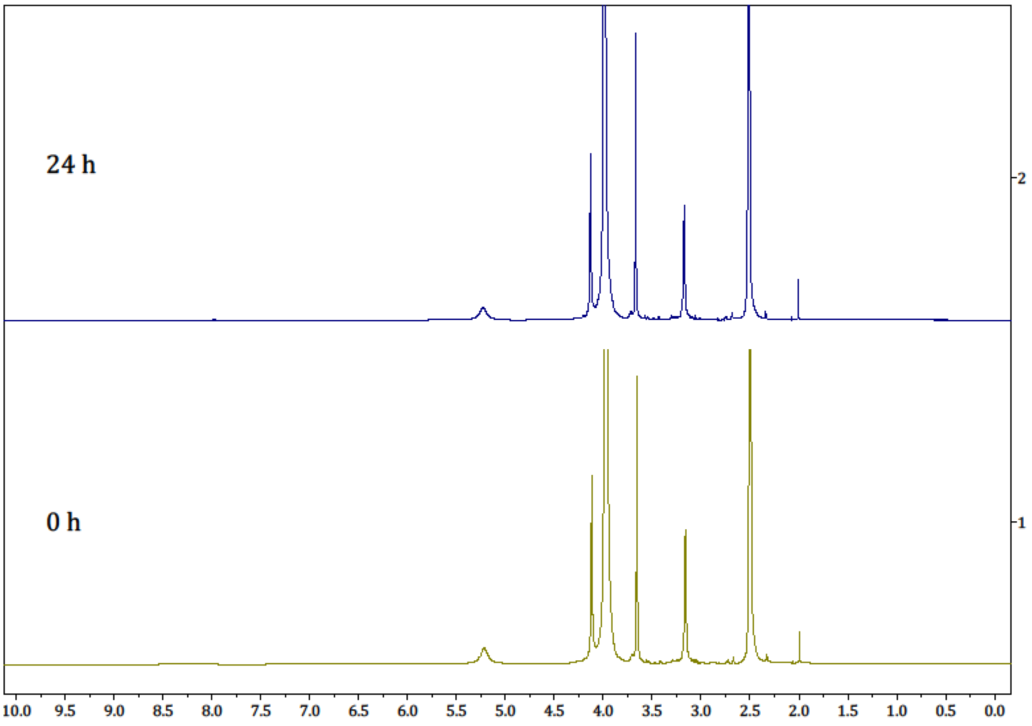

Figure S36. Stacked ^1H NMR spectra of AuB2 in D_2O/DMSO-d_6 4/1. Respective timestamps on the left.

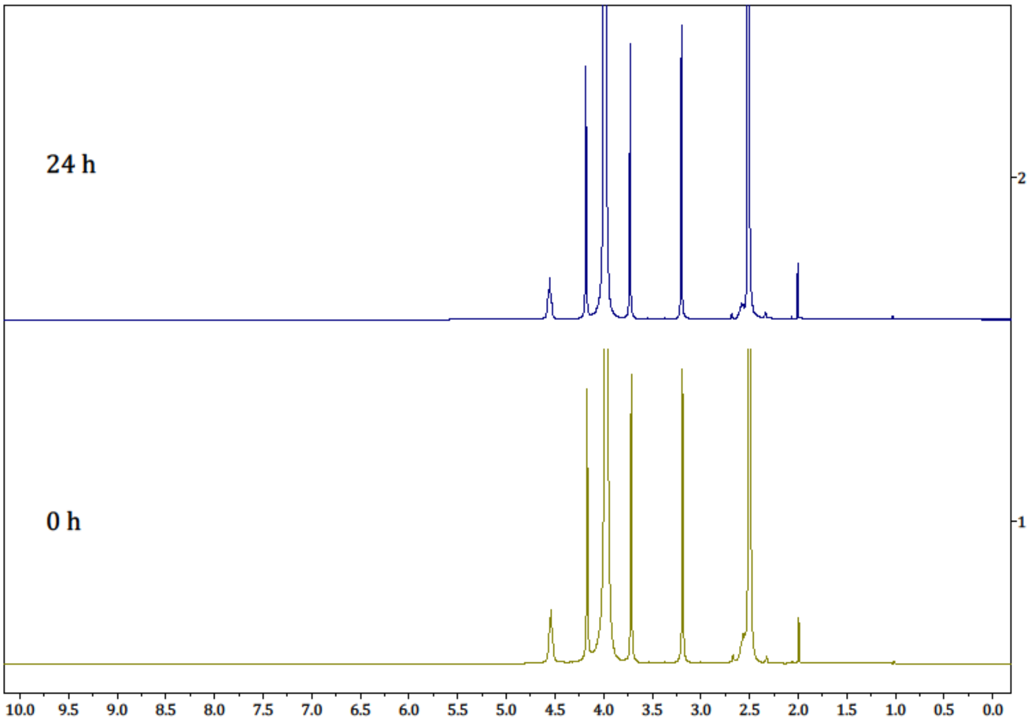

Figure S37. Stacked ^1H NMR spectra of AuB3 in D_2O/DMSO-d_6 4/1, recorded at different times.

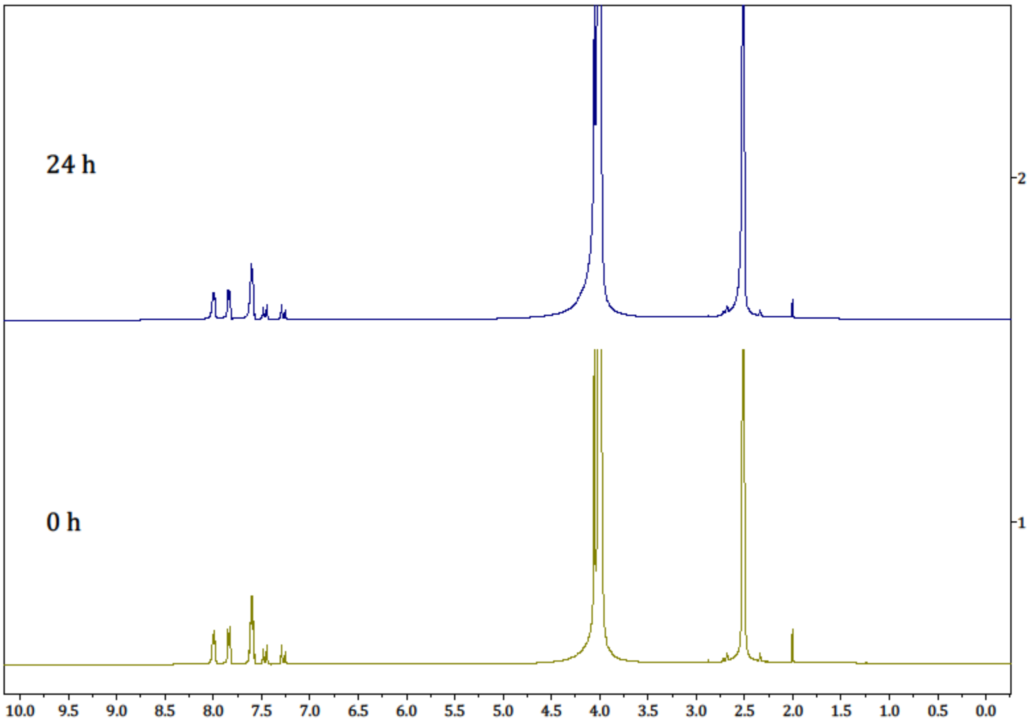

Figure S38. Stacked ^{1}H NMR spectra of AuC1 in D_2O/DMSO-d_6 4/1, recorded at different times.

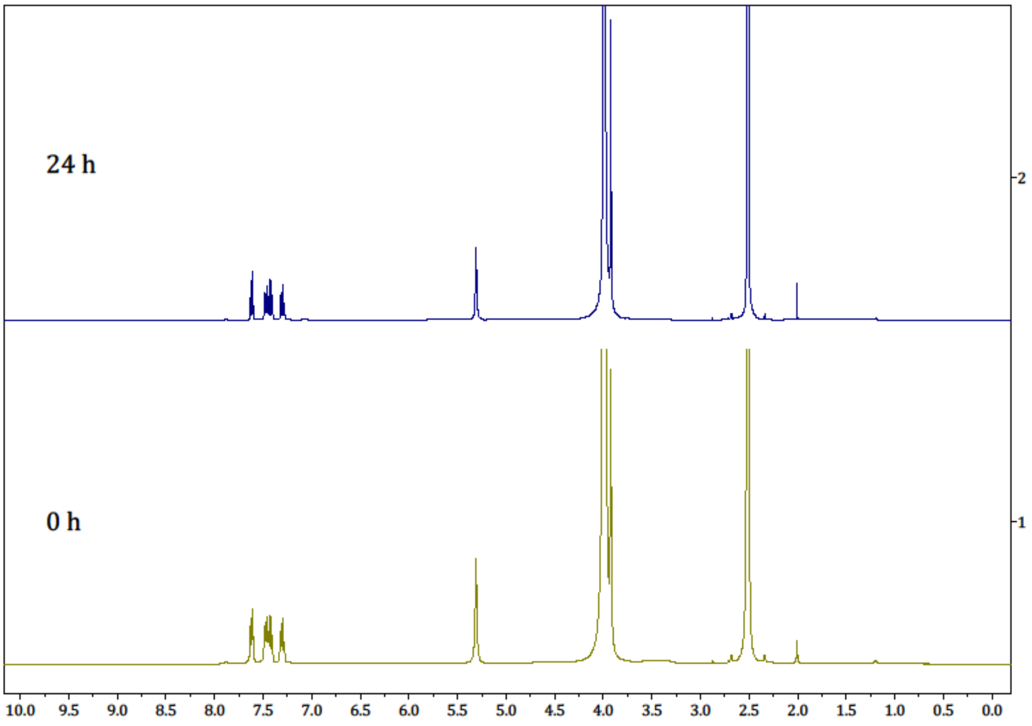

Figure S39. Stacked ^{1}H NMR spectra of AuC2 in D_2O/DMSO-d_6 4/1, recorded at different times.

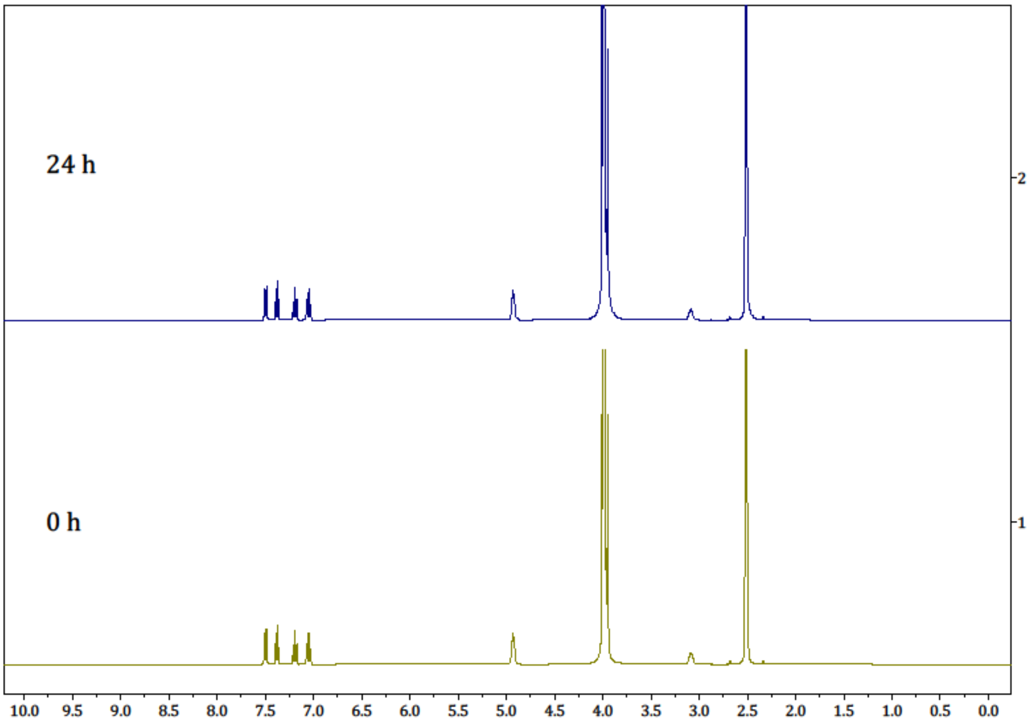

Figure S40. Stacked ^{1}H NMR spectra of AuC3 in D_2O/DMSO-d_6 4/1, recorded at different times.

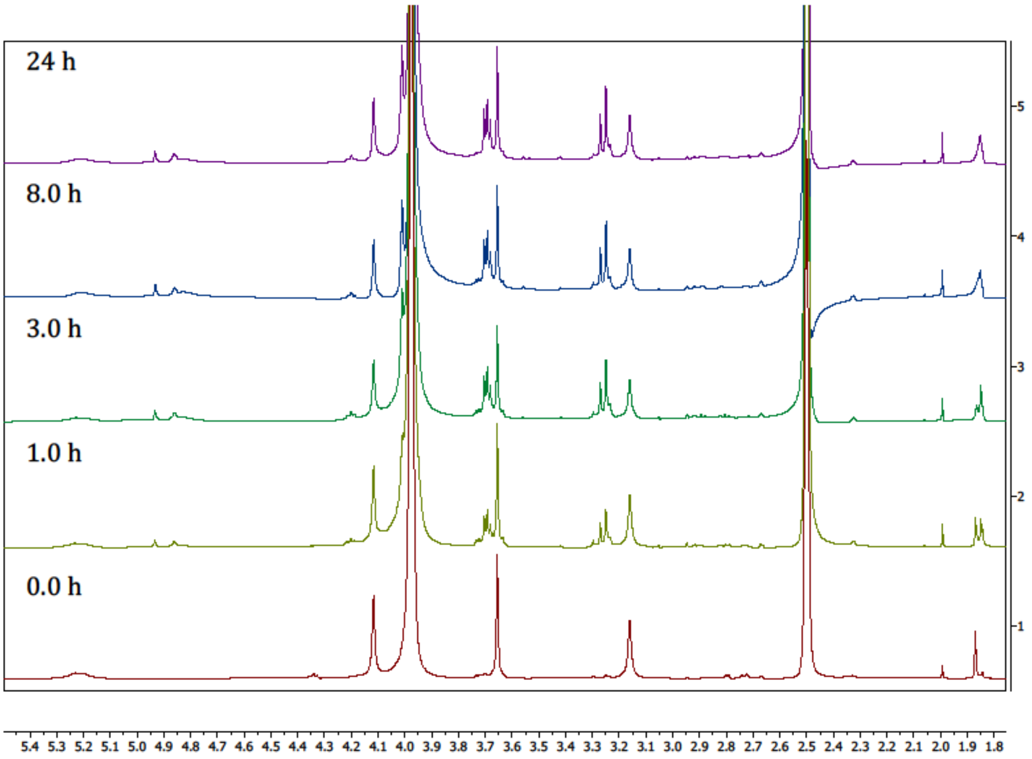

Figure S41. Stacked ^{1}H NMR spectra of AuB2 with 1 equiv. NAC in D_2O/DMSO-d_6 4/1, recorded at different times.

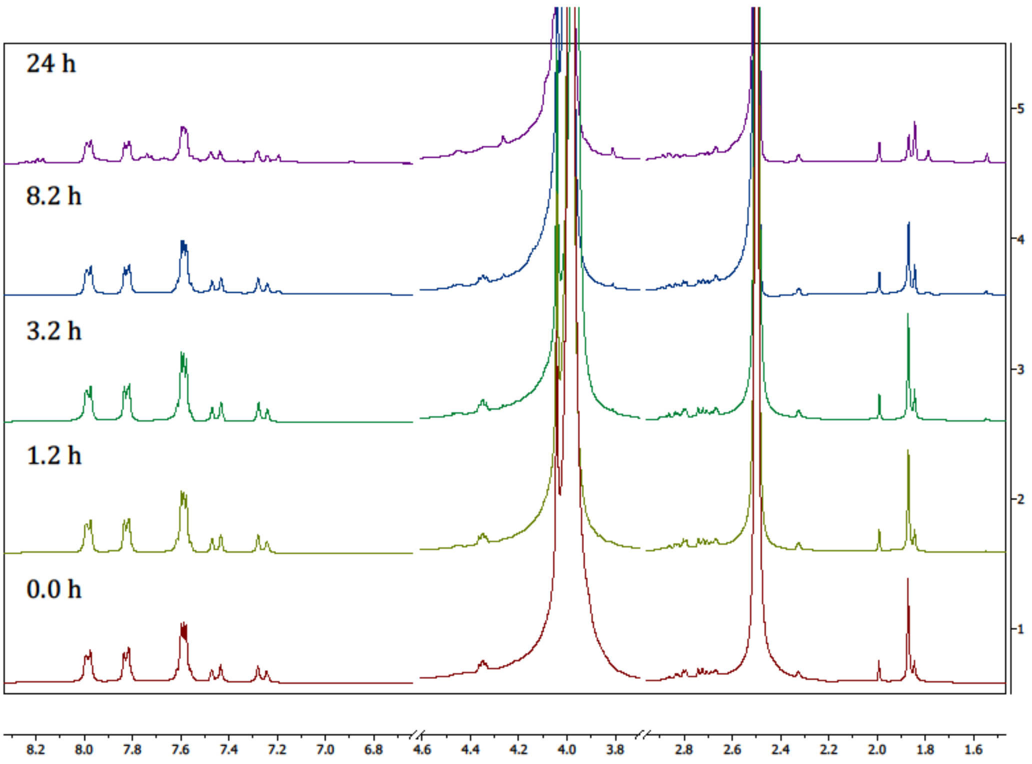

Figure S42. Stacked ¹H NMR spectra of AuC1 with 1 equiv. NAC in D_2O/DMSO-d_6 4/1, recorded at different times.

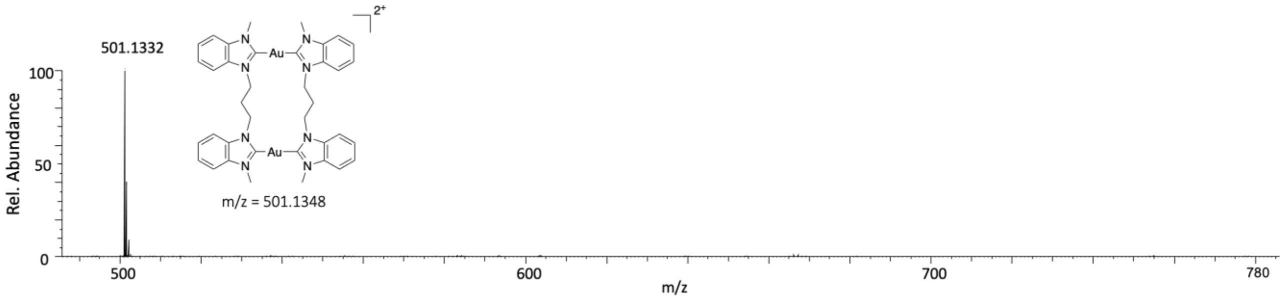

Figure S43. HR-ESI-MS of AuC1 with 1 equiv. NAC in D_2O/DMSO-d_6 4/1, reaction mixture after 24 h.

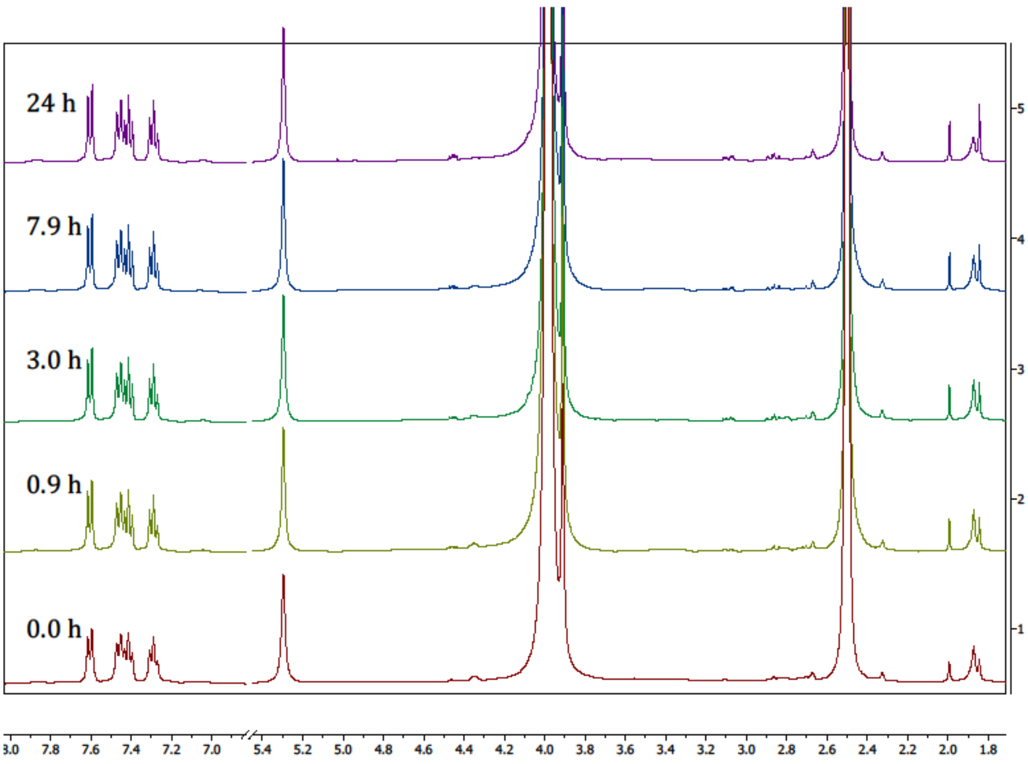

Figure S44. Stacked ^1H NMR spectra of AuC2 with 1 equiv. NAC in D_2O/DMSO-d_6 4/1, recorded at different times.

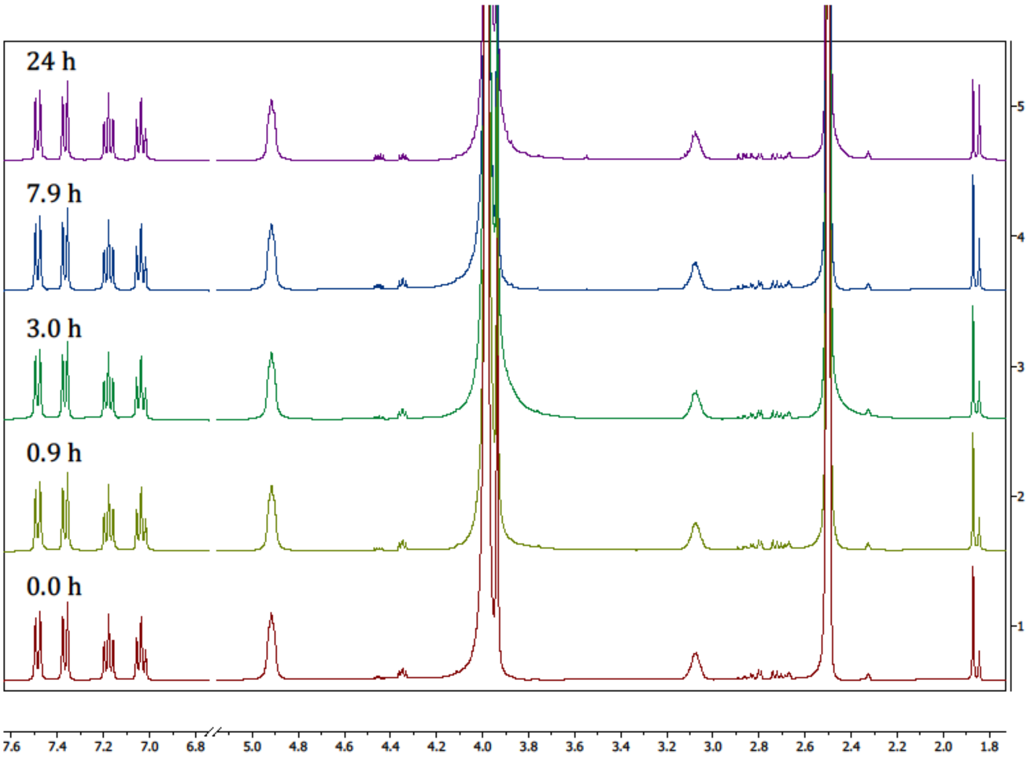

Figure S45. Stacked ^1H NMR spectra of AuC3 with 1 equiv. NAC in D_2O/DMSO-d_6 4/1, recorded at different times.

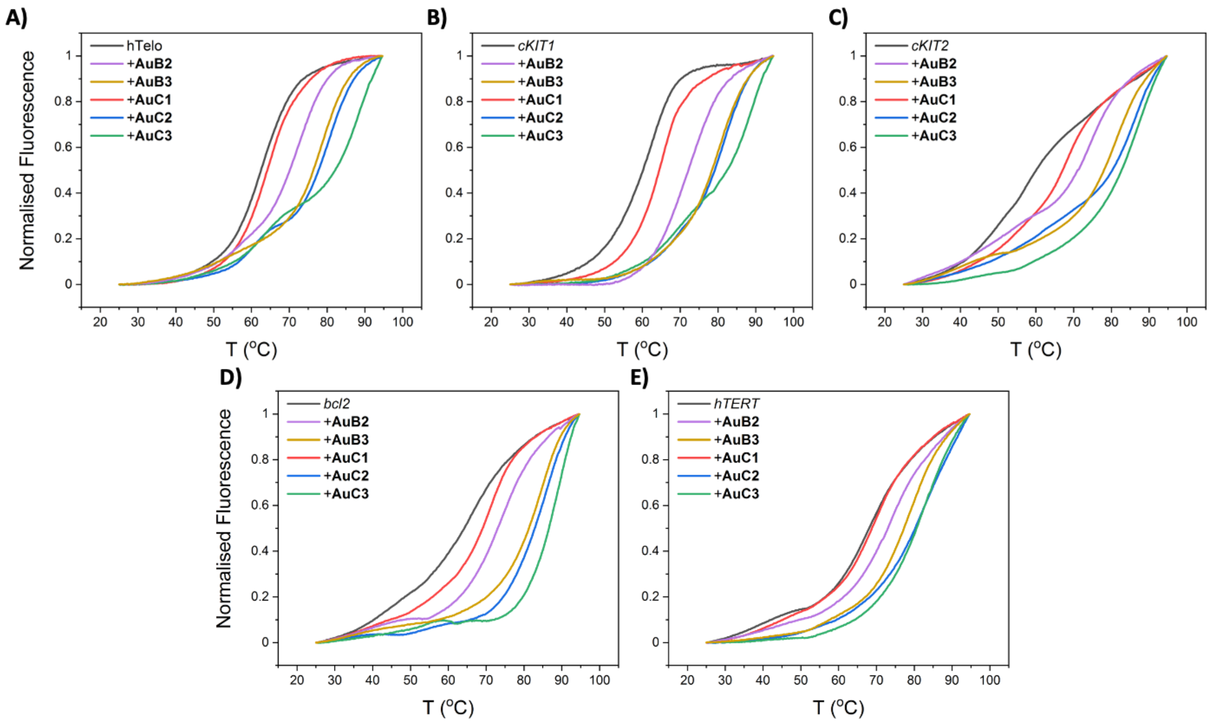

Figure S46. Representative FRET melting profiles of 200 nM hTelo (a), *cKIT1* (b), *cKIT2* (c), *bcl2* (d) or *hTERT* (e) G4-DNA alone (black lines) and in the presence of 5 equivalents of the metal compounds, in 60 mM K^+Cacodylate buffer (pH = 7.4).

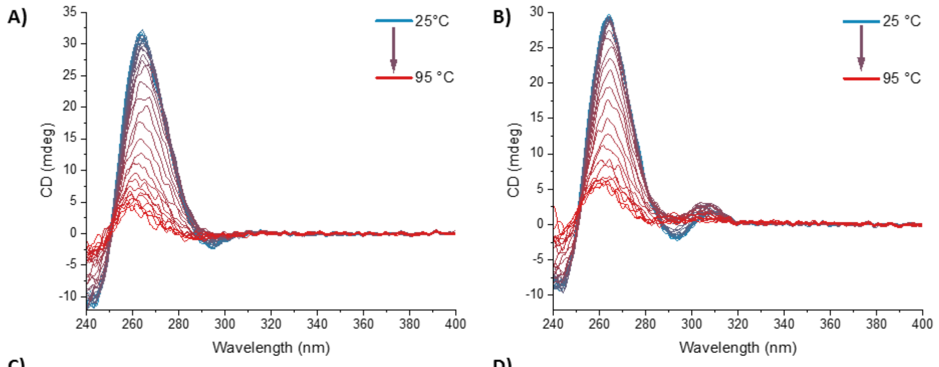

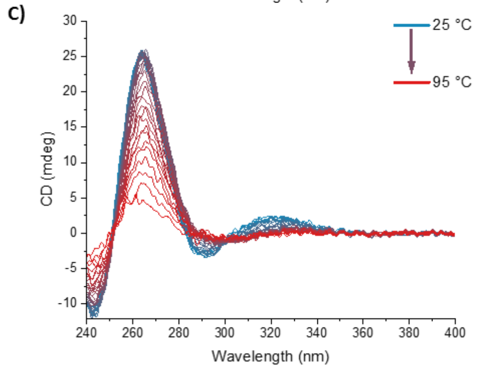

D)

	T_m	ΔT_m
cKIT1	61.4	-
**+ AuB2**	71.1	**9.7**
**+ AuB3**	75.1	**13.7**
**+ AuC3**	80.1	**18.7**

Figure S47. CD melting profiles (n=1) of 400 nM *cKIT1* solutions in the presence of 5 equiv. of AuB2 (a), AuB3 (b) or AuC3 (c) in 60 mM K^+ cacodylate buffer (pH = 7.4). Melting temperatures and their variations are reported in the table (d).

Table S1. Crystal data, data collection, and structure refinement for $\mathbf{H_2C3}^{BF4}$ (CCDC 2202916).

**Molecular Formula**		$C_{19}H_{22}B_2F_8N_4$ (H_2**C3**^{BF4})	
**Formula Weight**	[g mol $^{-1}$]	480.02	
**Crystal Dimensions**	[mm]	0.123 x 0.187 x 0.224	
**Crystal Habit**		colorless block	
**Crystal System**		monoclinic	
**Space Group**		*C2/c*	
**Unit Cell Dimensions**	[Å / °]	*a*=19.962(2)	α=90
		b=8.4954(9)	β=123.427(4)
		c=14.3428(17)	γ=90
**Volume**	[Å^3]	2030.0(4)	
**Z**		4	
**Density (calculated)**	[g cm^{-3}]	1.571	
**Absorption Coefficient**	[mm^{-1}]	0.145	
**F(000)**	[e^{-1}]	984	
**Temperature**	[K]	100(2)	
**Radiation Source**		Molybdenum *IMS microsource*	
**Wavelength**	[Å]	0.71073	
**Number of Frames**		1651	
**Exposure Time**	[h]	1.89	
**θ Range**	[°]	2.44 to 26.37	
**Index Ranges**		-24≤h≤24, -9≤k≤10, -17≤l≤17	
**Reflections Collected**		24 467	
**Independent Reflections**		2076 [*R*(int)= 0.0384]	
**Coverage**	[%]	99.9	
**Max. and min. transmission**		0.9820, 0.9680	
**Data / Restraints / Parameters**		2076 / 162 / 198	
**Goodness-of-fit on F^2**		1.051	
**Final *R* Indices**	1928 data; $I>2\sigma(I)$	R_1=0.0314, wR_2=0.0779	
	all data	R_1=0.0343, wR_2=0.0799	
**Largest Diff. Peak/Hole**	[eÅ^{-3}]	0.222, -0.209	
**R.M.S. Deviation from Mean**	[eÅ^{-3}]	0.037	

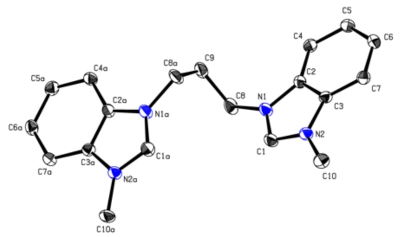

ORTEP-style representation of the cationic fragment of H_2C3^{BF4}. Thermal ellipsoids drawn at 50% probability and hydrogen atoms are omitted for clarity.

Table S2. Crystal data, data collection, and structure refinement for AuB2 (CCDC 2202917).

**Molecular Formula**		$C_{39}H_{51}Au_2F_{12}N_{17}O_9P_2$ (**AuB2** · DMF)	
**Formula Weight**	[g mol $^{-1}$]	1585.84	
**Crystal Dimensions**	[mm]	0.029 x 0.063 x 0.322	
**Crystal Habit**		colorless needle	
**Crystal System**		triclinic	
**Space Group**		$P\bar{1}$	
**Unit Cell Dimensions**	[Å / °]	*a*=11.8110(8)	α=82.712(2)
		b=15.0254(10)	β=80.622(2)
		c=18.5568(13)	γ=74.606(2)
**Volume**	[Å^3]	3120.5(4)	
**Z**		2	
**Density (calculated)**	[g cm^{-3}]	1.688	
**Absorption Coefficient**	[mm^{-1}]	4.842	
**F(000)**	[e^{-1}]	1544	
**Temperature**	[K]	100(2)	
**Radiation Source**		Molybdenum *IMS microsource*	
**Wavelength**	[Å]	0.71073	
**Number of Frames**		3656	
**Exposure Time**	[h]	18.51	
**θ Range**	[°]	1.88 to 26.02	
**Index Ranges**		-14≤h≤14, -18≤k≤18, -22≤l≤22	
**Reflections Collected**		104 429	
**Independent Reflections**		12 297 [*R*(int)= 0.0435]	
**Coverage**	[%]	100.0	
**Max. and min. transmission**		0.8720, 0.3050	
**Data / Restraints / Parameters**		12 297 / 297 / 809	
**Goodness-of-fit on F^2**		1.158	
**Δ/σ_{max}**		0.002	
**Final *R* Indices**	11 031 data; $I>2\sigma(I)$	R_1=0.0310, wR_2=0.0580	
	all data	R_1=0.0367, wR_2=0.0594	
**Largest Diff. Peak/Hole**	[eÅ^{-3}]	1.020, -1.928	
**R.M.S. Deviation from Mean**	[eÅ^{-3}]	0.110	

Table S3. Crystal data, data collection, and structure refinement for AuB3 (CCDC 2202920).

**Molecular Formula**		$C_{38}H_{48}Au_2F_{12}N_{16}O_8P_2$ (**AuB3**)	
**Formula Weight**	[g mol $^{-1}$]	1540.77	
**Crystal Dimensions**	[mm]	0.031 x 0.090 x 0.141	
**Crystal Habit**		colorless fragment	
**Crystal System**		triclinic	
**Space Group**		$P\bar{1}$	
**Unit Cell Dimensions**	[Å / °]	*a*=11.5685(12)	α=91.134(4)
		b=16.6317(18)	β=94.381(4)
		c=23.447(3)	γ=104.319(3)
**Volume**	[Å ^{3}]	4354.9(8)	
**Z**		3	
**Density (calculated)**	[g cm $^{-3}$]	1.763	
**Absorption Coefficient**	[mm $^{-1}$]	5.200	
**F(000)**	[e $^{-1}$]	2244	
**Temperature**	[K]	100(2)	
**Radiation Source**		Molybdenum *TXS* rotating anode	
**Wavelength**	[Å]	0.71073	
**Number of Frames**		3893	
**Exposure Time**	[h]	19.83	
**θ Range**	[°]	2.42 to 25.35	
**Index Ranges**		-13≤h≤13, -20≤k≤20, -28≤l≤28	
**Reflections Collected**		153 518	
**Independent Reflections**		15 931 [*R*(int)= 0.0593]	
**Coverage**	[%]	99.9	
**Max. and min. transmission**		0.8550, 0.5280	
**Data / Restraints / Parameters**		15 931 / 976 / 1265	
**Goodness-of-fit on F^2**		1.028	
**Δ/σ_{max}**		0.023	
**Final *R* Indices**	13 352 data; $I>2\sigma(I)$	R_1=0.0274, wR_2=0.0629	
	all data	R_1=0.0391, wR_2=0.0678	
**Largest Diff. Peak/Hole**	[eÅ $^{-3}$]	1.656, -1.028	
**R.M.S. Deviation from Mean**	[eÅ $^{-3}$]	0.111	

Table S4. Crystal data, data collection, and structure refinement for **AuC1** (CCDC 2202919).

**Molecular Formula**		$C_{40}H_{45}Au_2F_{12}N_9OP_2$ (**AuC1** · [MeCN, Et_2O])	
**Formula Weight**	[g mol $^{-1}$]	1351.72	
**Crystal Dimensions**	[mm]	0.064 x 0.092 x 0.123	
**Crystal Habit**		colorless rhombus	
**Crystal System**		triclinic	
**Space Group**		$P\bar{1}$	
**Unit Cell Dimensions**	[Å / °]	*a*=11.381(3)	α=85.140(9)
		b=12.909(4)	β=74.483(9)
		c=15.868(5)	γ=80.845(9)
**Volume**	[Å^3]	2235.1(11)	
**Z**		2	
**Density (calculated)**	[g cm^{-3}]	2.008	
**Absorption Coefficient**	[mm^{-1}]	6.723	
**F(000)**	[e^{-1}]	1304	
**Temperature**	[K]	100(2)	
**Radiation Source**		Molybdenum *TXS* rotating anode	
**Wavelength**	[Å]	0.71073	
**Number of Frames**		4639	
**Exposure Time**	[h]	4.85	
**θ Range**	[°]	2.37 to 26.37	
**Index Ranges**		$-14 \leq h \leq 14$, $-16 \leq k \leq 16$, $-19 \leq l \leq 19$	
**Reflections Collected**		125 603	
**Independent Reflections**		9135 [*R*(int)= 0.0457]	
**Coverage**	[%]	99.9	
**Max. and min. transmission**		0.6730, 0.4920	
**Data / Restraints / Parameters**		9135 / 43 / 623	
**Goodness-of-fit on F^2**		1.041	
**Δ/σ_{max}**		0.004	
**Final *R* Indices**	8532 data; $I>2\sigma(I)$	R_1=0.0154, wR_2=0.0378	
	all data	R_1=0.0174, wR_2=0.0389	
**Largest Diff. Peak/Hole**	[eÅ^{-3}]	0.725, -0.779	
**R.M.S. Deviation from Mean**	[eÅ^{-3}]	0.108	

Table S5. Crystal data, data collection, and structure refinement for AuC2 (CCDC 2202918).

**Molecular Formula**		$C_{38}H_{39}Au_2F_{12}N_9P_2$ (**AuC2** · MeCN)	
**Formula Weight**	[g mol $^{-1}$]	1305.65	
**Crystal Dimensions**	[mm]	0.052 x 0.086 x 0.483	
**Crystal Habit**		colorless needle	
**Crystal System**		triclinic	
**Space Group**		$P\bar{1}$	
**Unit Cell Dimensions**	[Å / °]	a=10.6058(9)	α=95.261(3)
		b=12.2839(10)	β=94.734(3)
		c=17.5892(15)	γ=110.520(3)
**Volume**	[Å^3]	2120.9(3)	
**Z**		2	
**Density (calculated)**	[g cm^{-3}]	2.045	
**Absorption Coefficient**	[mm^{-1}]	7.080	
**F(000)**	[e^{-1}]	1252	
**Temperature**	[K]	100(2)	
**Radiation Source**		Molybdenum *TXS* rotating anode	
**Wavelength**	[Å]	0.71073	
**Number of Frames**		5925	
**Exposure Time**	[h]	7.83	
**θ Range**	[°]	2.47 to 26.73	
**Index Ranges**		-13≤h≤13, -15≤k≤15, -22≤l≤22	
**Reflections Collected**		96 283	
**Independent Reflections**		9012 [*R*(int)= 0.0342]	
**Coverage**	[%]	99.9	
**Max. and min. transmission**		0.7100, 0.1310	
**Data / Restraints / Parameters**		9012 / 0 / 573	
**Goodness-of-fit on F^2**		1.158	
**Δ/σ_{max}**		0.002	
**Final *R* Indices**	8322 data; $I>2\sigma(I)$	R_1=0.0212, wR_2=0.0459	
	all data	R_1=0.0245, wR_2=0.0469	
**Largest Diff. Peak/Hole**	[eÅ^{-3}]	2.304, -1.236	
**R.M.S. Deviation from Mean**	[eÅ^{-3}]	0.096	

Table S6. Individual ΔG and CV distance values for each trajectory along with averaged Gibbs free-energy values for both metadynamics and experimental results. (T=300 K). K_b are calculated according to the equation $\Delta G° = -RT\ln(K_b)$ where T = 300 K.

Run	DG	Dist1 (Å)	Dist2 (Å)	K_b
**1**	-48.567872	9.3801	11.7079	
**2**	-47.839019	10.3558	12.111	
**3**	-33.38832	10.6174	13.2333	
**4**	-37.640519	12.029	9.9171	
**5**	-35.159826	10.0565	14.186	
**6**	-36.711671	12.4193	8.6156	
**7**	-36.540127	11.6798	9.1963	
**8**				
**9**	-39.624154	9.3641	13.3563	
**10**	-35.413376	10.2534	10.1185	
**11**	-31.028962	12.2013	11.5317	
**12**	-39.054293	10.8097	12.7508	
**13**	-36.397453	11.7796	10.3536	
**14**	-52.846012	10.1506	12.4311	
**15**	-44.443703	9.3783	11.4969	
**16**	-31.181678	9.2462	11.9768	
**17**	-34.802512	9.7268	12.4992	
**18**	-31.887519	9.8381	12.3891	
**19**	-43.956686	13.3498	9.5635	
**20**	-34.050229	10.703	12.0295	
**MetaD** *(n=19)*				
**total (kJ/mol)**	**-38.449154**	**10.7020421**	**11.5507474**	**1.1×10^6**
**stdev (kJ/mol)**	6.26540408	1.20768244	1.52509808	6.1×10^5
**Experimental** *(n=3)*				
**total (kJ/mol)**	**-33.56742**			**7.0×10^5**
**stdev (kJ/mol)**	1.69423			4.3×10^5

Movie S1. Metadynamics calculated trajectory of AuB3 interacting with the top tetrad of *cKIT1*.

AuB3 shown in licorice representation with the following colour scheme; C = teal, O = red, N = blue, H = white, Au = yellow, K = pink. cKIT1 shown in New Ribbons representation. Rendered using VMD (1).

References

(1) Humphrey, W.; Dalke, A.; Schulten, K. VMD -- Visual Molecular Dynamics. *J. Mol. Graph.* **1996**, *14*, 33–38.
